# Developing a systems-based programme theory for reducing night-time interactive electronic device use and improving sleep among adolescents

**DOI:** 10.1371/journal.pone.0359723

**Published:** 2026-10-21

**Authors:** Benjamin P. Rigby, Anne Martin, Colin B. Shore, Xueqi Wu, Carolyn Blake, Dawn Haughton, Holly Scott, Heather Cleland Woods, Rhiannon Evans, Sharon A. Simpson

**Affiliations:** 1 Population Health Sciences Institute, Newcastle University, Newcastle upon Tyne, United Kingdom; 2 School of Health and Wellbeing, University of Glasgow, Glasgow, United Kingdom; 3 Austrian National Public Health Institute, Vienna, Austria; 4 School of Health and Life Sciences, Glasgow Caledonian University, Glasgow, United Kingdom; 5 Institute for Implementation Science in Healthcare, University of Zurich, Zurich, Switzerland; 6 Psychology and Neuroscience Education Hub, University of Glasgow, Glasgow, United Kingdom; 7 DECIPHer, School of Social Sciences, Cardiff University, Cardiff, United Kingdom; LSHTM: London School of Hygiene & Tropical Medicine, UNITED KINGDOM OF GREAT BRITAIN AND NORTHERN IRELAND

## Abstract

**Background:**

Adolescent night-time use of interactive electronic devices (IEDs) is increasingly linked to poor sleep, mental health challenges, and reduced academic performance. However, existing interventions often overlook the complex systems shaping these behaviours. This study presents a first-of-its-kind systems-based programme theory for CLOCK OFF – a peer influence intervention aimed at reducing night-time IED use and improving adolescent sleep – created through novel participatory mapping activities and analyses, which involved adolescents, parents and teachers.

**Methods:**

We conducted five online participatory workshops with adolescents (aged 11–14 years), parents, and teachers across four United Kingdom schools, supported by desk-based research. Workshop activities were facilitated using StickyStudio software. Causal loop diagramming was used to identify system components and feedback loops influencing adolescent IED use and sleep. Causal loop diagrams (CLDs) were iteratively built using Kumu software, refined and validated through stakeholder input and network analysis. The final CLD informed the development of a systems-based programme theory.

**Results:**

Thirty participants (23 adolescents, 7 adults) contributed to the systems mapping process. The final CLD comprised 68 factors (*i.e.,* causal influences of night-time IED use) and 131 connections between these factors, spanning behavioural, educational, psychological, physiological, social, and technological domains. CLOCK OFF-related mechanisms were represented across a large proportion of the system’s identified feedback structures. The programme theory diagram included 72 factors and 147 feedback loops, grouped into five subsystems. These subsystems highlighted the dynamic interplay between enjoyment, overstimulation, fear of missing out (FOMO), emotional regulation, and educational stress.

**Conclusions:**

Participatory systems mapping with adolescents is a feasible and valuable method for surfacing complex behavioural drivers and informing intervention design. CLOCK OFF’s systems-based programme theory offers a novel approach for understanding and addressing adolescent night-time IED use and sleep disruption. Findings support the need for multi-actor and peer-led, multi-level interventions embedded within broader systemic strategies.

## Background

Adolescents’ use of interactive electronic devices (IEDs) at night-time, particularly for social media, is an increasing public health concern associated with sleep disruption and poorer mental health and educational outcomes [1,2]. Sleep disruption during adolescence, including delayed bedtimes, prolonged sleep onset, reduced sleep duration, difficulty falling back asleep, and poorer sleep quality, is linked to emotional dysregulation, reduced academic performance, and an increased risk of mental health difficulties [1,3–7]. Despite growing awareness of these harms, interventions targeting adolescent IED use have shown mixed effectiveness [8], often failing to address the complex social, environmental and technological factors that shape behaviour. Understanding these complexities is important for identifying, developing and targeting effective interventions that are responsive to real-world contexts [1,9].

Adolescence is characterised by increasing autonomy, heightened sensitivity to peer influence and biological changes that affect sleep timing and duration [3,5–7]. During this period, digital technologies become more embedded within social lives, with night-time IED use shaped by influences across peer, family, school and digital environments [4,5,7]. These influences operate dynamically, dampening or reinforcing behavioural patterns over time [9]. For example, social expectations surrounding online communication may encourage night-time device use, disrupting sleep and subsequently affecting mood, wellbeing and daily functioning, which in turn can further reinforce digital engagement [1,6,9]. However, parents and teachers also continue to play a critical role during adolescence; therefore, integrating their perspectives is essential for a comprehensive understanding of behaviour [10,11]. Approaches that focus on isolated determinants of behaviour risk providing only partial explanations of adolescents’ night-time IED use and sleep. This highlights the need for intervention development approaches that account for different actors, complexity, context and interactions within behaviour change systems [12].

Responding to complexity has become central to intervention development. The updated UK Research and Innovation Medical Research Council (MRC) framework for developing and evaluating complex interventions emphasised the importance of considering how interventions interact with context, how mechanisms of change are generated and how stakeholder perspectives are incorporated throughout the intervention lifecycle [12]. Although complexity should be considered throughout, the development phase is particularly important for surfacing assumptions about how change is expected to occur, identifying potential leverage points and ensuring that interventions are responsive to the systems in which they will be implemented.

One approach to addressing complexity during intervention development is the use of programme theory, which provides an explicit account of how an intervention is expected to produce its intended outcomes, including mechanisms through which change may occur and the contextual factors that influence these processes [12]. However, conventional programme theory can struggle to represent the dynamic and interactive nature of real-world settings. A systems perspective addresses this limitation by conceptualising interventions as events introduced into dynamic systems, where outcomes emerge through adaptation and interactions between intervention components, contextual influences and feedback processes [12,13]. From this perspective, understanding change requires attention not only to intervention activities, but also the wider systems in which behaviours are embedded.

Participatory systems mapping is considered a key approach to operationalising a systems perspective, involving stakeholders in visualising the components of a system and the relations between them [14,15]. A range of systems mapping methods exist, including fuzzy cognitive maps and Bayesian networks; however, causal loop diagrams (CLDs) are used most commonly in public health research to represent dynamic systems [14,16,17]. System maps support the understanding of the systems in which behaviours are embedded and provide a foundation for theorising how change may occur through intervention.

Building on system maps (especially CLDs), systems-based theory of change (ToC) approaches have emerged in response to limitations of conventional ToC models, which are often criticised for representing change as linear and static [15,18,19]. Systems-based ToC retains core elements of inputs, activities, mechanisms and outcomes, but explicitly incorporates feedback loops, contextual mechanisms and interactions between components, providing a more dynamic representation of change within complex systems [15,18,19]. While the concept of systems-based ToC mapping is established, in line with guidance on complex intervention development [12], we adopt the term *systems-based programme theory* to emphasise its explanatory focus, and the interdependent relationships between intervention components, contexts, mechanisms and outcomes. Despite burgeoning interest in systems mapping, the development of systems-based programme theories in public health remains under-examined, with limited methodological guidance and few empirical examples. This gap is particularly evident in research involving children and adolescents. Recent reviews of public health systems mapping studies [14,16,17] identified relatively few applications within these populations, although existing work spans topics such as neonatal mortality, health inequalities, asthma, obesity, fruit and vegetable intake, drink-driving and environmental health [20–36]. None addressed IED use or sleep. More recent studies have demonstrated the value of combining participatory approaches and systems thinking with young people, including the use of CLDs to explore energy balance-related behaviours, identify leverage points and inform action development [37–40]. Collectively, this work has demonstrated that children and adolescents can meaningfully contribute to systems-orientated research and intervention development.

A notable contribution was the study by Heemskerk et al. [9], which engaged adolescents in group model building to explore the dynamics of sleep health. The study identified six interacting subsystems (i.e., distinct but interconnected groups of factors operating within a wider system) influencing sleep (school; mental wellbeing; family and home; health behaviours and leisure; personal; digital environment) and challenged assumptions about blue light exposure as a driver of poor sleep. While the action scales model [41] was used to identify levels of potential intervention, the study did not examine how proposed actions might generate change within the system, nor did it provide detailed reflection on the nature or depth of adolescent involvement. More broadly, although participatory systems studies have advanced understanding of complex health behaviours and informed action development, there remains limited translation of systems insights in systems-based programme theories that explicitly theorise mechanisms of change within adolescent systems. Methodological guidance also remains limited, particularly regarding how systems methods can be adapted to suit adolescents’ developmental stages and the practical realities of working within schools and families [29,35,42]. Additional challenges include assumptions about who qualifies as an ‘expert stakeholder’ [43,44], and concerns regarding the accessibility of systems methods beyond trained researchers [45]. Addressing these gaps is important for strengthening applications of systems approaches to intervention development and generating interventions that are theoretically-informed, contextually responsive and grounded in adolescents’ lived experiences.

### Study aims

To address these gaps, this study adopted a systems perspective to support the development of CLOCK OFF, a school-based intervention designed to reduce adolescents’ night-time IED and social media use and improve sleep through educational and peer-influence strategies. At the time of the study, CLOCK OFF was in the intervention development phase, and a systems-informed participatory process was used to identify potential intervention targets, mechanisms of change and implementation considerations.

The primary aim was to develop an initial systems-based programme theory for CLOCK OFF using participatory systems mapping with adolescents, parents and teachers. This included exploring and validating these stakeholders’ perceptions of the system dynamics linking night-time IED use and sleep, particularly the digital environment and its social characteristics. The secondary aim was to generate methodological reflections on the use of participatory systems mapping with adolescents to inform intervention development. The study was guided by the following research question: how can participatory systems mapping inform the development of an initial systems-based programme theory for CLOCK OFF, and what methodological insights can be generated from this process?

## Materials and methods

### CLOCK OFF

This paper presents findings from the CLOCK OFF study, which was conducted with adolescents aged 11–14 years, as well as school staff and parents, in Scotland and Wales, United Kingdom (UK). Consistent with guidance on complex intervention development [12], CLOCK OFF was developed by using multiple sources of evidence, including existing literature and theory, stakeholder perspectives (including participants and charity partner experiences) and the empirical insights presented in this paper. An overview of its components are provided in Additional File 1. The systems mapping process was used to identify potential key influences on adolescents’ night-time IED use and sleep, potential entry points for intervention, mechanisms of change and implementation conditions, supporting the development of an initial systems-based programme theory. This programme theory informed the selection, adaptation and integration of evidence-based intervention and behaviour change techniques, drawing on the *Sound Sleep* programme [46], the *#SleepyTeens* programme [47,48] and the *ASSIST* approach [49–51]. See Additional File 2 for an overview of these programmes. Rather than combining these interventions, CLOCK OFF adapted relevant components within a systems-informed programme theory derived from the systems mapping process. Harnessing the importance of peer dynamics in adolescence and digital environments [52–55], the resulting intervention was a six-to-eight-week school-based programme combining teacher-led lessons and trained peer supporters to promote healthy sleep habits and reduce night-time IED use. Although delivered as a discrete school-based intervention, the intervention is explicitly situated within the wider system shaping adolescent night-time IED use and sleep, with its programme theory describing how the intervention components are expected to interact with existing system dynamics to produce change. Findings from the feasibility and acceptability testing will be reported in a forthcoming publication.

### Study design

This primarily qualitative study comprised a series of online systems mapping and validation workshops, supplemented by extensive desk-based research. Details of each phase are provided in the latter portions of this methods section. The mapping activities formed one component of a broader intervention development process for CLOCK OFF. This paper focuses specifically on the participatory systems mapping and programme theory development stages; findings from other intervention development activities will be reported separately. Online workshops enabled rapid, accessible participation by adolescents and adult stakeholders while accommodating school timetables and developmental needs, and generated rich causal data [15]. Ethics approval was granted by the University of Glasgow College of Social Sciences ethics committee (Ref: 400210152).

### Participant recruitment

In collaboration with the Schools Health and Wellbeing Improvement Research Network (SHINE) in Scotland and the School Health Research Network (SHRN) in Wales, whose member schools had cited sleep and mental health as priority issues, we aimed to recruit 20–30 adolescents and 10–20 adult stakeholders. Participants were recruited between 5 April 2022 and 31 May 2023, reflecting the sequential recruitment for mapping and validation workshops, and the scheduling of data collection around school timetables. Email invitations were sent via SHINE and SHRN to affiliated schools. Four schools expressed interest in participation (two per nation). A briefing was held with designated school contacts to discuss the research and respond to any queries. School contacts were asked to identify adolescents to take part (aged 11–14 years; no exclusion criteria), briefed on how to prepare the classroom for workshops, and asked to support recruitment by approaching appropriate adult stakeholders (*i.e.,* teachers at a recruited school, or parents or guardians of children aged 11–16 years at a SHINE or SHRN affiliated school). All participants provided written informed consent, with adolescents’ forms co-signed by a parent or guardian.

### Data collection

Data were collected between October 2022 and May 2023. Table 1 summarises the study’s data collection components. This paper focuses on the systems mapping and programme theory development stages, which formed part of the wider CLOCK OFF intervention development process. Details of the intervention integration and refinement workshops will be reported elsewhere.

**Table 1:** Summary of the CLOCK OFF study data collection components (19 workshops) and timeline.

Date	School 1 (S) – adolescents	School 2 (W) – adolescents	School 3 (S) – adolescents	School 4 (W) – adolescents	Parents and teachers (S/W)
Oct 2022	CLD building workshop^*^	CLD building workshop^*^			
Nov 2022	*ASSIST* integration workshop *Sound Sleep and Sleep Teens* integration workshop Programme theory review workshop	*ASSIST* integration workshop *Sound Sleep and Sleep Teens* integration workshop Programme theory review workshop	CLD building workshop^*^ ASSIST integration workshop *Sound Sleep and Sleep Teens* integration workshop Programme theory review workshop		
Dec 2022					
Jan 2023				Two CLD validation workshops^*^	
Feb 2023				*ASSIST* integration workshop *Sound Sleep and Sleepy Teens* integration and programme theory review workshop	
Mar 2023					
Apr 2023					*ASSIST* and *Sound Sleep* integration workshop *Sleepy Teens* integration and programme theory review workshop
May 2023					CLD validation workshop^*^
	**Total = 4 workshops**	**Total = 4 workshops**	**Total = 4 workshops**	**Total = 4 workshops**	**Total = 3 workshops**

**Legend:** S = Scotland, W = Wales; ^*^ Workshops covered in this paper.

## Causal loop diagramming

### General overview of the workshops

Causal loop diagramming was used to explore the complex, dynamic influences of adolescent night-time IED use on sleep and mental health, and to inform intervention development and programme theory. Two types of online workshops were conducted via Microsoft Teams: (1) map-building workshops to identify core system components; and (2) validation workshops to assess whether evolving CLDs reflected participants’ experiences, identify omissions and refine researcher interpretations. Each 90-minute session was scheduled to suit participants (*e.g.,* during school hours or evenings) and recorded using Microsoft Teams’ integrated function. This rapid format contrasted with previous approaches that adopted more established group model building processes [29,35].

Workshops were facilitated by CS, with systems mapping by BR or CB (additional notes taken by GD or CE). Sessions included an introduction, research activities with a break, and a feedback exercise (see Additional File 3 for detailed guides). A teacher was present during adolescent workshops for technical and behavioural support. Participants received a £10 gift voucher per session. Desk-based research was conducted before, between, and after workshops. Figure 1 outlines the key steps.

**Fig 1:**
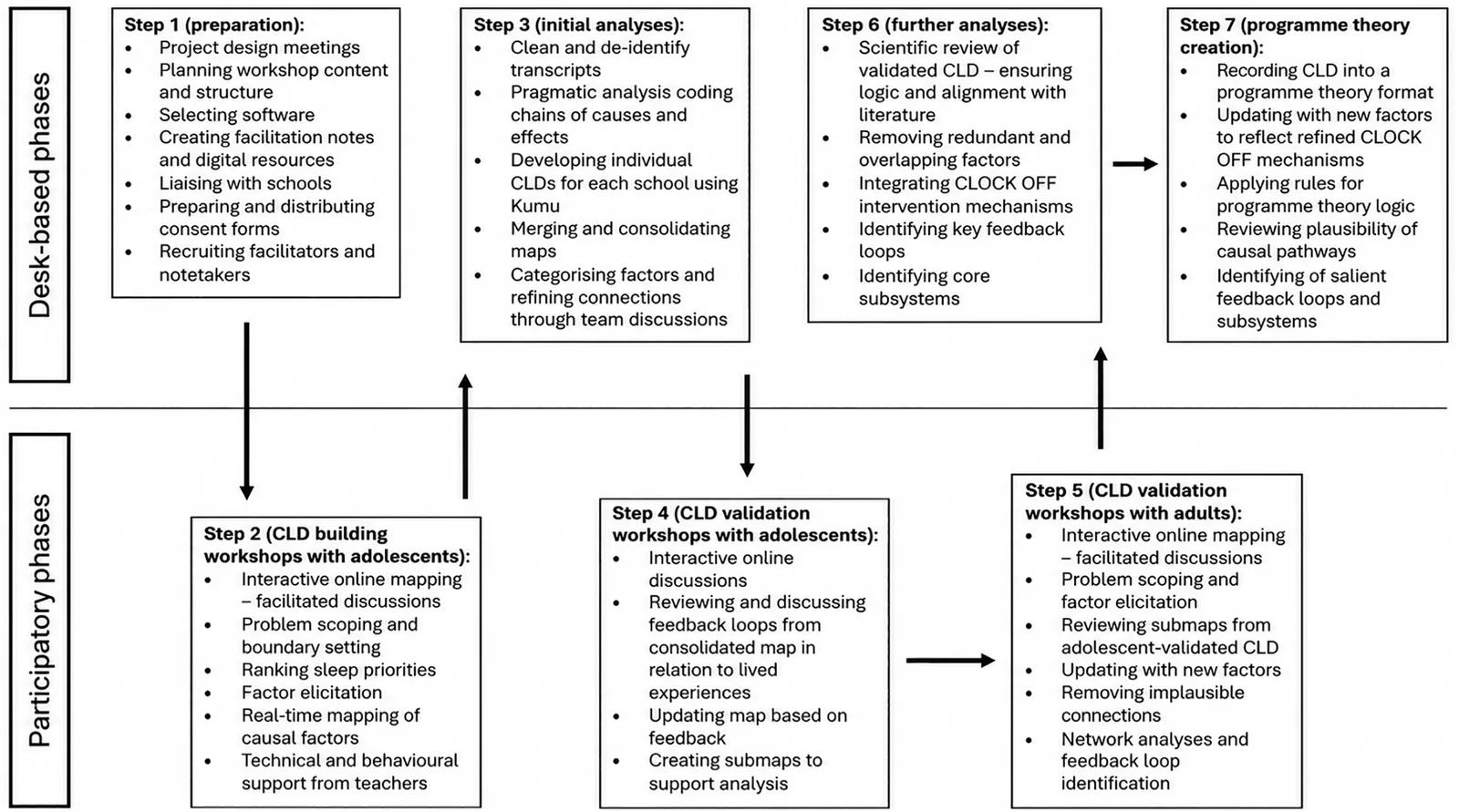
Overview of the causal loop diagramming process.

### Step 1: Preparation (desk-based phase)

Workshop preparation was iterative and spanned several months. The research team held project design meetings involving three experienced systems mapping researchers (BR, CB, AM) to determine the approach. Key preparatory tasks included planning workshop content, selecting appropriate software for adolescent engagement, developing facilitation materials, liaising with schools, managing consent procedures, and recruiting facilitators and notetakers.

### Step 2: CLD building workshops (participatory phase)

Three schools (Schools 1–3) participated in online CLD building workshops to explore adolescents’ perspectives on the factors influencing night-time IED use, and the relations between these factors and sleep-related outcomes. The workshops were guided by the overarching systems question: *what factors influence adolescents’ use of interactive electronic devices at bedtime, and how do interactions between these factors affect sleep?* Sessions were delivered via Microsoft Teams, using Microsoft PowerPoint and StickyStudio (Kumu Inc.) to support interactive mapping and data capture.

Workshops began with problem scoping and boundary setting, including participants defining ‘bedtime’ as the hour before sleep onset. This often overlooked step in systems mapping helped ensure relevant system influences were captured and the project remained tractable within the limited timeframe [56]. Adolescents ranked sleep priorities (*e.g.,* sleep duration, feeling rested) using dot voting exercise [57], followed by a structured factor elicitation process. Participants responded to four guiding questions used as prompts for factor elicitation, which centred on what devices are used for and why, the things that stop device use, and factors affecting sleep (see Additional File 3). Generated factors were recorded on digital sticky notes. These were reviewed in real time, with duplicates removed, similar factors grouped (*e.g.,* social media platforms collapsed into a single factor), and labels refined for clarity. At this stage, factors were not yet expressed as system variables [17,18].

The facilitator introduced causal relations and guided participants in identifying connections between factors. Each proposed relation was discussed in terms of directionality and influence (*e.g.,* whether factor A influences factor B, vice versa, or both). Agreed connections were mapped live in StickyStudio by the system mapper, forming the initial structure of a CLD. Figure 2 shows an example output.

**Fig 2:**
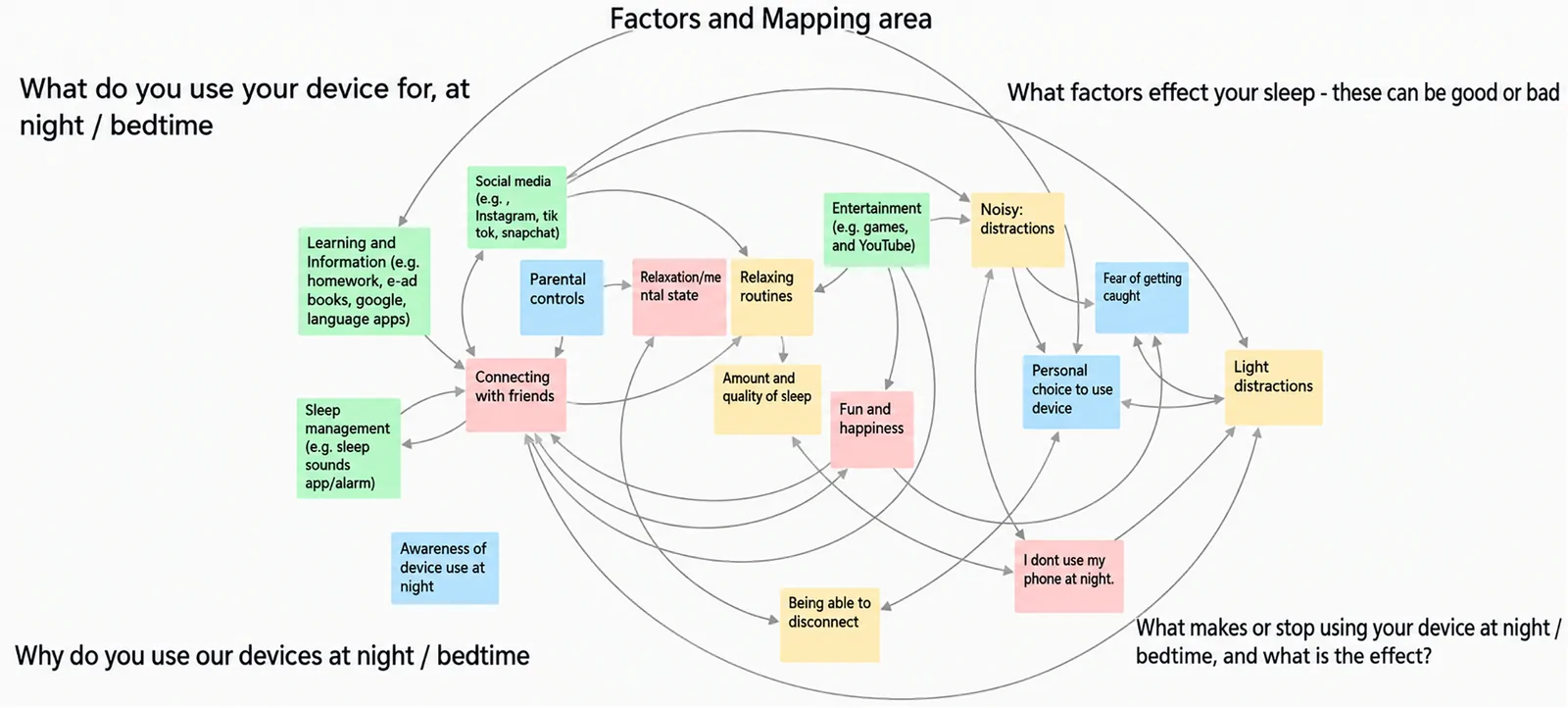
Initial CLD building workshop output.

### Step 3: Initial analyses – map refinement and consolidation (desk-based phase)

Analysis began after the first CLD-building workshop, allowing refinement of the workshop guide for subsequent iterations. Most analysis was completed while schools engaged in later intervention development workshops. Initially, data from each school were analysed separately to refine three individual CLDs.

Transcripts from MS Teams were cleaned and de-identified by CS, then analysed alongside StickyStudio data using a ‘pragmatic causal chain analysis’, adapted from Kim *et al.* [58]. First, transcripts were coded to identify different uses of IEDs (*e.g.,* social media use, messaging, gaming, entertainment and school-related activities). Second, transcripts were coded according to: (1) factors that change night-time IED use; (2) effects of IED use; (3) factors that change sleep quality/quantity; and (4) effects of sleep changes. For each factor, chains of causal influence were mapped, extending two-to-four factors from central nodes (*i.e.,* ‘use of device at night-time’ or ‘sleep quality/quantity’), with directionality noted. For example, ‘education from parents/guardians’ increased ‘awareness of the effects of device use at bedtime’, causing IED use to decrease. Coded data were transferred into a cause-and-effect grid in Microsoft Excel (see Fig 3), supplemented by StickyStudio data. This novel adaptation enabled rapid, rigorous formatting of complex data for CLD development, overcoming time constraints during workshops.

**Fig 3:**
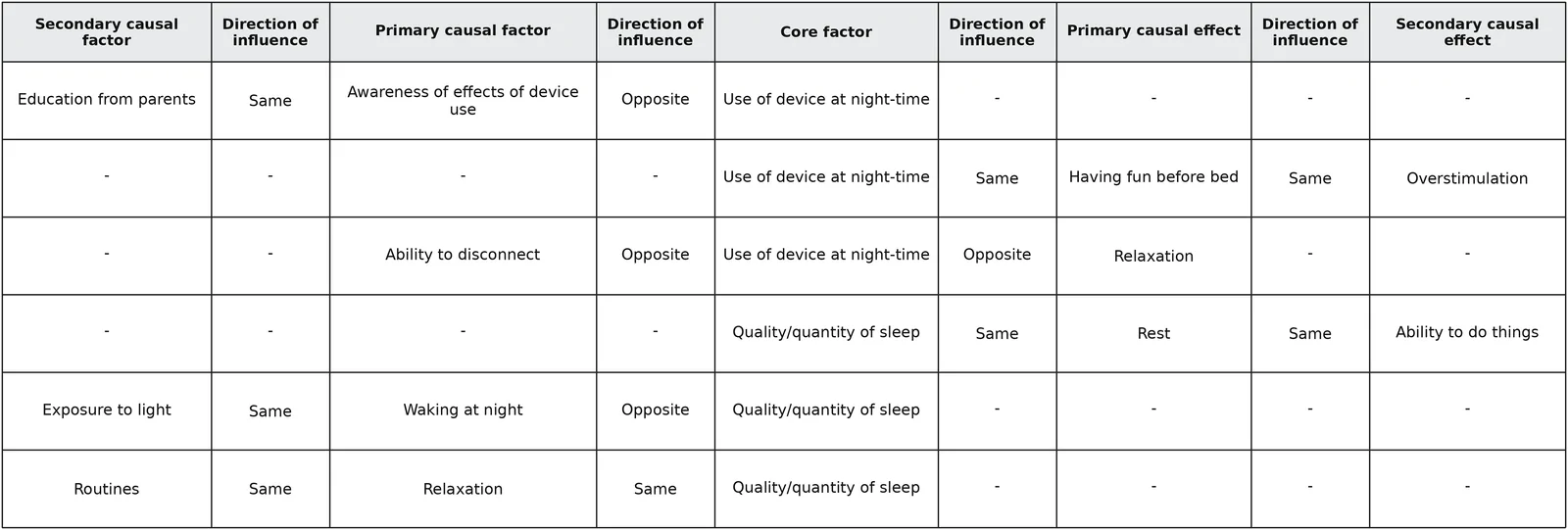
Example of abridged cause-and-effect grid created using data from School 2.

Individual CLDs for each of Schools 1–3 were manually created in Kumu (Kumu Inc.), using data from the assembled grids. These were then merged into a single overarching CLD. The purpose of combining the individual school maps was to identify recurring factors and causal processes across workshops, and develop a shared representation of the wider system influencing adolescent night-time IED use and sleep across participating settings for use in intervention development, rather than to compare schools or generate site-specific models. Factors and connections were exported to Microsoft Excel by BR, reviewed, and consolidated (*e.g.,* ‘bad mood’, ‘boredom’, and ‘frustration’ were grouped as ‘emotional dissatisfaction’). Factors were renamed, defined, and categorised using a socioecological model of sleep (*i.e.,* behavioural, educational, environmental, physiological, psychological, social, or technological factors) [59]. Thereafter, a consolidated list of connections was generated by cross-referencing the original lists against the revised factors, combining connections as required. Factors and connections were refined through team discussions, with decisions made about which to retain (*e.g.,* all IED uses collapsed into ‘use of device at night-time’ to reflect the intervention target). Interpretations, factor consolidation decisions and proposed revisions were reviewed iteratively by the research team to ensure consistency and plausibility prior to construction of the combined CLD. The final combined CLD was manually built in Kumu. Factors and causal connections were derived from participant-generated workshop data and transcript coding. Feedback loops were subsequently identified in the participant-derived data using Kumu’s integrated loop detection tool, and manually reviewed and interpreted by the research team for causal plausibility. This phase concluded with preparation for validation workshops, including planning and resource development (see Figure 1).

### Step 4: CLD validation with adolescents (participatory phase)

Validation of the combined CLD was conducted with a fourth school across two online workshops, facilitated by CS and BR. Schools 1–3 were unavailable for further participation, therefore a fourth school was recruited to provide independent adolescent validation of the consolidated CLD, and broaden the contextual diversity represented in the study. Adolescent validation was undertaken first because adolescents were the primary population of interest in the study. This stage enabled assessment of whether the consolidated CLD reflected young people’s perspectives before seeking additional input from stakeholder groups. In Workshop 1, participants engaged in activities from Step 2 (see above) and discussed unresolved connections between factors. Workshop 2 introduced key feedback loops identified in Step 3 (see above), prompting open discussion on their relevance to participants’ lived experiences. Fieldnotes were recorded and data were analysed using the previously described grid method. BR cross-referenced new insights with the existing system map, updating the CLD to reflect adolescent validation. Further team meetings were held, and submaps were created in preparation for Step 5 to support further analysis.

### Step 5: CLD validation with adults (participatory phase)

A second validation workshop, facilitated by CS and BR, was conducted with parents and school staff. Adult stakeholders contributed perspectives on influences operating at home and within schools, including routines, policies and practices that adolescents may not directly observe or articulate. Incorporating perspectives from different parts of the system broadened understanding of influences across home, school and social environments. Activities were adapted from earlier workshops and began with problem scoping and factor elicitation, structured around four guiding questions focused on influences, school policies, and roles of teachers and parents (see Additional File 3). Participants then reviewed submaps from the adolescent-validated CLD to identify additional factors, particularly those relevant to adult perspectives. To make the review of the CLD feasible within the workshop timeframe, a series of submaps were developed that focused on related groups of factors and feedback processes. This enabled participants to engage with sections of the system in sufficient detail, while retaining the broader structure of the overall CLD. They then discussed whether researcher interpretations of the map aligned with their experiences. Fieldnotes were taken to complement the session recording.

Post-workshop, BR analysed the transcript and notes using the previously described processes. The CLD was then updated in Kumu in consultation with CS. Additional feedback loops were identified in two ways: (1) manual inspection of new factors for loop formation; (2) using emerging network analysis techniques to produce submaps with a manageable number of feedback loops operating through potentially key factors [60]. Given the size and complexity of the validated CLD, additional analytical techniques were required to identify potentially influential areas of the system and generate submaps of manageable size for further analysis. Kumu was used to identify the 10 most central factors based on eigenvector, closeness, betweenness, and degree centrality – measures that reflect a factor’s influence, proximity, bridging role, and connectivity within the system [61]. As there are no absolute cut-off scores for centrality measures, we followed common reporting practices in systems mapping studies [60,62] and pragmatically selected ten factors to balance the need for interpretability with retaining sufficient systems complexity for meaningful analysis. These factors were consolidated and used to generate a submap. Thereafter, Kumu’s ‘detect loops’ function identified feedback loops, which were manually reviewed for causal plausibility. This process finalised the participant-informed CLD representing the system affecting adolescent night-time IED use and sleep.

### Step 6: Further analyses – finalising the CLD (desk-based phase)

Recognising that complex problems are viewed through multiple lenses [63], we applied a scientific lens in the third desk-based phase to refine the participant-validated CLD, assess its coherence against existing evidence and further establish rigour. Throughout the study, multiple researchers contributed to data interpretation, map refinement and analysis. AM and XW independently reviewed factor labels, definitions, and connections to remove redundancy, ensure logical coherence, and align the CLD with existing literature. Queries and proposed revisions were discussed in two meetings with BR and resolved through consensus. AM then revised the CLD to incorporate mechanisms that underpinned the CLOCK OFF intervention, which incorporated components of the *ASSIST*, *Sleepy Teens* and *Sound Sleep* programmes. In most cases, these mechanisms were represented by factors already identified during Steps 2–5, and the focus was therefore primarily on cross-referencing CLD factors against the theorised mechanisms of change underpinning CLOCK OFF. A small number of additional factors were introduced where necessary to represent key intervention processes.

HS and HW conducted final checks. The final CLD, created in Kumu, was colour-coded to show alignment with the underpinning programmes. Analysis focused on identifying intervention-relevant feedback processes and mechanisms operating across the system to inform programme theory development. Key feedback loops were identified via four steps: (1) using Kumu’s ‘detect loops’ function to generate a comprehensive list; (2) identifying loops from Steps 4 and 5 that included intervention mechanism factors (i.e., factors representing theorised processes through which CLOCK OFF activities were expected to influence behaviour and outcomes); (3) manually identifying loops that included factors added at Step 6; and (4) identifying additional loops influenced by CLOCK OFF inputs, irrespective of mechanism-related factors. This process led to the identification of two subsystems representing CLOCK OFF mechanisms and key feedback loops, forming the basis of a systems-based programme theory to reduce adolescents’ night-time IED use.

### Step 7: Creating a systems-based programme theory (desk-based phase)

The final desk-based phase traced a programme theory from the CLOCK OFF intervention to outcomes using the causal loop diagram (CLD), providing a whole-system view of both the intervention and the context in which it will be implemented [18]. Classification criteria were informed by systems-based theory of change approaches [18,19] and conventional programme theory frameworks. Factors were categorised according to whether they represented intervention resources (inputs), intervention activities, contextual conditions or mechanisms, or anticipated outcomes within the proposed change processes. Using these criteria, AM recoded CLD factors into established programme theory components: ‘inputs’, ‘activities’, ‘context/assumptions/mechanisms’ and ‘short-, intermediate-, and medium-term outcomes’ (excluding long-term outcomes, which were considered too distal to be mapped). Additional CLOCK OFF-related inputs and activities were added (see Additional File 4), and causal connections were drawn between these and existing factors. All elements were labelled and colour-coded by programme theory category, with ‘context/assumptions/mechanisms’ grouped due to conceptual overlap. AM and BR reviewed the diagram for causal plausibility using three rules. Classification decisions were discussed and refined iteratively during programme theory development:

1. Inputs (*e.g.,* ‘peer nomination survey’) may be influenced by context/assumption/mechanism factors (*e.g.,* ‘connectedness of pupils’) but must connect outwardly to activities (*e.g.,* ‘lessons on sleep’) or short-term outcomes (*e.g.,* ‘parent/guardian knowledge’). Inputs cannot be directly connected to other inputs; their relevance emerges through intermediary activities.
2. Activities (*e.g.,* ‘ongoing support for peer supporter’) must connect inwardly to inputs or context (*e.g.,* ‘trainers have trust and rapport with peer supporters’) and outwardly to short-term (*e.g.,* ‘teacher knowledge’) or intermediate outcomes (*e.g.,* ‘use of device at bedtime’). Activities may also receive connections from outcomes if preceded by another activity (*e.g.,* ‘training of teachers’ → ‘teacher knowledge’ → ‘advice from trusted adults’).
3. Short-term outcomes, or chains of shortterm outcomes (*e.g.,* ‘worry’ → ‘desire to disconnect from content’), must connect to intermediate (*e.g.,* ‘use of device at bedtime’) or medium-term outcomes (*e.g.,* ‘sleep quality/duration’), either directly or via an activity or context/assumption/mechanism factor.

Feedback loops (≤10 connections) were automatically detected via Kumu and manually reviewed for salience and plausibility. Per published recommendations [15], emphasis was placed on clarifying assumptions within key loops linking inputs or activities to expected outcomes, or between different outcomes. The final programme theory diagram illustrated connections and feedback loops across all CLOCK OFF programme theory components, retaining adolescent-derived terminology wherever possible (*e.g.,* factor names). The programme theory was intended to make explicit how a discrete school-based intervention might act upon identified entry points within the wider system, and how these changes could influence broader patterns of adolescent night-time IED use and sleep.

## Results

Following provision of participant characteristics, the results are presented in two parts aligned with the study’s aims. The first section outlines outputs from the CLD building and validation process, summarising how successive map iterations identified key influences on adolescent night-time IED use and sleep. This is followed by a detailed exposition of the systems-based programme theory, including the principal subsystems and feedback processes through which CLOCK OFF is theorised to influence behaviour and sleep outcomes.

### Participants

A total of 23 adolescents (17 girls aged 11–14 years; 6 boys all aged 13 years) and seven adults (5 female; 2 male) took part in the systems mapping components of the study. Table 2 provides a breakdown of participant numbers at each workshop.

**Table 2:** Participant attendance at system mapping workshops (n = 30).

Workshop	School 1	School 2	School 3	School 4	Parents and teachers
CLD diagram building	5	5	8	–	–
First CLD validation	–	–	–	5	7
Second CLD validation	–	–	–	5	–

### Causal loop diagrams

During this study, six system map outputs were co-created. Three initial maps created with adolescents were amalgamated, while the remaining three represented distinct iterations of the combined CLD: one validated by adolescents, one by adults, and a final version with additional researcher input. In this manuscript, these latter iterations are referred to as outputs 1, 2 and 3, respectively. This section primarily focuses on the final CLD, examining some of the interplay between participatory and desk-based research phases.

### Summary of outputs 1 and 2

The adolescent-validated CLD (output 1 – weblink to the systems map can be found here) maps 48 interconnected factors influencing adolescent night-time IED use and sleep. These factors were categorised as behavioural (n = 17), educational (n = 4), environmental (n = 1), physiological (n = 5), psychological (n = 19), social (n = 9) and technological (n = 8). There were 94 connections in the CLD, and 382 mathematically identifiable feedback loops. Further details on output 1 are provided in Additional File 5, which includes detailed examination of six salient feedback loops.

The adult-validated CLD (output 2 – weblink to the systems map can be found here) expanded the system to 55 factors and 107 connections. Seven new factors were added, including ‘addictive qualities of content’, ‘offline schoolwork’, and ‘school restrictions on device use’. Two adolescent-derived connections were removed due to lack of explanatory support. Sixteen new connections were introduced, reflecting adult perspectives on educational and home environments. The full adult map revealed 1,003 mathematically identifiable feedback loops, with a submap identifying 28 key loops (13 balancing, 15 reinforcing). Further details about output 2 are also included in Additional File 5.

Collectively, Outputs 1 and 2 demonstrated how the representation of the system evolved as additional stakeholder perspectives were incorporated. Adolescent mapping primarily highlighted psychological, behavioural and peer-related influences on bedtime IED use, whereas adult validation introduced additional educational and home-environment influences, including schoolwork, school restrictions and parental regulation strategies. These additions broadened understanding of the wider system affecting adolescent IED use and sleep, and informed refinement of the final CLD.

### Output 3: Presentation of the final CLOCK OFF CLD

The weblink to the final CLOCK OFF CLD can be found here. Having incorporated key mechanisms of change underpinning the CLOCK OFF intervention into the map (see green factors), the final version comprised 68 factors and 131 connections, spanning behavioural (n = 22), educational (n = 13), environmental (n = 3), physiological (n = 6), psychological (n = 25), social (n = 10) and technological (n = 9) domains. This process elicited the removal of four factors due to their limited perceived relevance or lack of clear causal influence within the validated CLD (‘awareness of time spent on device’; ‘free time’; ‘perceptions of control’; ‘potential to lose games’) and the addition of 16 new factors. Added factors included educational (*e.g.,* ‘opportunities for sharing curriculum content’), behavioural (*e.g.,* ‘modelling of device use behaviour’) and outcomes-related (*e.g.,* ‘desire to disconnect’; ‘parent/guardian knowledge’) elements. Table 3 describes the 25 factors linked to CLOCK OFF’s mechanisms of change. Readers seeking additional detail regarding the intervention components and approaches that informed CLOCK OFF are directed to Additional File 2. The remaining factors reflect important background conditions and influences within the broader system that shape how and where the intervention will take effect. Additional File 6 provides definitions for all factors in the final CLD.

**Table 3:** Factors representing mechanisms of the CLOCK OFF intervention.

Factors representing mechanisms of change in CLOCK OFF	Definition
Advice from trusted adults	Young people receive advice about using interactive electronic device from adults whom they trust (*e.g.,* parents, other family members, coaches, teachers *etc.*)
Agency	Young people’s ability to control their own behaviours and reactions to circumstances beyond their control, even if their actions are limited by someone or something else.
Awareness of effect of device use at bedtime	Knowledge of the detrimental effects of using interactive electronic devices around bedtime, including the impact on sleep.
Comparison of self with peers	Young people make comparisons with their peers’ behaviours, activities, opportunities *etc.* through interactive electronic devices.
Curriculum-based content	The totality of what is to be taught in a school system, often mandated through statutes and other policies.
Desire to disconnect from content	The intention of young people to actively disengage from or stop interacting with digital or electronic content.
Education from parents/guardians	Developmentally appropriate information provided to, or facilitated with, young people by people in the role of parents/guardians.
Exposure to educational content	Being presented with materials or media that are designed to inform, instruct, or provide knowledge to young people (*e.g.,* educational apps, tutorial videos, websites).
Feelings of being in the loop	Young people feel they have information, or are part of decision-making processes, relating to particular social groups.
FOMO	Fear of missing out – anxiety that an exciting or interesting event may currently be happening elsewhere (without you), often aroused by posts seen on social media.
Good parent-child relationships	A secure attachment between parents/guardians and their children that helps promote a child’s cognitive, emotional and social development.
Modelling of device use behaviour	Young people observe others (any people) using (or not) their interactive electronic devices and then imitate that behaviour.
Offline schoolwork	Giving young people homework that does not involve the need to use interactive electronic devices.
Opportunities for sharing curriculum content	Situations in which educational materials, resources, or information related to sleep can be disseminated or communicated to young people.
Parent/guardian knowledge	Awareness of the effects of interactive electronic device use on sleep among parents/guardians.
Parental/guardian controls	Strategies employed by parents/guardians to limit the amount or type of interactive electronic device use among young people through non-technological means (*e.g.,* time limits, device curfews *etc.*).
Parental/guardian engagement with curriculum content	Parents actively interact with the educational resources designed to reduce electronic device usage at bedtime among their children.
Positive offline social interactions	Positive face-to-face interactions with other people.
Prioritising sleep over other activities	Ability to concentrate on trying to (prepare for) sleep.
Repeat interactive electronic device use	Repeatedly using interactive electronic devices in similar circumstances; may or may not lead to habituation.
Seeking peer contact	The behaviour of young people actively trying to engage with or interact with peers (e.g., texting, social media messaging, online gaming, or any other form of communication or interaction with friends).
Sleep promoting bedtime routines	A series of activities that happen each day before bed, typically carried out in the same order, and often for the purpose of producing a calming effect.
Teacher knowledge	Awareness among teachers of the effects of interactive electronic device use on sleep.
Technological controls	Strategies employed by parents/guardians to limit the amount or type of interactive electronic device use among young people through technological means (*e.g.,* WiFi blockers or content controls *etc.*).
Worry	The prime cognitive characteristic of worry, comprising a chain of thoughts and images, which are negatively affect-laden and relatively uncontrollable.

In step 6 of the map-building process, 14 connections were removed to streamline overlapping factors, improve causal clarity, and align with existing research. This was followed by the addition of 38 new connections: two linking new factors together, and the rest connecting new factors to existing ones. All updates are detailed in Additional File 7. The final CLOCK OFF CLD included 1,431 mathematically identifiable feedback loops detected by Kumu, reflecting the complexity and interconnectedness of the system. Many of these represented variations of broader recurring feedback structures that shared common factors and causal pathways. Initial analyses therefore examined the distribution of intervention-related factors across the full set of loops, while subsequent interpretative analyses focused on theoretically salient loops that were causally plausible and relevant to CLOCK OFF mechanisms, or informative for programme theory development.

Including CLOCK OFF’s mechanisms of change enabled examination of how the intervention may influence adolescent IED use and sleep behaviours. Of the 1,431 mathematically identifiable feedback loops detected, 1,358 (94.9%) contained at least one factor directly related to a CLOCK OFF mechanism. These factors were present in 82.8% of loops with up to 9 connections, and in all loops with 10 or more, highlighting CLOCK OFF’s potential to influence a broad range of interconnected feedback processes across the system. This preliminary examination also emphasised the importance of raising awareness of the reinforcing effects of bedtime IED use (*e.g.,* ‘exposure to distressing/emotion-triggering content’ and ‘emotional dissatisfaction’) and supporting aims to reduce these.

Notably, loops downstream of ‘use of device at bedtime’ that contained the effects of IED use were most commonly missing intervention-related factors. Factors related to intervention mechanisms were present in 12 of the 15 previously identified balancing feedback loops (B1-B3; B5; B8-15), and four of 17 reinforcing loops (R1; R3; R7; R17) – see Additional File 8.

More broadly, the final CLD suggested that adolescent night-time IED use was maintained through interacting social, emotional and educational processes rather than any single causal influence. Peer interactions and FOMO shaped motivations for device use, emotional processes linked IED use to sleep-related outcomes, and educational and family influences provided potential mechanisms through which behaviours could be regulated. These analyses also led to the identification of two key subsystems, together containing 14 of the 25 intervention-related factors.

#### Knowledge and awareness (curriculum content) subsystem.

Around a third (9/25) of the CLOCK OFF-related mechanism factors were connected within the ‘knowledge and awareness’ subsystem, which was shaped by the ripple effects of ‘curriculum-based content’. This subsystem, illustrated in Fig 4, functions as an upstream influence within the wider system by increasing awareness of the effects of bedtime IED use among adolescents, teachers, parents/guardians and other trusted adults. However, its influence on IED use is largely indirect, operating through changes in beliefs, conversations and behavioural expectations that may encourage adolescents to self-regulate their device use or support adults to introduce and maintain bedtime IED controls.

**Fig 4:**
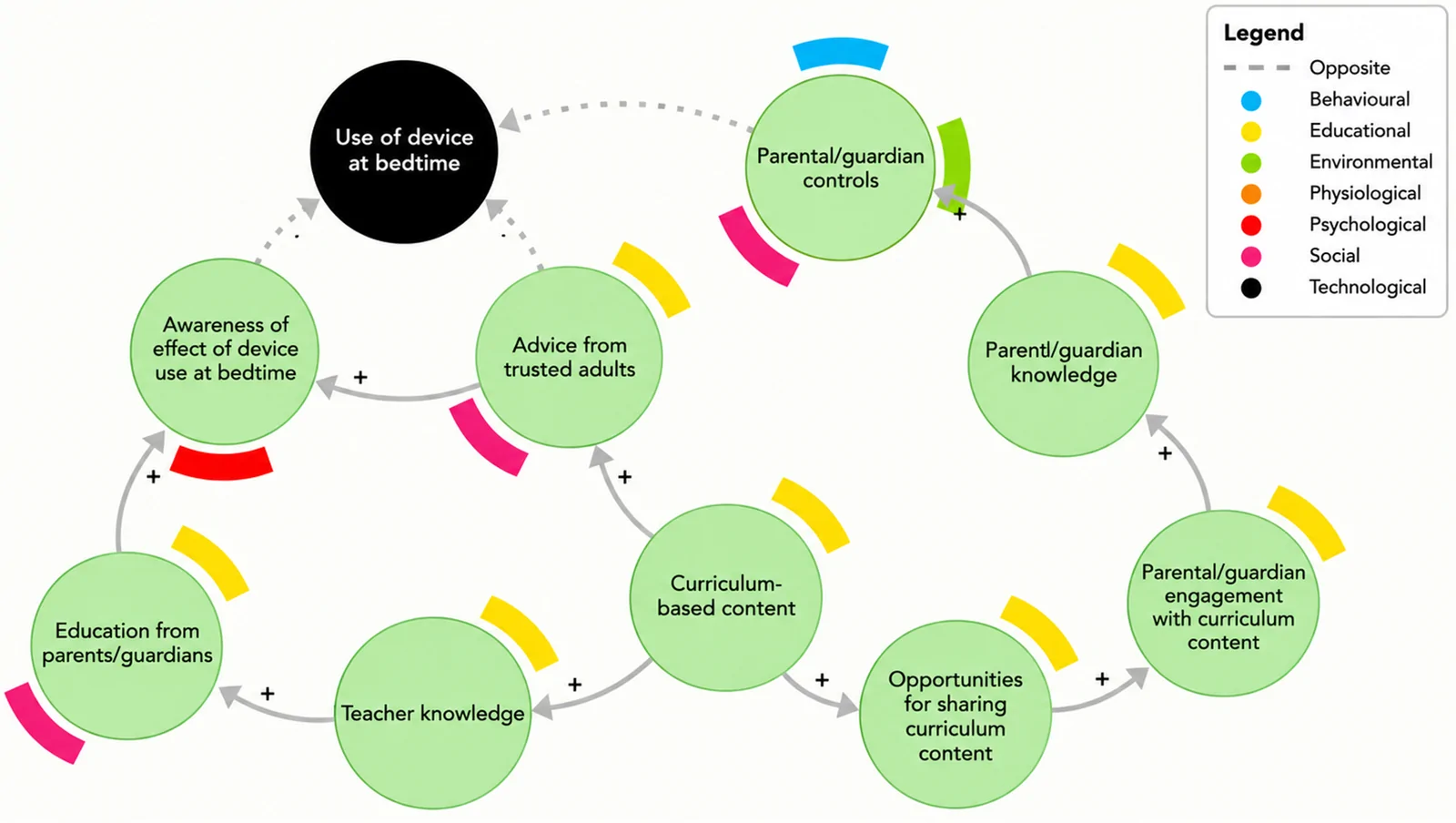
Knowledge and awareness submap (curriculum influence).

As shown in earlier CLD iterations, these controls were integral to several key feedback loops, highlighting that curriculum content alone is unlikely to reduce bedtime IED use unless it translates into changes in behaviour and the home environment. The subsystem therefore illustrates how knowledge acquisition interacts with other parts of the system, particularly parental regulation and school practices, rather than acting as an isolated intervention mechanism.

However, loop R18 highlights a potential, unintended consequence of curriculum-linked interventions. Adolescents frequently described using IEDs at bedtime for educational purposes (*e.g.,* searching online for homework, which adolescents indicated was not uncommon). Although this may increase ‘learning’, it may also simultaneously extend to an ‘overload of information’, ‘stress’ and ‘emotional dissatisfaction’. These factors may reinforce further IED use, while also contributing to poorer sleep outcomes linked to ‘emotional dissatisfaction’. This tension suggests that interventions promoting digital learning should be accompanied by guidance on when and how technology is used, as increasing knowledge alone may inadvertently strengthen behaviours that undermine an intervention’s intended effects.

#### Peer interactions and worry subsystem.

The second key subsystem (Fig 5) centres on ‘worry’ as a key mechanism linking adolescents’ bedtime IED use with their online social experiences. Unlike the knowledge and awareness subsystem, this subsystem contains both balancing and reinforcing feedback processes, suggesting that ‘worry’ can either reduce or sustain bedtime IED use depending on its underlying cause and adolescents’ behavioural response.

**Fig 5:**
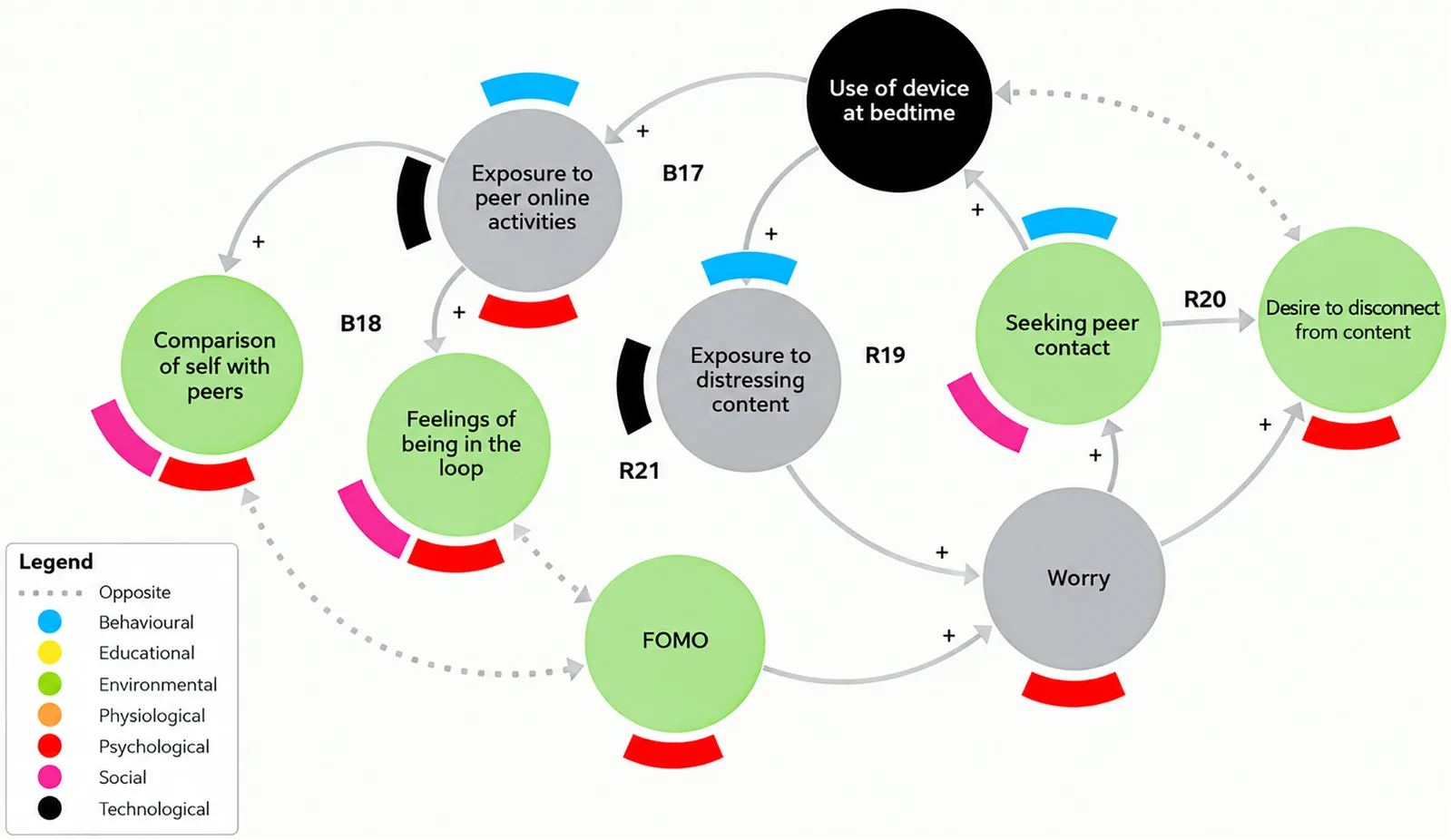
Peer interactions and worry submap.

The balancing loops illustrate how increased ‘worry’ may ultimately reduce IED use. Greater ‘exposure to distressing content’ online may increase ‘worry,’ which in turn increases the ‘desire to disconnect from content’ (B16). Similarly, ‘exposure to peer online activities’ may reduce ‘FOMO’ by increasing ‘feelings of being in the loop’ or facilitating ‘comparison of self with peers,’ reducing the need for ‘seeking peer contact’ and subsequent bedtime IED use (B17-B18). Together, these balancing processes suggest that online engagement may temporarily satisfy adolescents’ social needs or encourage disengagement when online experiences become emotionally overwhelming.

In contrast, reinforcing loops (R19-R21) demonstrate how ‘worry’ may perpetuate ‘use of device at bedtime.’ ‘Exposure to distressing content’ can increase ‘worry,’ prompting greater ‘seeking peer contact’ rather than a ‘desire to disconnect from content,’ thereby increasing bedtime IED use and creating further opportunities for exposure to distressing online experiences.

The coexistence of these balancing and reinforcing processes identifies ‘worry’ as a decision point within the system, where adolescents may either disengage from, or further engage with, online content. This suggests that the impact of ‘worry’ on bedtime IED use depends less on the emotion itself than on how adolescents respond to it. Consequently, interventions that reduce ‘FOMO,’ strengthen offline social support and promote adaptive responses to online distress may be more effective than strategies that focus solely on limiting device access.

### Output 4: Systems-based programme theory

The weblink to the system-based programme theory can be found here. It comprises 72 factors, 138 connections and 147 automatically detected feedback loops, illustrating the complex, non-linear processes underpinning CLOCK OFF. Factors include 5 inputs, 23 activities, 28 context/assumption/mechanism factors, 14 short-term outcomes, 1 intermediate outcome (‘use of device at bedtime’) and 1 medium-term outcome (‘sleep quality/duration’).

Analysis of the feedback loops identified 25 salient loops (14 reinforcing and 11 balancing), which spanned assumptions between activities and outcomes (n = 1), and between outcome types (n = 24). The loops were grouped into five subsystems, each reflecting key aspects of the intervention’s logic in influencing adolescent IED use and sleep outcomes. Unlike those presented in output 3, which describes structures within the final CLD, these subsystems represent a programme theory-level analysis of how CLOCK OFF is theorised to influence the wider system. Collectively, they illustrate the principal pathways through which intervention activities are expected to interact within existing system dynamics to influence adolescent IED use and sleep. These subsystem groupings are complementary rather than equivalent. Factor types and feedback loops are described fully in Additional File 9.

### Emotions and enjoyment

Fig 6 illustrates a key assumption in the programme theory: that adolescents derive a degree of ‘enjoyment’ from using their IEDs at bedtime; ‘enjoyment’ drives ‘prolonged device use’, which in turn triggers a chain of causal effects culminating in a delicate balancing response (B19).

**Fig 6:**
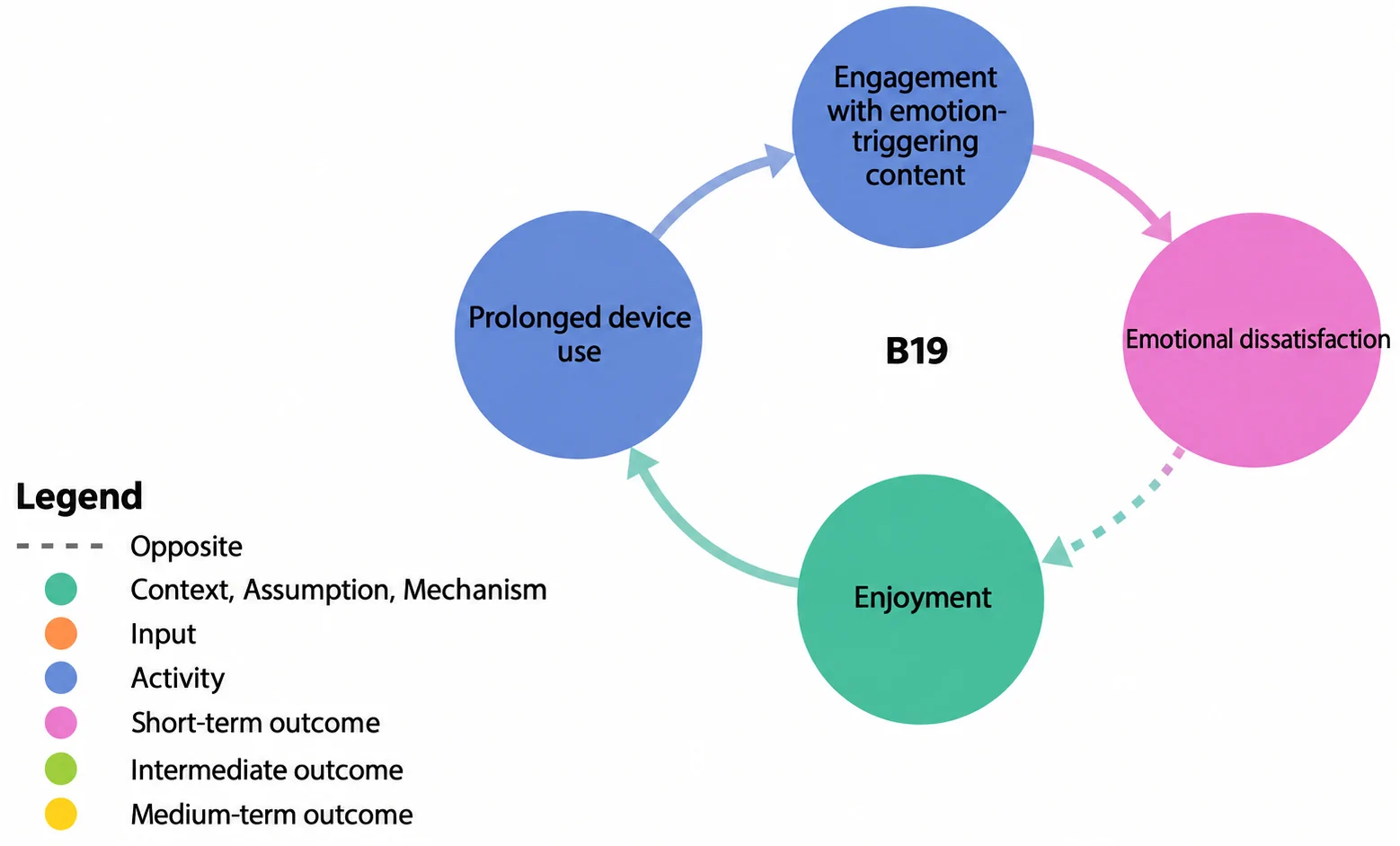
Emotions and enjoyment submap.

As ‘enjoyment’ of using IEDs increases, so too does ‘prolonged device use’, ‘engagement with emotion-triggering content’ (a key activity) and ‘emotional dissatisfaction’. This increase in ‘emotional dissatisfaction’ has a dampening effect on ‘enjoyment’, which in turn reduces ‘prolonged device use’. Over time, this cycle may repeat, leading to oscillating effects on ‘enjoyment’ levels and ‘prolonged device use’, meaning little net change in IED use among adolescents. Our programme theory demonstrates how CLOCK OFF has the potential to disrupt this pattern of events, through ‘cognitive restructuring’ (an activity acting on ‘emotional dissatisfaction’) and ‘modelling of desired IED use behaviour’ (an activity effecting change in ‘engagement with emotion-triggering content’). ‘Cognitive restructuring’ may be used to help adolescents reinterpret their emotions and perceived ‘enjoyment’ from IED use, while modelling desired behaviours can be reinforcing at times when levels of ‘prolonged device use’ are lower.

### Overstimulation

Fig 7 illustrates the overstimulation submap, which describes two balancing feedback loops that can dampen the initial impacts of increased IED use. Linked to the previous theme of *emotions and enjoyment*, this submap explains how ‘prolonged device use’ leads to an increase in emotional and/or cognitive ‘overstimulation’ among adolescents. This overstimulation can lead to one of two effects. First, an increase in ‘parental/guardian controls’, which leads to a decrease in IED use (B20). Alternatively, it is assumed that adolescents are able to recognise when they are becoming overstimulated, which in turn heightens adolescents’ ‘fear of being caught’ using IEDs at times they should not be by parents/guardians. Therefore they self-regulate their behaviour to avoid actually being caught, and reduce their IED use (B21). Both causal pathways informed the inclusion of content relating to ‘overstimulation’ and effective measures in response to this (either parent/guardian or adolescent led), within CLOCK OFF.

**Fig 7:**
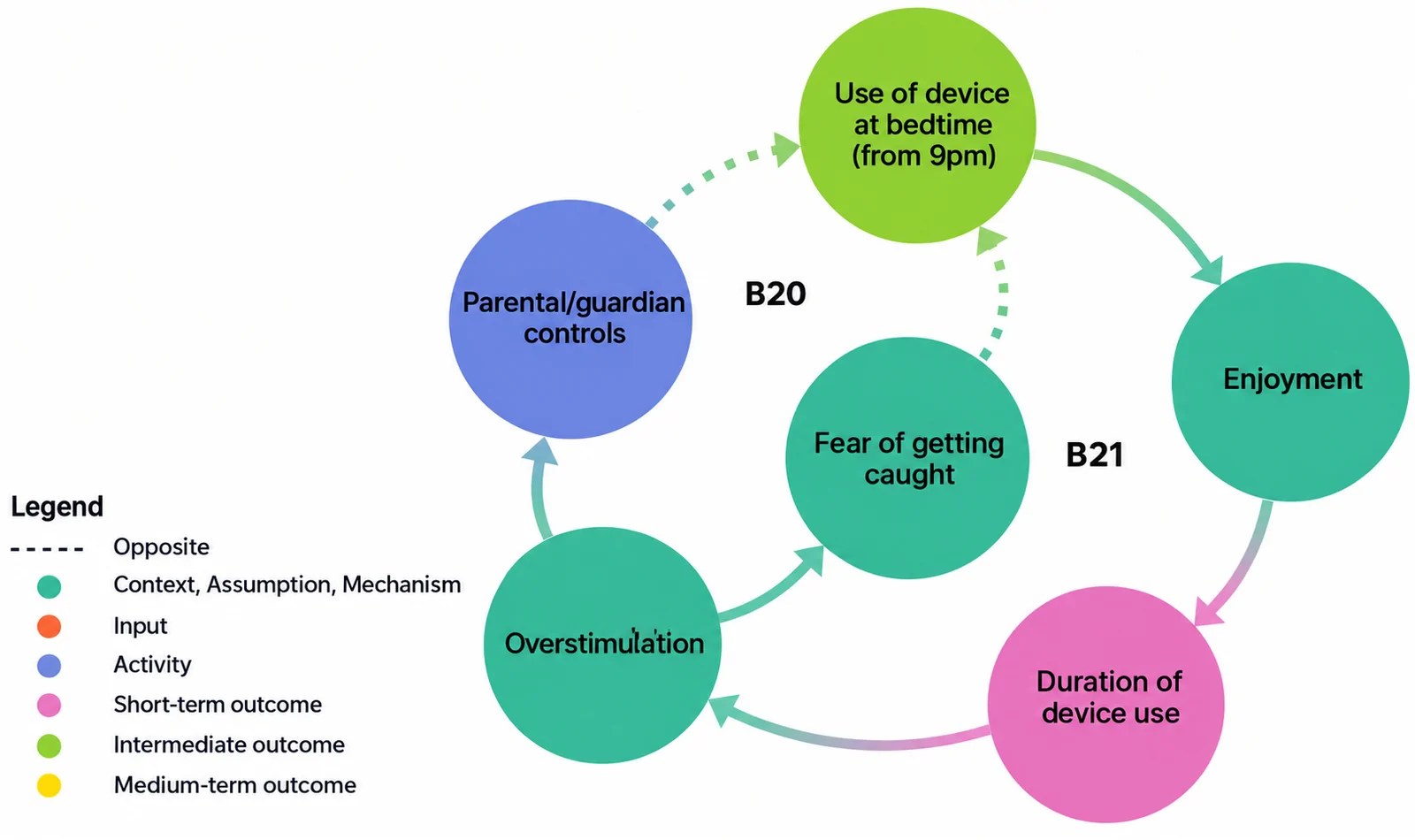
Overstimulation submap.

### Fear of missing out

FOMO was an integral factor in 13 feedback loops (B23-B31; R31-R34), which illustrates the importance of understanding and addressing the complex array of causal influences on this psychological outcome through CLOCK OFF. These loops describe the multiple and conjunctural pathways through which the effects of IED use and FOMO can be either balancing or reinforcing. Fig 8 depicts the *FOMO* subsystem in our programme theory diagram.

**Fig 8:**
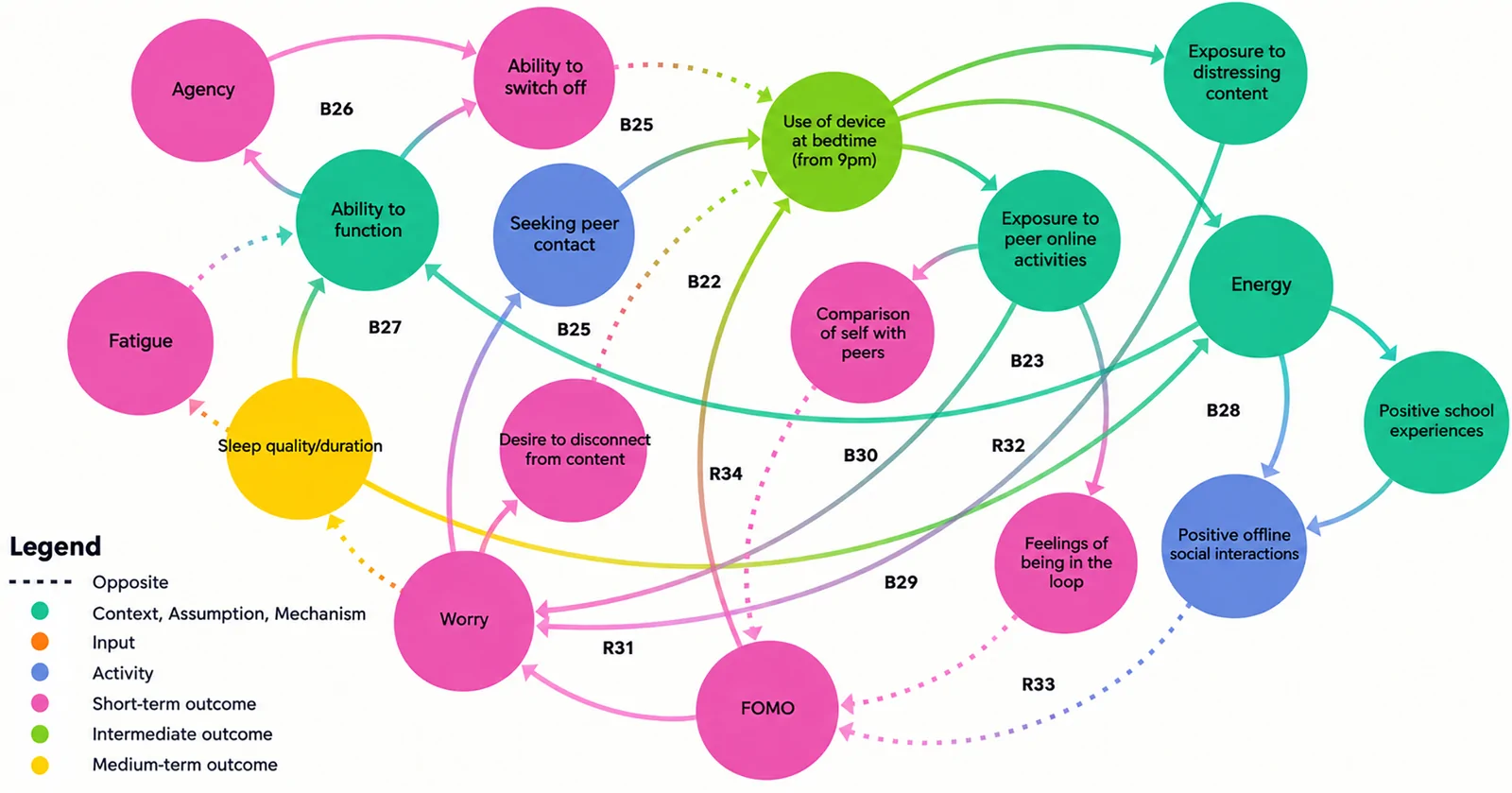
FOMO submap.

With the exception of loop B29 (concerning how IED use lowers ‘energy’ for the activity ‘positive offline social interactions’, leading to ‘FOMO’), the remaining balancing loops were driven by ‘exposure to peer online activities’. As this exposure increases, adolescents’ ‘feelings of being in the loop’ and ‘comparison of self with peers’ also increases. These factors both have the effect of reducing ‘FOMO’. This reduction in ‘FOMO’ reduces ‘worry’ among adolescents and leads to a chain of effects driven by the activity ‘seeking peer contact’, and a second via the medium-term outcome of increased ‘sleep quality/duration’. In this context, adolescents who ‘worry’ less have reduced need for ‘seeking peer contact’ and therefore use their IEDs less at bedtime. Meanwhile, the improved sleep outcomes caused by reductions in ‘worry’, increase ‘ability to function’, adolescents’ ‘agency’ and ‘ability to switch off’. This sequence of factors results in a reduction in ‘use of device at bedtime’. Underpinning these loops is the assumption that IED use is an important mechanism by which adolescents manage their sense of FOMO, and this needs to be considered in the implementation of CLOCK OFF. The intervention incorporates activities to build ‘positive offline social interactions’ (key components of reinforcing loops R32-R34, which are related to positive school experiences), and strategies for managing helpful IED use at more optimal times of day.

### Ability to function and regulate device use

Fig 9 illustrates the *ability to function and regulate device use* subsystem. It comprises eight related reinforcing feedback loops. Each shared four factors linked in a specific order: a decreased ‘ability to function’ is related to consequent decreases in ‘agency’ and ‘ability to switch off’, which in turn increased ‘use of device at bedtime’. The initial change in ‘ability to function’ is the product of several causal pathways: a decrease in ‘sleep quality/duration’ and/or ‘energy’ caused by IED use; or ‘exposure to distressing content’ resulting from ‘use of device at bedtime’, which causes ‘worry’, decreased ‘sleep quality/duration’ and ‘fatigue’. ‘Positive offline social interactions’ may also decrease ‘FOMO’ and ‘worry’, though these effects may be dampened by a lack of ‘energy’ resulting from ‘use of device at bedtime’.

**Fig 9:**
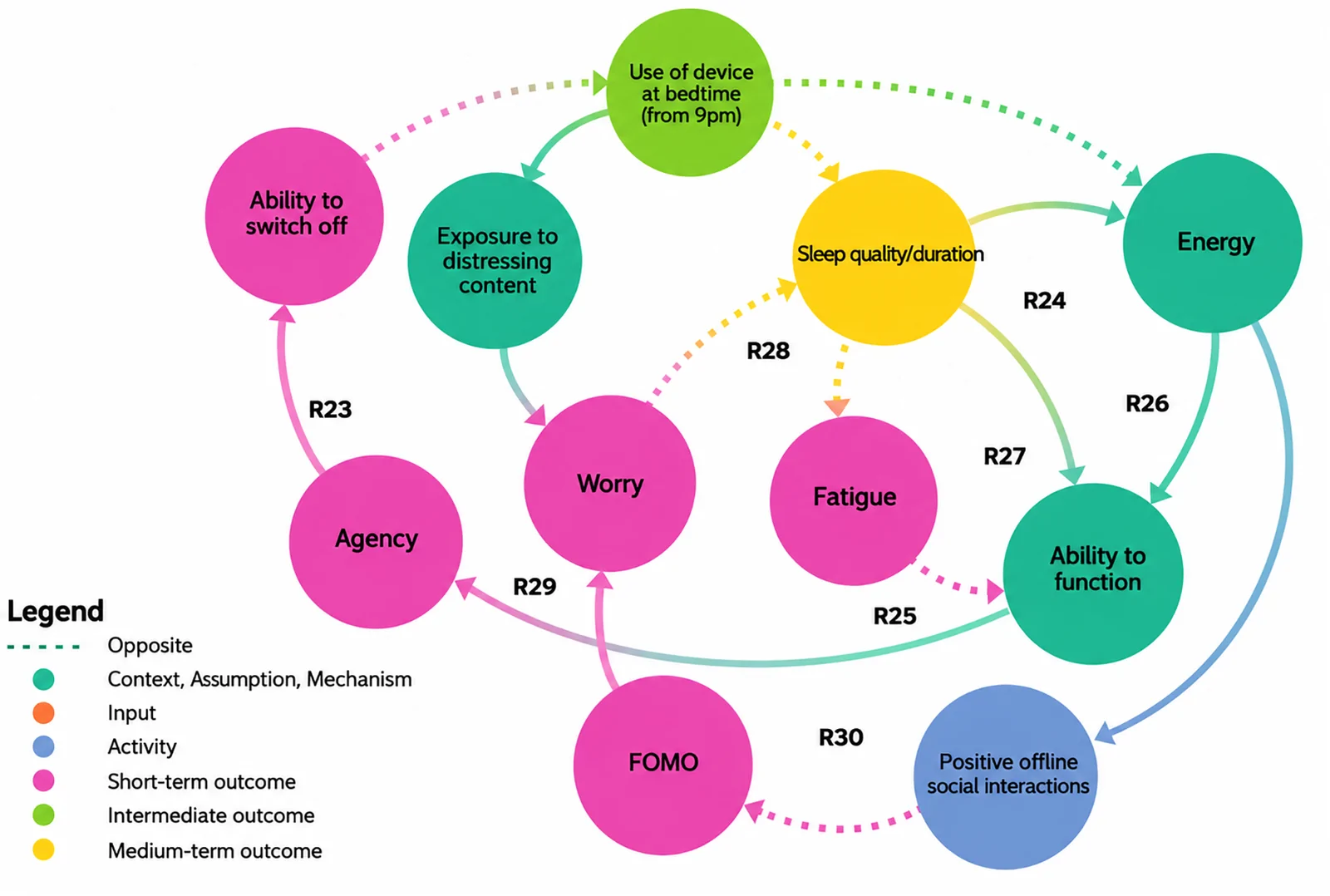
Ability to function and regulate device use submap.

### Stressful education

The programme theory provides a further cautionary note about the potential unintended consequences of implementing curriculum-based content in CLOCK OFF. An increased ‘use of device at bedtime’ can increase ‘exposure to educational content’ (complemented by activities such as ‘diffusing sleep promoting messages’). While this can increase in ‘learning’, it may also lead to an ‘overload of information’ and increased ‘stress’. This in turn, may heighten the ‘desire to wind down’ and encourage further ‘use of device at bedtime’ to alleviate this education-induced ‘stress’ before sleep. This impresses the importance of messaging that both raises awareness of the challenges associated with the use of IEDs for educational purposes near bedtime, as well promotes alternative inputs and activities that do not rely on such devices.

## Discussion

To our knowledge, this study presents the first public health-focused systems-based programme theory that adapts key structures of traditional linear models (inputs, activities, context/assumption/mechanisms, short-term outcomes, intermediate outcomes and medium-term outcomes). Guided by MRC recommendations [12], we adopted a systems perspective to ensure the development of an intervention that was theoretically-informed, responded to the context causes of the problem and was contextually sensitive. Our primary aim was to conduct participatory systems mapping with adolescents, parents and teachers to develop a programme theory for CLOCK OFF, a peer-influence intervention targeting adolescents’ night-time IED use and improving sleep. We also sought to generate methodological reflections on the use of these methods with adolescents. The outputs, comprising CLDs and programme theory, were developed through rigorous participant- and researcher-led processes. These CLDs illustrate the digital environment subsystem, its likely influence on IED use and sleep, and the causal logic and contextual conditions underpinning CLOCK OFF, as defined by adolescents, parents/guardians and teachers. While the specific programme theory developed here is unique to CLOCK OFF and its implementation context, the approach demonstrates how participatory systems mapping can be used to explicate mechanisms, contextual influences and feedback processes that may inform the development of other sleep- and health-related interventions.

The participatory systems mapping process also directly informed development of the CLOCK OFF programme theory. For example, adolescents consistently highlighted the importance of positive offline social interactions, supporting inclusion of activities intended to strengthen social connection beyond digital environments. Similarly, adolescents frequently described worry arising from online social interactions, reinforcing emphasis on mechanisms relating to FOMO, peer contact and emotional regulation. Further, participants also highlighted practical strategies for circumventing parental controls and technological restrictions, emphasising the need to support self-regulation through CLOCK OFF, rather than merely relying on external control measures.

### Findings related to intervention content

Our findings support existing research on the multiple and multi-level factors that influence adolescent sleep health [3,4,6,7,9]. Past studies have demonstrated how individual- (*e.g.,* beliefs, affect, attitudes), social- (*e.g.,* family environment) and societal-level (*e.g.,* technology) factors interweave to influence sleep. We adopted a socioecological model of sleep [59] to categorise CLD factors and found multiple influences across behavioural (n = 22), educational (n = 13), environmental (n = 3), physiological (n = 6), psychological (n = 25), social (n = 10) and technological (n = 9) domains. However, by conducting participatory systems mapping we contributed to a nascent body of research that extends socioecological perspectives [9], by making explicit mental models about how IED use- and sleep-influencing factors interact in complex causal dynamics. We augment this emerging evidence by providing a deep-dive into the causal processes of the digital environment [9] and surrounding subsystems, with a particular emphasis on the social environment around IED use. For example, while our findings support previously observed subsystems related to how IED use postpones bedtime/sleep, and how FOMO is an important driver of IED use [9], we have been able to show how, within the context of CLOCK OFF, reductions in FOMO can be effected and leveraged to decrease device use through several pathways.

These findings extend those of Heemskerk et al. [9], who identified the digital environment as one of six interacting subsystems influencing adolescent sleep health and used the action scales model to identify opportunities for intervention. While their work provided important insight into the structure of the sleep system, our study builds on this by examining how key feedback processes operating within the digital environment and related social subsystems may generate change. In particular, the systems-based programme theory makes explicit how intervention activities are theorised to interact with mechanisms such as social connectedness, FOMO, worry and self-regulation to influence adolescent IED use and sleep outcomes.

A key contribution of the CLD and systems-based programme theory was the identification of interacting social feedback processes linking adolescents’ need for social connectedness with bedtime IED use. Across both the *peer interactions and worry* (output 3) and *FOMO* (output 4) subsystems, factor’s such as ‘feelings of being in the loop’, ‘comparison of self with peers’, ‘seeking peer contact’, ‘worry’ and ‘FOMO’ formed interconnected feedback structures that could either reinforce or dampen bedtime IED use. Previous research indicated the role of parents in regulating IED use [64], and the importance of balanced approach to IED use [65] to help prevent FOMO. Our findings addresses calls for a better understanding of the factors interacting with FOMO [66], and extend evidence by showing how factors such as device ‘notifications’, ‘positive offline social interactions’ and peer comparison may operate within feedback processes that influence changes in both FOMO and subsequent IED use. More broadly, other affective dimensions (*e.g.,* ‘engagement with emotion-triggering content’ and *‘*enjoyment’) also emerged as important components of identified subsystems (*emotions and enjoyment; overstimulation*). However, with the exception of *‘sleep promoting routines’* and *‘worry’*, our participants and resultant maps focused less on key mental wellbeing factors (*e.g.,* finding support, cyberbullying and perfectionism), compared to previous mapping work [9].

The programme theory further identified a reinforcing mechanism linking sleep, social functioning and bedtime IED use. Peer influence [49,51] and ‘positive offline social interactions’ appeared important within this mechanism (although adolescents themselves had mixed feelings about this). However, their effects were dependent on adolescents’ *ability to function and regulate device use*, which in turn was influenced by feelings of ‘energy’ and/or ‘fatigue’. Night-time IED use is associated with daytime tiredness [67], while tiredness is associated with poorer social outcomes [68]. Our maps extend these observations by illustrating the feedback processes through which poorer sleep may undermine social functioning and self-regulation, thereby increasing vulnerability to further bedtime IED use.

Finally, it has been suggested that adolescents may benefit from education about sleep health and the detrimental impact of night-time IED use [69]. We argue that this should be complemented by initiatives that also enhance knowledge among teachers, parents/guardians and other trusted adults. However, our findings raise a potential concern when adolescents are asked to engage with digital educational content. Both completing homework near bedtime and using devices to enhance schoolwork efficiency are both known contributors of night-time IED use and poor sleep outcomes [9,70]. Outputs 3 and 4 highlight how this may be stressful for adolescents; more research is needed to examine the extent to which the format of educational materials influences behaviour and outcomes.

Although the resultant maps focused on school, family, peer and digital environments, several processes are likely shaped by wider socio-environmental factors, such as platform design, commercial incentives and social norms surrounding connectivity. Combined, our findings point toward the multiple and conjunctural causality that underpins the relation between night-time IED use by adolescents and sleep outcomes, as well as the impact of influences seeking to improve these behaviours. This emphasises the need to consider adopting a whole school approach, which integrates behaviours and practices of key actors (*i.e.,* adolescents, parents/guardians and teachers), with changes in school ethos and curricula, and opportunities to extend learning to the home and community contexts [71]. However, successful implementation depends on meaningful teacher involvement, which is a well-documented challenge shaped by professional autonomy, workload, leadership support and collaborative school cultures, rather than simply resistance to change [72–74]. CLOCK OFF is a step toward this endeavour, operating on a number of interconnected social, educational and self-regulatory processes identified within the system. A whole school approach should be nested within broader initiatives of whole system change, which focus on the macro and socio-environmental conditions that perpetuate IED use (*e.g.,* commercial and connectivity drivers embedded in digital platforms, and policy environments shaping technology use). Interventions such as CLOCK OFF should therefore be viewed as one component of a wider response rather than a complete solution to the problem.

### Reflections on implementing participatory systems mapping with adolescents

Participatory systems mapping with adolescents remains an emerging area of research [9,26,29,35], with relatively little reporting on the implementation of these methods. Our paper contributes to the response to calls for more detailed reporting and reflection on participatory research methods with adolescents [42], as well as participatory and analytical processes in systems mapping more broadly [14,15,35]. Here we offer researcher reflections on implementing participatory systems mapping within the present study. These reflections are intended as practice-based considerations that may help inform future applications of these methods, while recognising that they were not derived from interviews, focus groups or other systematic evaluation of participants’ experiences. They should therefore be interpreted as methodological observations rather than empirical evidence.

We experienced the research process as a dialogue between the different phases of participant- and researcher-led steps, which culminated in a final CLD from which the programme theory map was developed. In a sense, the maps evolved through turn-taking, whereby each step may be considered a response to the previous one, through an active and dynamic exchange between different knowledge systems (*i.e.,* that of the adolescents, adult stakeholders or researchers). This dialogical process was partly driven by practical considerations, including participant availability and time for researchers to analyse and synthesise findings between stages. It also highlighted the important role of researcher interpretation. Although the initial maps were participant-generated, subsequent stages required decisions regarding factor consolidation, causal connections, subsystem identification and programme theory development. The resulting outputs therefore reflected an ongoing dialogue between participant perspectives, researcher interpretation and existing evidence.

Participatory systems mapping requires considerable commitment from participants and is often time and labour intensive [35]. This can be especially challenging for schools (*e.g.,* for timetabling) and adolescents (*e.g.,* developmental and environmental influences on attention). To mitigate this, we implemented rapid mapping activities (step 2), which were restricted to 90-minute workshops. As a research team, we identified minimum data requirements from each workshop, which would enable us to move to the next step (*e.g.,* a list of factors influencing device use, which could be cross-referenced with existing literature to identify causal connections if required). While we echo previous reflections about ideally wanting additional time with participants to refine system maps [35], in our experience the activities implemented were manageable for participants and generated sufficient data from which to further map development. It is important to note, however, that while the activities with adolescents themselves were rapid relative to those in previously published studies, the overall process from step 1–7 remained time consuming. In particular, additional time should be set aside for the desk-based steps (1, 3, 6 and 7). There remains need for the development of analytical processes that can be actioned more quickly.

A further consideration in using participatory systems mapping with adolescents relates to the perceived difficulty of applying systems methods among non-methodological experts [45]. While experienced system mappers helped facilitate the sessions (also a time-saving measure [15]), our impression as facilitators was that adolescents appeared able to conceptualise causes and effects, and perhaps aided by familiarity with the technological tools used, generally found the mapping process intuitive (note, researchers often had to infer and review directionality of connections). Given that children as young as four years are capable of causal reasoning [42], that effective systems mapping does not depend on participants being introduced to systems-thinking concepts [75], and that there is an emerging body of work demonstrating adolescent participation in systems mapping [9,26,29,35], we encourage future research to consider this approach as a viable and valuable option.

A final reflection from this study relates to the type of data generated through adolescent workshops. While a systems approach helps extend socioecological models by focusing on interactions across multiple levels [9], our observations suggested that adolescents typically discussed more individual psychological and behavioural level factors that influenced their use of IEDs, alongside some social influences. The reasons for this emphasis cannot be determined from the present study because we did not explore participants’ experiences of the mapping process. It is possible that adolescents naturally prioritised influences most immediately salient to their own experiences, consistent with theories emphasising self-focus and susceptibility to peer influence during adolescence [76], although this interpretation requires empirical investigation. The resultant micro- and meso-level view of the system generated by adolescents reinforced the importance of speaking to adults whose tendencies, we speculate, may incline toward more environmental and macro-level considerations, as well as conducting additional desk-based activities and reviewing research evidence. This may partly explain why broader social-environmental and macro-level influences were less evident in the participant-generated maps. Future mapping research may consider setting system boundaries [56] that direct adolescent reflection to higher-level system constructs, alongside incorporating additional stakeholder groups such as policymakers, regulators, technology developers and industry representatives, to better capture these wider system dynamics. This may be particularly important with changes in political discourse on interactive electronic device use, such as the recently announced bans on social media for children aged under 16 years in Australia and the UK [77,78].

Participatory systems mapping may also function as an intervention in its own right [15]. Through reflecting on factors influencing night-time IED use and sleep, engaging with others’ perspectives, and exploring the complex interconnections shaping behaviour, participants may develop new insights that influence how they understand and respond to these issues. However, this possibility was not examined in the present study. Our overarching reflection is that participatory systems mapping with adolescents is that it was a feasible approach for eliciting adolescents’ mental models and informing the development of the CLOCK OFF programme theory. The considerations below should be viewed as practical reflections from implementing the approach, rather than evidence-based recommendations:

1. These methods are flexible. It is important to consider the balance between pragmatism and rigour required in a given project
2. Online platforms, such as Sticky Studio, enable adolescents to engage in ways other than verbal discussion (*e.g.,* if they are shy, or peers dominate discussion). They may also help prevent groupthink. Note, software familiarisation will be required
3. Having an adult present with whom they are familiar (*e.g.,* a teacher) can assist with behaviour management, or technology support, during workshops, though may present a barrier to openness
4. Additional workshop facilitators may be necessary to help ensure adolescents stay focused and mapping is conducted efficiently
5. Consider integrating engaging and creative activities (*e.g.,* storytelling), which are deemed helpful principles of participatory research with adolescents

### Limitations

Despite the strengths of our study, including the way the process informed our programme theory targeting specific levers within the system, and offering insights into how CLOCK OFF fits and interacts with the wider context, several limitations warrant mention. First, the outcomes were inherently shaped by the participants. Different participants bring distinct mental models, which would likely result in alternative system representations. While we included participants from different UK regions and conducted multi-actor validation to enhance transferability, this remains one representation of the system among many. In addition, schools identified eligible adolescents, which may have introduced selection bias if pupils perceived as more engaged, interested or digitally literate were preferentially recruited, potentially underrepresenting some perspectives. Future research should investigate how sex and gender shape bedtime IED use, recognising known differences in digital behaviours and social media experiences were aggregated through our study design [79,80]. Furthermore, research should explore perspectives from other groups (*e.g.,* adolescents from diverse backgrounds and policymakers), to more comprehensively depict the system and help elicit more macro-level influences less prominent in the current map (*e.g.,* commercial, regulatory and policy factors that shape adolescents’ digital environment). Additional participatory systems mapping with different groups of adolescents may also further extend understanding of the system and its contextual variation.

Second, while we conducted robust validation with adolescents, practical constraints prevented us from involving the same group in both map-building and validation stages. Ideally, we would have reconvened with the original cohorts to sense-check whether the outputs and our desk-based synthesis (step 3) reflected their perceptions of the system. Owing to practical challenges in re-accessing participating schools, we were also unable to conduct adolescent validation of the adult-informed CLD. Nonetheless, our validation workshop revealed consistent themes across groups. We also acknowledge that due to time constraints, we did not collect evaluation data from participants about the workshop activities, which would have contributed to assessing the mapping process and its methodological value [81]. Future studies should prioritise collecting such data through approaches such as reflection forms, interviews, focus groups or other workshop evaluation activities.

Third, the selection of feedback loops involved was primarily researcher-led, based on data and literature, and guided by the need to synthesise a large volume of information relevant to CLOCK OFF. While little guidance exists on selecting feedback loops, we drew on emerging strategies (*e.g.,* from ToC mapping and network analyses) [19,60]. However, alternative filtering approaches may yield different findings.

Finally, the close alignment between our CLD and the CLOCK OFF intervention may reflect the fact that workshops were not conducted blind to the intervention’s components. Facilitators’ prior knowledge of peer-influence strategies (*i.e.,* those underpinning the programmes adapted in the development of CLOCK OFF) may have influenced the later stages of map refinement and programme theory development by shaping the interpretation and retention of factors aligned with the existing intervention concepts. Although participant-led mapping, stakeholder validation and iterative review of existing evidence were used to mitigate this potential interpretative/confirmation bias, some influence of prior intervention knowledge cannot be ruled out. Future work should consider additional strategies to further reduce this risk.

## Conclusions

This study outlines the development of a systems-based programme theory for CLOCK OFF, a peer-influence intervention aimed at reducing adolescent night-time IED use to improve sleep outcomes. The resultant map illustrates the value of generating dynamic representations of programme theory, which move beyond CLDs, to support complex intervention development. We have critically examined the methodological and practical considerations of involving adolescents in systems mapping, demonstrating its utility in capturing the complex interplay of factors that shape IED use behaviours. Our findings underscore the dynamic nature of subsystem interactions and feedback loops, and how these insights can inform intervention design.

Importantly, while IED use may serve protective functions for adolescents (*e.g.,* fulfilling social needs and alleviating anxiety), there remains a pressing need for nuanced, multi-component strategies to mitigate its adverse effects on sleep. These findings support the case for multi-actor (including peer-influence), multi-level interventions, embedded within broader systemic solutions.

## Supporting information

S1 File S1 overview of CLOCK OFF components. (PDF)

S2 File S2 overview of the ASSIST, Sound Sleep and #Sleepyteens. (PDF)

S3 File S3 workshop guides. (PDF)

S4 File S4 inputs and activities of CLOCK OFF. (PDF)

S5 File S5 - outputs 1 and 2. (PDF)

S6 File S6 CLOCK OFF CLD factor definitions and categories. (PDF)

S7 File S7 changes to CLD factors and connections. (PDF)

S8 File S8 key feedback loops (final CLOCK OFF CLD). (PDF)

S9 File S9 components and feedback loops of programme theory. (PDF)

S10 File S10 – CLOCK OFF systems mapping data. (XLSX)

## Abbreviations

CLD: Causal loop diagram
FOMO: Fear of missing out
IED: Interactive electronic device
SHINE: Schools Health and Wellbeing Improvement Research Network
SHRN: School Health Research Network
ToC: Theory of change
UK: United Kingdom
