# Supplementary material for: Developing a systems-based programme theory for reducing night-time interactive electronic device use and improving sleep among adolescents
Source: PLoS One. 2026 Oct 21;21(10):e0359723. doi: 10.1371/journal.pone.0359723 (PMC13686474; doi:10.1371/journal.pone.0359723)
Supplement: Supplementary file 1 [file pone.0359723.s001.pdf]

S1: Overview of CLOCK OFF components

CLOCK OFF is an 8-week school-based intervention designed to improve adolescent sleep habits and reduce night-time use of IEDs and social media. Designed to be delivered in partnership with a specialist sleep charity, the intervention consists of four main components.

Teacher training

Teachers receive face-to-face training on adolescent sleep, sleep health, and evidence-based strategies to support healthy sleep behaviours. This prepares them to implement the intervention through the curriculum.

Teacher-led classroom lessons

Schools deliver up to three curriculum-based lessons covering the importance of sleep, adolescents' sleep needs, body clocks and circadian rhythms, factors affecting sleep quality, and the impact of evening device and social media use. Lessons include pupil feedback and reflection exercises, including discussion activities, surveys and practical behaviour-change tasks.

Parent information

Schools are provided with a digital information brochure to share with parents. It aims to increase parental awareness of healthy sleep practices and support positive bedtime routines.

Peer support programme

Influential pupils are identified through a peer nomination process and trained as peer supporters. Training provides knowledge about healthy sleep and the effects of night-time device use, alongside communication and interpersonal skills. Following training, peer supporters engage in informal conversations with their peers to promote healthy sleep behaviours. Follow-up sessions reinforce knowledge, confidence and message dissemination.

Note a full intervention description will be published in a forthcoming article.
