# Supplementary material for: Developing a systems-based programme theory for reducing night-time interactive electronic device use and improving sleep among adolescents
Source: PLoS One. 2026 Oct 21;21(10):e0359723. doi: 10.1371/journal.pone.0359723 (PMC13686474; doi:10.1371/journal.pone.0359723)
Supplement: Supplementary file 2 [file pone.0359723.s002.pdf]

S2: Overview of the ASSIST, Sound Sleep and #Sleepyteens

ASSIST

The ASSIST (A Stop Smoking in Schools Trial) was an effective peer-led anti-smoking intervention in which 'influential' young people (aged 12-13 years) were recruited via peer nomination and trained as peer supporters over a two-day period. The training informed adolescents about the risks of smoking and the benefits of living 'smoke-free', alongside communication skills. These trained peer mentors would then spread and sustain non-smoking norms through informal interactions [1]. This approach has been adapted to sexual health behaviour in the STASH trial and substance use in the Frank Friends trial [2, 3].

Sound Sleep

The Sound Sleep programme [4] is an initiative from the charity Sleep Action (formerly Sleep Scotland), which aims to educate pupils from both primary and secondary schools about the impact of sleep on their health, empowering them to recognise their own sleeping behaviour and to make personal changes to improve their sleep duration and quality through cognitive-behavioural techniques. The programme consists of training from Sound Sleep trainers (which covers various evidence-based topics including the function of sleep, circadian rhythms, sleep and wellbeing, dreams, sleep disorders, the influence of stimulants and sedatives, and adolescent sleep; a Sound Sleep resource pack for educators to build their capacity to independently deliver programme material in education settings over time (including lesson plans, MS PowerPoint presentations and activity handouts, such as quizzes and sleep diaries); a Sound Sleep Ambassador (*e.g.* a teacher, school nurse, parent or volunteer within the school) working in tandem with a group of older pupils aged 16-18 years. Together, the Ambassadorial team are trained to raise awareness about the importance of sleep and assist pupils with sleep problems, while being able to signpost to relevant services as appropriate.

#Sleepyteens

The #Sleepyteens project has been working in partnership with schools to support evidence-based approaches to social media use and sleep since 2014. Participating pupils have highlighted that an effective approach to promoting a healthy balance must approach social media as a social interaction, with expectations around availability and prompt responses, as well as fears over missing out [5]. Indeed, pupils who experience concerns about staying connected and following etiquette more strongly also report a range of poorer sleep outcomes [5]. Building on this evidence from online surveys with over 3000 pupils, the project has developed a programme of lesson materials that focus on pupil-led discussions of local evidence gathered in an online survey in the first session. Teachers use the survey results to facilitate discussions around the perceived impact of social media on sleep, as well as demonstrating techniques to identify and reframe unhelpful worries about disconnecting at night. Evaluation has indicated that this programme increased knowledge of the links between social media use and sleep, as well as promoting behaviour change and discussions with peers and family [6].

References

1. Campbell R, Starkey F, Holliday J, Audrey S, Bloor M, Parry-Langdon N. et al. An informal school-based peer-led intervention for smoking prevention in adolescence (ASSIST): a cluster randomised trial. Lancet. 2008;371:1595-1602.

2. Forsyth R, Purcell C, Barry S, Simpson S, Hunter R, McDaid L, et al. Peer-led intervention to prevent and reduce STI transmission and improve sexual health in secondary schools (STASH): protocol for a feasibility study. Pilot Feasibility Stud. 2018;4:180
3. White J, Hawkins J, Madden K, Grant A, Er V, Angel L, et al. (2017). Adapting the ASSIST model of informal peer-led intervention delivery to the Talk to FRANK drug prevention programme in UK secondary schools (ASSIST + FRANK): intervention development, refinement and a pilot cluster randomised controlled trial. Public Health Res. 2017;5:17.
4. Sleep Action. Sound Sleep. Online. 2024. Available from: https://sleepaction.org/training/education/for-schools Accessed 9 December 2024
5. Scott H, Biello SM, Woods HC. Nodding off but can't disconnect: development and validation of the iNOD index of Nighttime Offline Distress. Sleep Med. 2021;81:430-8.
6. Woods, H. C., & Scott, H. #Sleepyteens: Social media use in adolescence is associated with poor sleep quality, anxiety, depression and low self-esteem. J Adolesc. 2016;51:41-9.
