# Supplementary material for: Developing a systems-based programme theory for reducing night-time interactive electronic device use and improving sleep among adolescents
Source: PLoS One. 2026 Oct 21;21(10):e0359723. doi: 10.1371/journal.pone.0359723 (PMC13686474; doi:10.1371/journal.pone.0359723)
Supplement: Supplementary file 3 [file pone.0359723.s003.pdf]

S3: Workshop guides

Causal loop diagram building workshops

What are we doing and why?

We will invite young people to attend a participatory systems mapping workshop to better understand young people's views on factors involved in night-time use of interactive electronic devices and the connections between these factors.

Problem

Reports show young people get less than the recommended number of hours of sleep. The impact of this can be physiological and psychological, which can in turn impact on external factors such as schoolwork or relationships. Over recent decades, device use has become an integral part of young people's development and ability to communicate with others. Increases in interactive electronic device use have run in parallel with a shift in sleeping habits, yet there is still relatively limited knowledge of the implications of access to, and use of, interactive electronic devices on sleep outcomes.

Questions of interest

- What factors influence the use of interactive electronic devices at bedtime/night time?
- What factors could be potential leverage points in developing an intervention?
- What are the potential causal mechanisms, barriers and facilitators, and motivational factors associated with changes in interactive electronic device use at bedtime/night time?

Main outputs

- We will produce a list of factors that influence device use and sleep
- We will start to produce a draft causal loop map including the identified factors

General structure of CLOCK OFF workshops

We will start with an introduction to the workshop, the research team and the participants on the call, and include an ice-breaker activity. The workshop will consist of interactive group activities. Depending on the number of attending participants, activities will take place in smaller breakout groups with no more than 5 participants in each group. Groups for activity 1 and 2 remain the same. There will be a comfort break to reduce screen time. Note takers will support the facilitation and use of interactive software. After the group activities, findings will be shared between groups in plenary sessions. It will be video recorded using the videocall software's functions to capture the screen-shared interactive development. Participants will be advised that if they do not want to be on screen, they can turn their video function off, but audio will be captured. However, depending on the infrastructure in the participants' school, they might be on a shared device, which may preclude the option to turn their camera off. All efforts will be taken to make participants comfortable.

Duration

Workshop will last up to 90 minutes.

Where are we doing this?

Online using MS Teams and StickyStudio.

Activity 1: variable elicitation

Working in breakout groups (if required). Young people will be asked to list factors involved in night-time use of social media and interactive electronic device use. Guiding questions will be used (*e.g.* why do young people use social media and IED when in bed; what do they tend to use devices/apps for; and what stops them from switching off?). We will use Stickystudio interactive whiteboards to capture young people's input.

Activity 2: Causal loop diagram

This activity will be broken down into two parts. Working in breakout groups (if required), the Stickystudio software will be used to build a causal loop diagram connecting the factors identified in Activity 1 in relation to the priority outcomes identified by the participants. Young people will be asked to identify the direction of connection and whether the factors change in the same or opposite direction (*e.g.* does an increase in factor 1 lead to an increase in factor 2?). Participants will be able to create the map, although the mapper will primarily do this.

Participant numbers

Maximum of 10 participants per workshops. With 10 participants, this means two breakout rooms (maximum of 5 per room). Depending on recruitment, breakout rooms should not have less than 4 participants per room. Seven is maximum number of participants without breakout rooms.

Research staff required

- If 10 participants = minimum of 4 research staff = 2 mappers and 2 facilitators
- If 5 participants = minimum of 2 research staff = 1 mapper and 1 facilitator
- Potential professional service assistance required if 10 participants. This is to reallocate participants back into breakout rooms in case of accidental removal

Research staff tasks

- Lead facilitator is the focal point to lead workshops, introductions, expectations, questioning and debriefs
- Mapper – to move factors between platforms (activity 1) and to connect links in mapping process (activity 2)
- Facilitator – prompt and lead discussion in activities 1 and 2

Outline workshop schedule

	Tasks	Time	Materials / facilitator notes
**Welcome and overview of workshop activities**	1. Welcoming all attending<br>2. Expectations for the workshop<br>3. Introductions<br>- Name<br>- Age<br>- Hobby	10 mins	• Facilitator to lead.<br>• PowerPoint slides.<br>• Outline information for participants: timings/video recording reminder/ two main activities/breaks.<br>• Discuss how this is a research project and how important it is to remember that nothing said here will be seen by other people (privacy and confidentiality)<br>• Tutorial on how to navigate Stickystudio (*e.g.* using sticky notes, text boxes)<br>• Share Stickystudio link – participants to practice using Stickystudio
**Introduction to topic of project**	Introduction to study<br><br>**Voting question:** What is more important to you?<br>• Longer sleep<br>• Falling asleep quicker<br>• Not waking up in the night<br>• Feeling rested in the morning<br><br>**Voting question:** Is a lack of sleep a problem to you?<br>• Yes<br>• No<br>• Why?	10-15 mins	• Facilitator to lead<br>• Brief discussion on sleep, what we know it is good for, what happens when we do not get enough sleep<br>• Find out what is important to the adolescents using voting questions and sticky notes/dots
**Activity 1: Variable elicitation**	Introduce activity.<br><br>Random allocation to 2 breakout groups (if required).<br><br>1 facilitator and 1 mapper per group.	30 mins	• Facilitator to lead; supported by mapper<br>• PowerPoint slides<br>• Facilitators to help stimulate discussion or ideas<br>• Facilitators to write down ideas if young people struggle using Stickystudio

	In Stickystudio area, populate the following with sticky notes:<br>1. Electronic device (mobile phones, tablets, laptops) and social media use. What do you use it for at night / bedtime?<br>2. Why do you use it at night / bedtime?<br>3. What factors affect your sleep for better or worse?<br>4. What makes or stop using your device and what is the effect?		• Let young people come up with their own factors (emphasis on quantifiable where appropriate / possible – adjusted by mapper if required)<br>• Prompt for barriers, facilitators, impact, motivation
Break		10 min	Mappers to move variables into required platform. Add another break during next session if required.
**Activity 2: Causal loop diagram building**	1. Introduce task<br>2. Place in same breakout groups if required<br>3. Connecting factors using arrows function in Stickystudio	30 mins<br><br>Practice (10 mins)	• During first activity mappers will be watching and making notes of what is said and then moving items into new Stickystudio area ready for activity 2.<br>• Facilitator to lead with input from mappers if and when required.<br>• PowerPoint: provide picture of casual loop diagram and a demonstration of connecting factors (taking part in study example). Explain why we create CLDs.<br>• Ideally, mapper will only draw connections between factors that young people discuss.<br><br>• After practice, start connecting factors. Mapper will have moved all factors into required Stickystudio area and grouped them if required.<br>• Mapper will draw connections directed from young people.

			• Facilitator to prompt discussion with young people, exploring where the links go and in which direction.
**Plenary outcome**	1. Wrap up and share maps between groups (if required)<br>2. What will happen next<br>3. Thank you and date of next workshop	5 mins	• Facilitator to lead.<br>• PowerPoint slide<br>• Thank you for all the hard work. Share created work if required.<br>• Power point slide of flow chart of where this information is feeding into the study process.<br>• Tell participants when next workshop takes place and offer thanks.

Causal loop diagram validation workshop 1 (adolescents)

What are we doing and why?

We will invite young people to attend two validation workshops to help consolidate previous system map building work, and ensure it reflects their perceptions of the system of factors that influence bedtime/night time use of interactive electronic devices, and the connections between these factors.

Duration

Workshop will last up to 90 minutes.

Where are we doing this?

Online using MS Teams and StickyStudio.

Activity 1: soft validation

Guiding questions will be used (*e.g.* why do young people use social media and IED when in bed; what do they tend to use devices/apps for; and what stops them from switching off?). We will use Stickystudio interactive whiteboards to capture young people's input.

Activity 2: querying factors

Young people will be introduced to the idea of causal loop diagram, why we create them, and what they look like. It will be explained that sometimes factors in system maps do not always make sense or do not fit into a plausible narrative of system dynamics. Therefore, this task will be to look at factors and see if they make sense to young people as key influences of their interactive electronic device use. The factors the young people will be seeing will have been generated from peers in previous workshops. Screen shots of factors will be shown and an open discussion will be held.

Participant numbers

6 participants will be present. This number is manageable with no breakout rooms, based on previous workshops with similar numbers.

Research staff required

Two research staff will be present.

Research staff tasks

- Lead facilitator is the focal point to lead workshops, introductions, expectations, questioning and debriefs
- Note taker/mapper – to take notes where required and to provide read through of causal loop diagram

Outline workshop schedule

	Tasks	Time	Materials / facilitator notes
**Welcome and overview of workshop activities**	1. Welcoming all attending<br>2. Expectations for the workshop<br>3. Introductions<br>- Name<br>- Age<br>- Hobby	5 mins	• Facilitator to lead<br>• Power point slides<br>• Welcomes<br>• Ice breaker<br>• Outline information for participants: timings/video recording reminder/ two main activities/breaks.<br>• Discuss how this is a research project and how important it is to remember that nothing said here will be seen by other people (privacy and confidentiality)
**Start recording here**			
**Introduction to topic of project**	Introduction to study and opening questions / discussion on sleep.<br><br>What are we doing in the study?<br>What have we done so far?<br>What will you be doing to help?	10 mins	• Facilitator to lead<br>• Quick discussion on sleep: the evidence suggests that lack of sleep is a problem. We also know that devices are becoming more popular.<br>• What is sleep for? Why is it important to get enough sleep?<br><br>1. CLD mapping<br>2. ASSIST<br>3. #sleepyteens<br>4. Logic model<br><br>• Explanation of how they will help (slides).
**Activity 1:**	Soft validation:<br><br>Electronic device (mobile phones, tablets, laptops) and social media use.<br><br>Defining night time/bedtime use. 1hr before bed?	<20 mins	• Facilitator to lead<br>• Power point slides<br>• Facilitators to help stimulate discussion or ideas.<br>• Let young people come up with their own factors (emphasis on quantifiable where appropriate / possible – adjusted by mapper if required)

	1. What do you use it for at night / bedtime?<br><br>2. What is the impact of a poor night's sleep?		*There are two additional questions which can be asked if required. These were asked in the original workshops and can be checked if required (what factors affect your sleep for better or worse? and what makes or stop using your device and what is the effect?).*
Break		10 mins	
**Activity 2: Query links**	Introduction to main system map to demonstrate what has been made.<br><br>1. Introduce task<br>2. Query factors (x 3 pictures)	30 mins	• Facilitator to show main system map to demonstrate what the previous participants helped make.<br>• Team to explain why we have made a map (potentially show slide that has the explanation how map works).<br>• But some factors we could not make links between. Today we are going to look at the links that did not get included in the main map, to see if they might be important enough to be included.<br>• Open discussion on each query.
**End recording here**			
**Ending**	1. Wrap up<br>2. What will happen next?<br>3. Thank you and date of next workshop	5 mins	• Facilitator to lead<br>• PowerPoint slide<br>• Thank you for all the hard work. Share created work if required<br>• Power point slide of flow chart of where this information is feeding into the study process<br>• Tell participants when next workshop takes place and offer thanks

Causal loop diagram validation workshop 2 (adolescents)

What are we doing and why?

We will invite young people to attend two validation workshops to help consolidate previous system map building work, and ensure it reflects their perceptions of the system of factors that influence bedtime/night time use of interactive electronic devices, and the connections between these factors.

Duration

Workshop will last up to 90 minutes.

Where are we doing this?

Online using MS Teams and StickyStudio.

Activity 1: Feedback loop querying and validation

Young people will be re-introduced to the idea of causal loop diagrams, why we create them and what they look like. The participants will be walked through several feedback loops to establish the following:

- Do these factors reflect reality / do they make sense, is this similar to your experience?
- Is anything unclear?
- Is there anything incorrect?
- Are any factors missing?
- Are any links missing?

Participant numbers

Six participants will be present. This number is manageable with no breakout rooms, based on previous workshops with similar numbers.

Research staff required

- 2 research staff will be present

Research staff tasks

- Lead facilitator is the focal point to lead workshops, introductions, expectations, questioning and debriefs
- Note taker/mapper – to take notes where required and to provide read through of causal loop diagram

Outline workshop schedule

	**Tasks**	**Time**	**Materials / facilitator notes**
**Welcome and overview of workshop activities**	1. Welcoming all attending<br>2. Expectations for the workshop<br>3. Introductions<br>- Name	5 mins	• Facilitator to lead<br>• Power point slides<br>• Welcomes<br>• Ice breaker

<table>
<tr><td></td><td>- Age
- Hobby</td><td></td><td>• Outline information for participants: timings/video recording reminder/ two main activities/breaks
• Discuss how this is a research project and how important it is to remember that nothing said here will be seen by other people (privacy and confidentiality)</td></tr>
<tr><td colspan="4">Start recording here</td></tr>
<tr><td>Activity 1:</td><td>Re-introduction to system map and how it works.

Work through a number of loops

1. Device use vs parental controls
2. Device use vs worry
3. Device use vs emotional dissatisfaction (nosiness vs different uses)
4. Device vs repeat use to energy
5. Device use vs noisy notifications</td><td>30 mins</td><td>• Facilitator to lead
• Mapper to assist with note taking and ‘story telling’
• Power point slides

Questions of interest and focus:
• Do these factors reflect reality / do they make sense, is this similar to your experience?
• Is anything unclear?
• Is there anything incorrect?
• Are any factors missing?
• Are any links missing?</td></tr>
<tr><td colspan="2">Break</td><td>10 mins</td><td></td></tr>
<tr><td>Activity 1: Continued</td><td>Work through remaining loops and sub maps:

1. Positive energies loop
2. Negative energies loop
3. Sub map controls
4. Sub map controls
5. Sub map emotions</td><td>30 mins</td><td>• Facilitator to lead
• Mapper to assist with note taking and ‘story telling’
• Power point slides

Questions of intertest and focus
• Do these factors reflect reality / do they make sense, is this similar to your experience?
• Is anything unclear?
• Is there anything incorrect?
• Are any factors missing?
• Are any links missing?</td></tr>
<tr><td colspan="4">End recording here</td></tr>
<tr><td>Ending</td><td>1. Wrap up</td><td>5 mins</td><td>• Facilitator to lead</td></tr>
</table>

	2. What will happen next<br>3. Thank you and date of next workshop		• PowerPoint slide<br>• Thank you for all the hard work. Share created work if required<br>• Power point slide of flow chart of where this information is feeding into the study process<br>Tell participants when next workshop takes place and offer thanks

Causal loop diagram validation workshop 3 (parents/guardians and teachers)

What are we doing and why?

We will invite stakeholders (parents and school staff) to attend a system mapping workshop to better understand the system of factors influencing young people's bedtime/night time interactive electronic device use.

What did the previous young people's workshops cover?

We conducted four workshops with young people to: 1) create a system map of device use at night time; 2) consider adaptations of established interventions (ASSIST, Sound Sleep and #Sleepyteens); and 3) refine our programme thinking by reviewing a logic model. Adult stakeholders have been made aware of this.

What did the previous adult workshops cover?

We conducted two workshops with adults looking at what adaptations of established interventions (ASSIST, Sound Sleep and #Sleepyteens) could be made. Additionally, we looked to refine our programme thinking by reviewing a logic model and held an open discussion on what changes could be implemented.

What will this workshop do and produce?

This workshop will allow researchers to explore similar questions that were presented to young people, but from an adult perspective. Furthermore, we will target parents and school staff to allow for more focused contextual questions to help us better understand the system.

Duration

Workshop will last up to 90 minutes.

Where are we doing this?

Online using MS Teams and StickyStudio.

Activity 1: Variable elicitation

Working in breakout groups (if required). Adult stakeholders will be asked to list factors influencing night time use of social media and interactive electronic devices. Guiding questions will be used (described below in table).

Activity 2: Causal loop diagram validation

Participants will be guided through a series of submaps depicting feedback loops. Group discussions will be held to ascertain whether the narrative of these submaps is consistent with adult stakeholders' perceptions. Additional factors and connections for inclusion in the final causal loop diagram will be noted.

Participant numbers

It is anticipated that there will be around 7 participants. An upper limit of 10 participants will be in place.

Research staff required

- This workshop will run with 2 research staff (a lead facilitator and a mapper/notetaker)

Research staff tasks

- Lead facilitator is the focal point to lead workshops, introductions, expectations, questioning and debriefs. They will also monitor the chat function if there is a need.
- Mapper will make written observations related to the causal loop diagram, and ask questions if they consider there has been a missed opportunity to explore a topic. They will also monitor the chat function if there is a need.

Outline workshop schedule

	**Tasks**	**Time**	**Materials / facilitator notes**
**Welcome and overview of workshop activities**	1. Welcoming all attending<br>2. Expectations from workshop<br>3. Intro to StickyStudio	5 mins	• Facilitator to lead.<br>• PowerPoint slides.<br>• Outline information for participants: timings/video recording reminder/ two main activities/breaks.<br>• Discuss how this is a research project and how important it is to remember that nothing said here will be seen by other people (privacy and confidentiality)<br>• Tutorial on how to navigate Stickystudio (*e.g.* using sticky notes, text boxes)<br>• Share Stickystudio link – participants to practice using Stickystudio
**Activity 1: Variable elicitation**	Introduce activity and what a causal loop diagram is.<br><br>1. What influences night time use of devices/social medial use?<br><br>2. What policies/processes/ actions do schools have toward supporting the reduction of electronic device (mobile phones, tablets, laptops) and social media use at night time?	30 mins	Focus to help stimulate discussion or ideas. Emphasis on quantifiable factors where appropriate / possible.<br><br>1. What to consider: some aspects that are both broad and those that might impact at a more personal level. Macro, meso, micro levels – (*e.g.* adverts, trends, friends, sports clubs).<br><br>2. This could be your school or schools you are aware of. Parents

	3. What factors do you think as a teacher/school you can influence? How can parents support what is going on at school?		– are you aware of what your child's school has?<br><br>3. What can you support parents with? / Parents, what can you support school with?<br><br>**Areas to explore:**<br>Barriers, facilitators, impact, motivation, commonalities and differences, time (*e.g.* negotiating with child at night to ensure phone turned off), social costs (*e.g.* strain on relationships when trying to get children to sleep), other device use (e.g., writing in school vs typing).
Break		10 min	Facilitators: to move variables into required platform
**Activity 2: Unpicking Causal loop maps**	Introduction to system map and how they work.<br><br>Work through several loops:<br><br>1. Conscience submap<br>2. Education submap<br>3. FOMO submap<br>4. Social Submap<br>5. Reinforcing loop<br>6. Balancing loop	35 mins	• Facilitator to lead<br>• Mapper to assist with note taking and 'story telling'<br>• Power point slides<br><br>Questions of intertest and focus:<br>• Do these factors reflect reality / do they make sense, is this similar to your experience?<br>• Is anything unclear?<br>• Is there anything incorrect?<br>• Are any factors missing?<br>• Are any links missing?
**Ending**	1. End – what happens next?<br>2. Any questions?<br>3. Thank you and vouchers	5 mins	PowerPoint slide
