# Supplementary material for: Developing a systems-based programme theory for reducing night-time interactive electronic device use and improving sleep among adolescents
Source: PLoS One. 2026 Oct 21;21(10):e0359723. doi: 10.1371/journal.pone.0359723 (PMC13686474; doi:10.1371/journal.pone.0359723)
Supplement: Supplementary file 4 [file pone.0359723.s004.pdf]

S4: inputs and activities of the CLOCK OFF intervention

Inputs

- Curriculum based content
- Information material for parents
- Lesson materials and teaching plan for teachers
- Peer nomination survey
- Sleep and social media survey

Activities

- Advice from trusted adults
- Cognitive reframing
- Diffusing sleep promoting messages
- Exposure to educational content
- Homework – parent involvement
- Learning
- Lessons on sleep and social media
- Modelling of desired device use behaviour
- Notifications
- Ongoing support for peer supporters
- Parental/guardian controls
- Parental/guardian engagement with curriculum content
- Peer supporter training
- Peer supporters offer social support to pupils
- Positive offline social interactions
- Prioritising sleep over other activities
- Prolonged device use
- Repeat interactive electronic device use
- Seeking peer contact
- Technological controls
