# Supplementary material for: Developing a systems-based programme theory for reducing night-time interactive electronic device use and improving sleep among adolescents
Source: PLoS One. 2026 Oct 21;21(10):e0359723. doi: 10.1371/journal.pone.0359723 (PMC13686474; doi:10.1371/journal.pone.0359723)
Supplement: Supplementary file 5 [file pone.0359723.s005.pdf]

S5: Outputs 1 and 2

Output 1: Presentation of the adolescent validated CLD

The adolescent validated CLD is available **here**. This figure illustrates the complex causal relations in the system for adolescent night-time interactive electronic device use and sleep, as perceived by adolescents. The map contained 48 factors. Among these, factors were categorised as behavioural (n = 17), educational (n = 4), environmental (n = 1), physiological (n = 5), psychological (n = 19), social (n = 9) and technological (n = 8). Between factors, there were 94 connections. These are depicted as solid arrows (representing a positive (+) relation between two factors, where change in one factor leads to a change in a second factor in the same direction, *i.e.* both increase or both decrease) and dashed arrows (representing a negative (-) relation, whereby a change in one factor leads to an inverse change in a second factor, *i.e.* an increase in one leads to a decrease in another, or vice versa).

Participants raised and discussed several factors that may be of particular relevance to, or amenability to influence through, peer-influence interventions such as CLOCK OFF. These included 'positive school experiences', which adolescents associated with having enough energy after a night's sleep. Although not explicitly discussed by our sample, the opportunity to help engineer a positive social environment in schools is integral to our project's aims. Relatedly, 'positive offline social interactions' was a key factor in numerous feedback loops we identified, and is discussed further below. 'Feelings of being in the loop' (*i.e.* having information about, or being part of decision-making in, social groups) and 'comparison of self with peers' (*i.e.* perceptions of other adolescents' behaviours and activities developed through device use) were two factors influencing 'FOMO' (*i.e.* 'fear of missing out') among are participants. Combined, these three factors may also be of focal interest in a peer-influence intervention.

The adolescent validated map included 382 feedback loops, ranging from six reciprocal relations between a pair of factors, up to a single chain of 14 factors. After sense-checking each loop manually for plausibility of causal logic, analysing them against fieldnotes and findings from across the CLOCK OFF project, and discussing these with adolescents, we selected six key feedback loops that are perceived by young people to drive system dynamics for night-time use of interactive electronic devices. Among these, three were balancing loops (B*n*) and three were reinforcing loops (R*n*). Table 1 describes the connections and dynamics of these loops.

Table 1. Key feedback loops in the adolescent validated CLD

No.	Connections	Description and interpretation
B1	Use of device at bedtime (+)→ parental/guardian controls (-)→ use of device at bedtime	When adolescents use their devices at bedtime, this increases the amount of non-technological controls that parents/guardians use to manage this behaviour. Where successful, these controls have a balancing effect and can reduce use of devices at bedtime.
B2	Use of device at bedtime (+)→ repeat interactive electronic device use (+)→ parental/guardian controls (+)→ relaxation (+)→ sleep quality/duration (+)→ energy (+)→ positive offline social interactions (-)→ FOMO (+)→ use of device at bedtime	When adolescents use their devices at bedtime, this increases the likelihood of repeat behaviour (potentially becoming habituated). In response, parents/guardians increase their use of non-technological controls to reduce or interrupt this behaviour. Where successful, these controls can lead to increased relaxation around bedtime, which in turn improves sleep outcomes. The effect of increased sleep quality/duration is that adolescents have increased energy, which enable increases in positive offline social interactions (*e.g.* at school). This helps reduce experiences of FOMO, which has a balancing effect by reducing use of devices at bedtime.
B3	Use of device at bedtime (+)→ repeat interactive electronic device use (+)→ parental/guardian controls (+)→ sleep promoting bedtime routines (+)→ relaxation (+)→ sleep quality/duration (+)→ energy (+)→ positive offline social interactions (-)→ use of device at bedtime	When adolescents use their devices at bedtime, this increases the likelihood of repeat behaviour (potentially becoming habituated). In response, parents/guardians increase their use of non-technological controls to reduce or interrupt this behaviour. Where successful, these controls can lead to increased sleep promoting bedtime routines, and in turn relaxation around bedtime. This improves sleep outcomes. The effect of increased sleep quality/duration is that adolescents have increased energy, which enable increases in positive offline social interactions (*e.g.* at school). This helps reduce experiences of FOMO, which has a balancing effect by reducing use of devices at bedtime.
R1	Use of device at bedtime (+)→ worry (+)→ use of device at bedtime	When adolescents use their devices at bedtime, this can lead to increased levels of worry due to content they access. This has a reinforcing effect and leads to increased use of devices.
R2	Use of device at bedtime (+)→ emotional dissatisfaction (+)→ use of device at bedtime	When adolescents use devices at bedtime, this leads to an increase in emotional dissatisfaction, which in turn has a reinforcing effect on further increases in device use.
R3	Use of device at bedtime (+)→ emotional dissatisfaction (-)→ happiness (+)→ sleep quality/duration (+)→ energy (+)→ positive offline social interactions (-)→ use of device at bedtime	When adolescents use devices at bedtime, this leads to an increase in emotional dissatisfaction. As such, this causes adolescents to feel less happy. A reduction in happiness also reduces sleep outcomes. Consequently, adolescents have less energy and this leads to a reduction in positive offline social interactions. Combined this chain of causal effects can result in reinforcing increased device use.

Across the balancing feedback loops, parental/guardian controls (*e.g.* device curfews or time limits for device use) was a consistent factor, which reflects adolescents' views of the importance of their primary care givers in influencing their device use and sleep-related behaviours. Loop B1 illustrates the basic dynamics of the relation between bedtime device use and non-technological parental/guardian controls. However, as indicated by loops B2 and B3, there are multiple pathways by which controls may ultimately lead to a balancing effect on device use. Loops B2 and B3 both relate to the idea of 'positive energies', whereby controls leading to improved sleep outcomes have the effect of increased energy levels among adolescents. When energised, adolescents are potentially more able to have meaningful and productive offline social interactions (*e.g.* at school) and as such, feel in-touch with social developments, experience less fear of missing out (FOMO), and may therefore feel a reduced need to use their devices around bedtime. Loop B3 differs from B2 by the inclusion of an additional factor (sleep promoting bedtime routines) between parental/guardian controls and relaxation.

In terms of the reinforcing loops, the inverse of B2 and B3 is presented in loop R3 ('negative energies'), whereby causal processes may lead to adolescents experiencing less energy as a result of the detrimental effects of device use and subsequent emotional reactions, which lead to poorer sleep outcomes. This reduction in energy has the opposite effect of that already described; adolescents may have less positive offline social interactions during the day, thus turn toward their phones to meet their social needs. In the second validation workshop, we asked adolescents about the relationship between device use and worry. Prior to this workshop it was unclear whether worry driven by device use reduced or reinforced further use. Our participants indicated the latter causal process was more likely, as exposure to worrying content increased the likelihood that they would talk to their friends about what they had seen (R1). Loop R2 illustrates the basic dynamics of the relation between device use and emotional dissatisfaction. Adolescents discussed how, depending on duration and type of use, using their devices could lead to a range of affective reactions (*e.g.* boredom, frustration), which means they use their phones for alternative reasons to alleviate these negative emotions. Our discussions highlighted how this basic feedback loop can route through different causal pathways. For example, adolescents described a general sense of nosiness as a factor related to boredom that they perceived to drive device use among their age group.

Output 2: Presentation of the parent and school staff validated CLD

The second iteration of the CLD is available **here**. Following the validation workshop with parents and school staff, the refined system map comprised 55 factors and 107 connections. At this stage, original factor labels were retained. The factors were categorised as behavioural (n = 19), educational (n = 9), environmental (n = 3), physiological (n = 5), psychological (n = 21), social (n = 10) and technological (n = 9). While participants did not recommend the removal of any factors, activities elicited seven new factors. These were 'addictive qualities of content', 'curriculum-based content', 'good parent/guardian-child relationships', 'offline schoolwork', 'perceived device use by peers', 'school restrictions on device use' and 'time available for daytime online activities'. Following this third validation workshop, two connections from the adolescent map were removed following recommendations from the participants; school staff were unable to propose an explanation for the perceived direct positive relation between 'teacher knowledge' and 'sleep quality/duration', nor the direct negative relation between 'use of device at bedtime' and 'teacher knowledge'. Sixteen new connections were made, which linked both new and existing factors. Here is a list of all changes made between the two validated versions of the system map:

Changes between adolescent validated map and adult validated map

Factors added to the map

Addictive qualities
Curriculum-based content
Good parent-child relationships
Offline school work
Perceived device use of peers
School restrictions on device use
Time available for daytime online activities

Connections added to the map

Ability to function (+)→ Mental health
Addictive qualities of content (-)→ Agency
Advice from trusted adults (+)→ Awareness of the effect of device use at bedtime
Curriculum-based content (+)→ Teacher knowledge
Curriculum-based content (+)→ Advice from trusted adults
Curriculum-based content (+)→ Awareness of the effect of device use at bedtime
Emotional dissatisfaction (-)→ Good child-parent relationships
Fatigue (-)→ Use of device at bedtime
Offline schoolwork (-)→ Use of device at bedtime
Overstimulation (+)→ Parental/guardian controls
Perceived device use of peers (+)→ Emotional dissatisfaction
School restrictions on device use (-)→ Time available for daytime online activities
Teacher knowledge (+)→ Parental/guardian controls
Teacher knowledge (+)→ Education from parents/guardians
Technological controls (-)→ Background operations of devices
Time available for daytime online activities (+)→ Use of device at bedtime

Connections removed from the map

Teacher knowledge (+)→ sleep quality/duration
Use of device at bedtime (-)→ teacher knowledge

School staff and parent discussions led to the addition of several factors and connections related to education (*e.g.* school rules and homework) and parents/guardians (*e.g.* child-parent/guardian relationships). However, this additional validation phase highlighted the important influence of school-based factors, parents/guardians and the home environment, as well as macro-level factors (*e.g.* national curriculum content/the addictiveness of social media and IED use). These findings provide important contextual information relevant to the implementation of the CLOCK OFF intervention.

The full adult validated map included 1,003 detected feedback loops, although none of these contained new factors added at the adult validation stage. The submap generated from the validated map (available **here**), including a consolidated list of 20 factors with the highest values across the four centrality measures, included 28 plausible feedback loops (13 balancing; 15 reinforcing). Loops, which ranged from 2 to 8 connections, are described in Table 2. Across the balancing feedback loops, 11 included the 'parental/guardian controls' factor, further emphasising the importance of primary care givers in influencing adolescent device use and sleep behaviours. However, loops B13 and B14 demonstrate how it is possible for such controls to lead to a negative emotional reaction and poorer sleep outcomes (potentially short-term), even if these may ultimately balance out in terms of heightened fatigue reducing device use at subsequent bedtimes. The reinforcing feedback loops broadly related to two main clusters of factors. First, the dynamics between 'sleep quality/duration' and changes in 'mental health'. Second, the affective dimensions of device use and sleep outcomes, whereby 'emotional dissatisfaction' and/or 'worry' (present in 8 of 15 reinforcing loops) contribute to causal processes of decreased 'sleep quality/duration'.

Feedback loops (adult validated map)

Balancing feedback loops	
**No.**	**Connections**
B1	Use of device at bedtime (+)→ Parental/guardian controls (-)→ Use of device at bedtime*
B4	Use of device at bedtime (-)→ Sleep quality/duration (-)→ Fatigue (-)→ Use of device at bedtime
B5	Use of device at bedtime (+)→ Repeat interactive electronic device use (+)→ Parental/guardian controls (-)→ Use of device at bedtime
B6	Use of device at bedtime (+)→ Worry (-)→ Sleep quality/duration (-)→ Fatigue (-)→ Use of device at bedtime
B7	Use of device at bedtime (+)→ Intrusive sensory experience (-)→ Sleep quality/duration (-)→ Fatigue (-)→ Use of device at bedtime
B8	Use of device at bedtime (+)→ Parental/guardian controls (+)→ Sleep promoting bedtime routines (-)→ Repeat electronic device use (+)→ Use of device at bedtime
B19	Use of device at bedtime (+)→ Parental/guardian controls (+)→ Relaxation (+)→ Sleep quality/duration (-)→ Emotional dissatisfaction (+)→ Use of device at bedtime
B10	Use of device at bedtime (+)→ Parental/guardian controls (+)→ Sleep promoting routines (+)→ Sleep quality/duration (-)→ Emotional dissatisfaction (+)→ Use of device at bedtime
B11	Use of device at bedtime (+)→ Repeat interactive electronic device use (+)→ Parental/guardian controls (+)→ Relaxation (+)→ Sleep quality/duration (-)→ Emotional dissatisfaction (+)→ Use of device at bedtime
B12	Use of device at bedtime (+)→ Repeat interactive electronic device use (+)→ Parental/guardian controls (+)→ Sleep promoting routines (+)→ Sleep quality/duration (-)→ Emotional dissatisfaction (+)→ Use of device at bedtime
B13	Use of device at bedtime (+)→ Parental/guardian controls (+)→ Sleep promoting routines (+)→ Relaxation (+)→ Sleep quality/duration (-)→ Emotional dissatisfaction (+)→ Use of device at bedtime
B14	Use of device at bedtime (+)→ Repeat interactive electronic device use (+)→ Parental/guardian controls (+)→ Emotional dissatisfaction (-)→ Sleep quality/duration (-)→ Fatigue (-)→ Use of device at bedtime
B15	Use of device at bedtime (+)→ Repeat interactive electronic device use (+)→ Parental/guardian controls (+)→ Sleep promoting routines (+)→ Relaxation (+)→ Sleep quality and duration (+)→ Emotional dissatisfaction (+)→ Use of device at bedtime

Reinforcing feedback loops	
**No.**	**Connections**
R1	Use of device at bedtime (+)→ Worry (+)→Use of device at bedtime*
R4	Mental health (+)→ Energy (+)→ Mental health
R5	Sleep quality/duration (+)→ Emotional dissatisfaction (+)→ Sleep quality/duration
R6	Sleep quality/duration (+)→ Mental health (+)→ Sleep quality/duration
R7	Use of device at bedtime (+)→ Repeat interactive electronic device use (+)→Use of device at bedtime
R9	Sleep quality/duration (+)→ Energy (+)→ Mental health (+)→ Sleep quality/duration
R9	Energy (+)→ Ability to function (+)→ Mental health (+)→ Energy
R10	Sleep quality/duration (+)→ Ability to function (+)→ Mental health (+)→ Sleep quality/duration
R11	Use of device at bedtime (+)→ Sleep quality/duration (+)→ Emotional dissatisfaction (+)→ Use of device at bedtime

R12	Sleep quality/duration (-)→ Emotional dissatisfaction (-)→ Enjoyment (+)→ Happiness (+)→ Sleep quality/duration
R13	Sleep quality/duration (+)→ Energy (+)→ Ability to function (+)→ Mental Health (+)→ Sleep quality/duration
R14	Use of device at bedtime (+)→ Worry (-)→ Sleep quality/duration (-)→ Emotional dissatisfaction (+)→ Use of device at bedtime
R15	Use of device at bedtime (+)→ Intrusive sensory experience (-)→ Sleep quality/duration (-)→ Emotional dissatisfaction (+)→ Use of device at bedtime
R16	Sleep quality/duration (-)→ Fatigue (-)→ Ability to function (+)→ Mental health (+)→ Sleep quality/duration
R17	Use of device at bedtime (+)→ Repeat electronic device use (+)→ Parental/guardian controls (-)→ Emotional dissatisfaction (+)→Enjoyment (+)→ Happiness (+)→ Sleep quality/duration (-)→ Fatigue (+)→ Use of device at bedtime

* Loop was identified as salient in the adolescent validated map
