# Supplementary material for: Developing a systems-based programme theory for reducing night-time interactive electronic device use and improving sleep among adolescents
Source: PLoS One. 2026 Oct 21;21(10):e0359723. doi: 10.1371/journal.pone.0359723 (PMC13686474; doi:10.1371/journal.pone.0359723)
Supplement: Supplementary file 6 [file pone.0359723.s006.pdf]

S6: Final CLOCK OFF CLD factor definitions and categories

Factor	Factor categories	Factor definition
Ability to function	Physiological<br>Psychological	The capacity to perform the activities and tasks that can normally be expected of them (*e.g.* engaging with the school day).
Ability to switch off	Behavioural	Young people are able to detach themselves from the stressors and cognitive workload associated with the day, which helps them relax and prepare for bedtime, and consequently the next day.
Addictive qualities of content	Psychological	Using interactive electronic devices (*e.g.* for social media) can lead to physical and psychological addiction by triggering the brain's reward system.
Advice from trusted adults	Educational<br>Social	Young people receive advice about using interactive electronic device from adults whom they trust (*e.g.* parents, other family members, coaches, teachers *etc.*)
Agency	Psychological<br>Social	Young people's ability to control their own behaviours and reactions to circumstances beyond their control, even if their actions are limited by someone or something else.
Awareness of effect of device use at bedtime	Psychological	Knowledge of the detrimental effects of using interactive electronic devices around bedtime, including the impact on sleep.
Background operations of devices	Technological	Apps on interactive electronic devices often continue to work 'in the background' without the active engagement of the user (*e.g.* the mechanisms by which push notifications are received).
Battery life	Technological	The length of time an interactive electronic device can continue to work before the battery needs to be recharged.
Breaking parents' or guardians' rules	Behavioural	The breaking of rules put in place by parents/guardians to limit the amount or type of interactive electronic device use.
Comparison of self with peers	Behavioural	Young people make comparisons with their peers' behaviours, activities, opportunities *etc.* through interactive electronic devices.

Factor	Factor categories	Factor definition
Concentration at school	Psychological	Young people's ability to maintain focus on school-based tasks, while ignoring distractions.
Curriculum-based content	Educational	The totality of what is to be taught in a school system, often mandated through statutes and other policies.
Desire to disconnect from content	Behavioural<br>Psychological	The intention of young people to actively disengage from or stop interacting with digital or electronic content.
Desire to wake up early	Behavioural<br>Psychological	Motivational state deriving from better sleep, and often associated with the need to undertake a particular task or activity.
Desire to wind down	Psychological	The motivation to engage in activities that help young people relax and prepare for sleep.
Device activations	Behavioural<br>Technological	The act of turning a device on.
Duration of device use	Behavioural	The length of time young people spend using electronic interactive devices.
Education from parents/guardians	Social	Developmentally appropriate information provided to, or facilitated with, young people by people in the role of parents/guardians.
Emotional dissatisfaction	Psychological	Young people may display a range of emotional responses (*e.g.* boredom, frustration, general negative emotions) when things happen that are unexpected/undesired.
Energy	Psychological	An individual's potential to perform mental and/or physical activities.
Energy expenditure	Physiological	Refers to the amount of energy an individual uses to maintain essential body functions (*e.g.* respiration, circulation, digestion) and as a result of physical activity.
Engagement with emotion-triggering content	Behavioural	Young people's involvement or interaction with content on devices that elicit emotional reactions, whether positive or negative.
Enjoyment	Behavioural	The positive feelings and satisfaction.
Exposure to distressing content	Behavioural<br>Psychological<br>Technological	Young people see or hear content on their devices that is distressing (*e.g.* age-inappropriate content, violence, hatred, emergencies *etc.*), but may also include misinformation and rumours (*e.g.* related to current affairs, as opposed to rumours about social contacts).
Exposure to educational content	Educational<br>Technological	Being presented with materials or media that are designed to inform, instruct, or provide knowledge to young people (*e.g.* educational apps, tutorial videos, websites).
Exposure to peer online activities	Social<br>Technological	The extent to which young people are exposed to or take part in online activities of their peers (*e.g.* social media usage or gaming *etc.*).

Factor	Factor categories	Factor definition
Fatigue	Physiological<br>Psychological	Subjective perceptions of reduced mental or physical capacity, a persistent sense of physical, emotional, and/or cognitive tiredness or exhaustion.
Fear of getting caught	Psychological	Young people may (be tempted to) use their device(s) at times that they know are against parental/household rules, leading to the prospect of being found 'misbehaving' by family members.
Feelings of being in the loop	Psychological	Young people feel they have information, or are part of decision-making processes, relating to particular social groups.
FOMO	Psychological	Fear of missing out - anxiety that an exciting or interesting event may currently be happening elsewhere (without you), often aroused by posts seen on social media.
Good parent-child relationships	Social	A secure attachment between parents/guardians and their children that helps promote a child's cognitive, emotional and social development.
Happiness	Psychological	A state of emotional well-being that a person experiences as a positive evaluation of one's accomplishments.
Intrusive sensory experience	Technological	Exposure to light and/or sounds associated with device use at times when young people are trying to sleep.
Learning	Behavioural<br>Educational	Use of electronic devices can expose young people to educational content, which leads to increased learning and information.
Mental distraction	Psychological	Young people use devices to either distract themselves from their concerns or seek confirmation from online activities.
Mental distraction	Psychological	Young people's use of IEDs leads to a state of mental preoccupation or diversion of attention before bedtime.
Mental health	Psychological	The absence of mental illness or poor mental wellbeing.
Modelling of device use behaviour	Behavioural	Young people observe others (any people) using (or not) their interactive electronic devices and then imitate that behaviour.
Nosiness	Behavioural	The quality of being too interested in what other people are doing and wanting to discover too much about them.
Notifications	Technological	Audible and/or mechanical alert produced by interactive electronic devices to notify the user of new/relevant content.
Offline schoolwork	Educational	Giving young people homework that does not involve the need to use interactive electronic devices.

Factor	Factor categories	Factor definition
Opportunities for sharing curriculum content	Environmental	Situations in which educational materials, resources, or information related to sleep can be disseminated or communicated to young people.
Overload of information	Psychological	The excessive or overwhelming amount of content or stimuli that young people are exposed to through device use.
Overstimulation	Physiological<br>Psychological	A state in which young people are exposed to more sensory stimuli in one or more of the body's senses than they can cope with.
Parent/guardian knowledge	Educational	Awareness of the effects of interactive electronic device use on sleep among parents/guardians.
Parental/guardian controls	Behavioural<br>Environmental<br>Social	Strategies employed by parents/guardians to limit the amount or type of interactive electronic device use among young people through non-technological means (*e.g.* time limits, device curfews *etc.*).
Parental/guardian engagement with curriculum content	Behavioural	Parents actively interact with the educational resources designed to reduce electronic device usage at bedtime among their children.
Perceived device use of peers	Psychological	The belief among young people that their peers are spending time on their interactive electronic devices, often associated with periods of time when a young person themselves is not using their device(s).
Positive offline social interactions	Social	Positive face-to-face interactions with other people.
Positive school experiences	Educational<br>Social	A phrase concerning: i) constructive relationships with peers, educators and other personnel (*e.g.* support staff); ii) sense of overall belongingness and acceptance; and iii) feelings of being supported when facing adversity.
Prioritising sleep over other activities	Behavioural	Ability to concentrate on trying to (prepare for) sleep.
Relaxation	Behavioural<br>Physiological<br>Psychological	Emotional or physical state of low tension, in which there is an absence of arousal, particularly from negative sources.
Repeat interactive electronic device use	Behavioural	Repeatedly using interactive electronic devices in similar circumstances; may or may not lead to habituation.
School restrictions on device use	Educational<br>Environmental	Strategies employed within the school to limit the amount or type of interactive electronic device use (*e.g.* banning phones in the classroom or on the premises).
Seeking peer contact	Behavioural	The behaviour of young people actively trying to engage with or interact with peers (e.g. texting, social media messaging, online gaming, or any other form of communication or interaction with friends).

Factor	Factor categories	Factor definition
Self-control	Behavioural	The ability to regulate emotions, thoughts or behaviours when faced with temptations or impulses.
Sleep promoting bedtime routines	Behavioural	A series of activities that happen each day before bed, typically carried out in the same order, and often for the purpose of producing a calming effect.
Sleep quality/duration	Outcome	Desired outcome of reducing night time device use - may encompass the following factors: duration of sleep; time taken to fall asleep; time awake during the night; ease of waking in the morning *etc.*
Stress	Psychological	State of worry or mental tension caused by a difficult situation.
Suitable internet connection	Technological	Provision of, and ability to connect devices to, a source of WiFi or data.
Teacher knowledge	Educational	Awareness among teachers of the effects of interactive electronic device use on sleep.
Technological controls	Technological	Strategies employed by parents/guardians to limit the amount or type of interactive electronic device use among young people through technological means (*e.g.* WiFi blockers or content controls *etc.*).
Time available for daytime online activities	Environmental	Space in the day to carry out online activities via interactive electronic devices in the absence of competing activities.
Time available to wind down	Behavioural	Space in the day to wind down and relax in the absence of competing activities.
Time of waking	Behavioural	The hour of day in which someone wakes up.
Time taken to fall asleep	Behavioural	The amount of time elapsed from going to bed to falling asleep (*i.e.* sleep onset).
Use of device at bedtime	Outcome	Study target behaviour: young people's use of electronic interactive devices at bedtime.
Waking at night	Behavioural	Instances during normal sleeping hours which are interrupted by periods of waking.
Worry	Psychological	The prime cognitive characteristic of worry, comprising a chain of thoughts and images, which are negatively affect-laden and relatively uncontrollable.
