# Supplementary material for: Developing a systems-based programme theory for reducing night-time interactive electronic device use and improving sleep among adolescents
Source: PLoS One. 2026 Oct 21;21(10):e0359723. doi: 10.1371/journal.pone.0359723 (PMC13686474; doi:10.1371/journal.pone.0359723)
Supplement: Supplementary file 7 [file pone.0359723.s007.pdf]

S7: Changes to CLD factors and connections

Changes between adult validated map and final CLOCK OFF causal loop diagram

Factors added to the map

Breaking parents' or guardians' rules
Desire to disconnect from content
Desire to wind down
Duration of device use
Energy expenditure
Engagement with emotion-triggering content
Exposure to educational content
Exposure to peer online activities
Mental distraction
Modelling of device use behaviour
Opportunities for sharing curriculum content
Overload of information
Parent/guardian knowledge
Parental/guardian engagement with curriculum content
Prioritising sleep over other activities
Seeking peer contact

Factors removed from the map

Awareness of time spent on device
Free time
Perceptions of control
Potential to lose games

Connections added to the map

Addictive qualities of content (+)→ Use of device at bedtime
Breaking parents' or guardians' rules (+)→ Parental/guardian controls
Breaking parents' or guardians' rules (+)→ Use of device at bedtime
Curriculum-based content (+)→ Opportunities for sharing curriculum content
Desire to disconnect from content (-)→ Use of device at bedtime
Desire to wind down (+)→ Use of device at bedtime
Duration of device use (+)→ Engagement with emotion-triggering content
Duration of device use (+)→ Overstimulation
Engagement with emotion-triggering content (+)→ Emotional dissatisfaction
Engagement with emotion-triggering content (+)→ Fear of getting caught
Enjoyment (+)→ Duration of device use
Exposure to distressing content (-)→ Relaxation
Exposure to peer online activities (+)→ Comparison of self with peers
Exposure to peer online activities (+)→ Feelings of being in the loop
Learning (+)→ Overload of information
Mental distraction (+-)→ Relaxation
Modelling of device use behaviour (+-)→ Use of device at bedtime
Opportunities for sharing curriculum content (+)→ Parental/guardian engagement with curriculum content

Overload of information (+)→ Stress
Parent/guardian knowledge (+)→ Advice from trusted adults

Parent/guardian knowledge (+)→ Education from parents/guardians
Parent/guardian knowledge (+)→ Parental/guardian controls
Parental/guardian engagement with curriculum content (+)→ Parental/guardian knowledge
Prioritising sleep over other activities (+)→ Sleep quality/duration
Seeking peer contact (+)→ Use of device at bedtime
Sleep promoting bedtime routines (-)→ Use of device at bedtime
Stress (+)→ Desire to wind down
Use of device at bedtime (+)→ Energy
Use of device at bedtime (+)→ Energy expenditure
Use of device at bedtime (+)→ Engagement with emotion-triggering content
Use of device at bedtime (+)→ Exposure to distressing content
Use of device at bedtime (+)→ Exposure to educational content
Use of device at bedtime (+)→ Exposure to peer online activities
Use of device at bedtime (+)→ Intrusive sensory experience
Use of device at bedtime (-)→ Prioritising sleep over other activities
Worry (+)→ Desire to disconnect from content
Worry (+)→ Seeking peer contact

Connections removed from the map

Awareness of time spent on device (-)→ Use of device at bedtime
Enjoyment (+)→ Fear of getting caught
Free time (+)→ Relaxation
Free time (+)→ Use of device at bedtime
Parental/guardian controls (+)→ Emotional dissatisfaction
Parental/guardian controls (+)→ Relaxation
Potential to lose games (+)→ Emotional dissatisfaction
Repeat interactive electronic device use (+)→ Relaxation
Teacher knowledge (+)→ Parental/guardian controls
Technological controls (+)→ Parental/guardian controls
Use of device at bedtime (+)→ Happiness
Use of device at bedtime (+)→ Learning
Use of device at bedtime (+)→ Worry
Worry (+)→ Use of device at bedtime
