# Supplementary material for: Developing a systems-based programme theory for reducing night-time interactive electronic device use and improving sleep among adolescents
Source: PLoS One. 2026 Oct 21;21(10):e0359723. doi: 10.1371/journal.pone.0359723 (PMC13686474; doi:10.1371/journal.pone.0359723)
Supplement: Supplementary file 8 [file pone.0359723.s008.pdf]

S8: Key feedback loops (final CLOCK OFF CLD)

Loops identified in outputs 1 and 2 containing factors related to the components of the CLOCK OFF intervention (see bold factors)

**Balancing feedback loops**	
**No.**	**Connections**
B1	Use of device at bedtime (+)→ **Parental/guardian controls** (-)→ Use of device at bedtime
B2	Use of device at bedtime (+)→ **repeat interactive electronic device use** (+)→ **parental/guardian controls** (+)→ relaxation (+)→ sleep quality/duration (+)→ energy (+)→ **positive offline social interactions** (-)→ **FOMO** (+)→ use of device at bedtime
B3	Use of device at bedtime (+)→ **repeat interactive electronic device use** (+)→ **parental/guardian controls** (+)→ **sleep promoting bedtime routines** (+)→ relaxation (+)→ sleep quality/duration (+)→ energy (+)→ **positive offline social interactions** (-)→ use of device at bedtime
B5	Use of device at bedtime (+)→ **Repeat interactive electronic device use** (+)→ **Parental/guardian controls** (-)→ Use of device at bedtime
B8	Use of device at bedtime (+)→ **Parental/guardian controls** (+)→ **Sleep promoting bedtime routines** (-)→ **Repeat electronic device use** (+)→ Use of device at bedtime
B9	Use of device at bedtime (+)→ **Parental/guardian controls** (+)→ Relaxation (+)→ Sleep quality/duration (-)→ Emotional dissatisfaction (+)→ Use of device at bedtime
B10	Use of device at bedtime (+)→ **Parental/guardian controls** (+)→ **Sleep promoting routines** (+)→ Sleep quality/duration (-)→ Emotional dissatisfaction (+)→ Use of device at bedtime
B11	Use of device at bedtime (+)→ **Repeat interactive electronic device use** (+)→ **Parental/guardian controls** (+)→ Relaxation (+)→ Sleep quality/duration (-)→ Emotional dissatisfaction (+)→ Use of device at bedtime
B12	Use of device at bedtime (+)→ **Repeat interactive electronic device use** (+)→ **Parental/guardian controls** (+)→ **Sleep promoting routines** (+)→ Sleep quality/duration (-)→ Emotional dissatisfaction (+)→ Use of device at bedtime
B13	Use of device at bedtime (+)→ **Parental/guardian controls** (+)→ **Sleep promoting routines** (+)→ Relaxation (+)→ Sleep quality/duration (-)→ Emotional dissatisfaction (+)→ Use of device at bedtime
B14	Use of device at bedtime (+)→ **Repeat interactive electronic device use** (+)→ **Parental/guardian controls** (+)→ Emotional dissatisfaction (-)→ Sleep quality/duration (-)→ Fatigue (-)→ Use of device at bedtime
B15	Use of device at bedtime (+)→ **Repeat interactive electronic device use** (+)→ **Parental/guardian controls** (+)→ **Sleep promoting routines** (+)→ Relaxation (+)→ Sleep quality and duration (+)→ Emotional dissatisfaction (+)→ Use of device at bedtime

**Reinforcing feedback loops**	
**No.**	**Connections**
R1	Use of device at bedtime (+)→ **Worry** (+)→Use of device at bedtime
R3	Use of device at bedtime (+)→ emotional dissatisfaction (-)→ happiness (+)→ sleep quality/duration (+)→ energy (+)→ **positive offline social interactions** (-)→ use of device at bedtime
R7	Use of device at bedtime (+)→ **Repeat interactive electronic device use** (+)→Use of device at bedtime

R17	Use of device at bedtime (+)→ **Repeat electronic device use** (+)→ **Parental/guardian controls** (-)→ Emotional dissatisfaction (+)→Enjoyment (+)→ Happiness (+)→ Sleep quality/duration (-)→ Fatigue (+)→ Use of device at bedtime

Loops identified in the final CLOCK OFF causal loop diagram (intervention components in bold)

**Balancing feedback loops**	
**No.**	**Connections**
B16	Use of device at bedtime (+)→ Exposure to distressing content (+)→ Worry (+)→ **Desire to disconnect** (-)→ Use of device at bedtime
B17	Use of device at bedtime (+)→ Exposure to peer online activities (+)→ **Feelings of being in the loop** (-)→ **FOMO** (+)→ Worry (+)→ **Seeking peer contact** (+)→ Use of device at bedtime
B18	Use of device at bedtime (+)→ Exposure to peer online activities (+)→ **Comparison of self with peers** (-)→ **FOMO** (+)→ Worry (+)→ **Seeking peer contact** (+)→ Use of device at bedtime

**Reinforcing feedback loops**	
**No.**	**Connections**
R18	Use of device at bedtime (+)→ **Exposure to educational content** (+)→ Learning (+)→ Overload of information(+)→ stress (+)→ Emotional dissatisfaction (+)→ Use of device at bedtime
R19	Use of device at bedtime (+)→ Exposure to distressing content (+)→ Worry (+)→ **Seeking peer contact** (+)→ Use of device at bedtime
R20	Use of device at bedtime (+)→ Exposure to peer online activities (+)→ **Feelings of being in the loop** (-)→ **FOMO** (+)→ Worry (+)→ **Desire to disconnect** (-)→ Use of device at bedtime
R21	Use of device at bedtime (+)→ Exposure to peer online activities (+)→ **Comparison of self with peers** (-)→ **FOMO** (+)→ Worry (+)→ **Desire to disconnect** (-)→ Use of device at bedtime
