# Supplementary material for: Developing a systems-based programme theory for reducing night-time interactive electronic device use and improving sleep among adolescents
Source: PLoS One. 2026 Oct 21;21(10):e0359723. doi: 10.1371/journal.pone.0359723 (PMC13686474; doi:10.1371/journal.pone.0359723)
Supplement: Supplementary file 9 [file pone.0359723.s009.pdf]

S9: components and feedback loops of programme theory

Factors

Factor	Type
Curriculum-based content	Input
Information material for parents	Input
Lesson materials and teaching plan for teachers	Input
Peer nomination survey	Input
Sleep and Social Media Survey	Input
Advice from trusted adults	Activity
Cognitive reframing	Activity
Diffusing sleep promoting messages	Activity
Education from parents/guardians	Activity
Engagement with emotion-triggering content	Activity
Exposure to educational content	Activity
Homework - parent involvement	Activity
Learning	Activity
Lessons on sleep & social media	Activity
Modelling of desired device use behaviour	Activity
Notifications	Activity
Ongoing support for peer supporter	Activity
Parental/guardian controls	Activity
Parental/guardian engagement with curriculum content	Activity
Peer supporter offers social support to pupils	Activity
Peer supporter training	Activity
Positive offline social interactions	Activity
Prioritising sleep over other activities	Activity
Prolonged device use	Activity
Repeat interactive electronic device use	Activity
Seeking peer contact	Activity
Technological controls	Activity
Training of teachers	Activity
Ability to function	Context, Assumption, Mechanism
Addictive qualities of content	Context, Assumption, Mechanism
Background operations of devices	Context, Assumption, Mechanism
Breaking parents' or guardians' rules	Context, Assumption, Mechanism
Connectedness of pupils	Context, Assumption, Mechanism
Endorsement for sleep promoting messages from parents	Context, Assumption, Mechanism
Energy	Context, Assumption, Mechanism
Enjoyment	Context, Assumption, Mechanism
Exposure to distressing content	Context, Assumption, Mechanism
Exposure to peer online activities	Context, Assumption, Mechanism
Fear of getting caught	Context, Assumption, Mechanism
Good parent-child relationships	Context, Assumption, Mechanism
Intrusive sensory experience	Context, Assumption, Mechanism
Nosiness	Context, Assumption, Mechanism

Opportunities for sharing curriculum content	Context, Assumption, Mechanism
Overload of information	Context, Assumption, Mechanism
Overstimulation	Context, Assumption, Mechanism
Perceived device use of peers	Context, Assumption, Mechanism
Positive school experiences	Context, Assumption, Mechanism
School restrictions on device use	Context, Assumption, Mechanism
Shift of social norms	Context, Assumption, Mechanism
Social influence of peer supporter	Context, Assumption, Mechanism
Stress	Context, Assumption, Mechanism
Time available for daytime online activities	Context, Assumption, Mechanism
Time available to wind down	Context, Assumption, Mechanism
Trainers are effective and have trust and rapport with teachers	Context, Assumption, Mechanism
Trainers have trust and rapport with peer supporters	Context, Assumption, Mechanism
Willingness of peer supporter	Context, Assumption, Mechanism
Ability to switch off	Short-term outcome
Agency	Short-term outcome
Awareness & knowledge of effect of device use at bedtime	Short-term outcome
Awareness of time spent on device	Short-term outcome
Comparison of self with peers	Short-term outcome
Desire to disconnect from content	Short-term outcome
Desire to wind down	Short-term outcome
Emotional dissatisfaction	Short-term outcome
Fatigue	Short-term outcome
Feelings of being in the loop	Short-term outcome
FOMO	Short-term outcome
Parent/guardian knowledge	Short-term outcome
Teacher knowledge	Short-term outcome
Worry	Short-term outcome
Use of device at bedtime (from 9pm)	Intermediate outcome
Sleep quality/duration	Medium-term outcome

Feedback loops

Balancing feedback loops	
**No.**	**Connections**
**Emotions and enjoyment**	
B19	Engagement with emotion triggering content (+)→ Emotional dissatisfaction (-)→ Enjoyment (+)→ Prolonged device use (+)→ Engagement with emotion triggering content
**Overstimulation**	
B20	Use of device at bedtime (+)→ Enjoyment (+)→ Prolonged device use (+)→ Overstimulation (+)→ Parental/guardian controls (-)→ Use of device at bedtime
B21	Use of device at bedtime (+)→ Enjoyment (+)→ Prolonged device use (+)→ Overstimulation (+)→ Fear of getting caught (-)→ Use of device at bedtime
**Reducing FOMO**	
B22	Use of device at bedtime (+)→ Exposure to peer online activities (+)→ Feelings of being in the loop (-)→ FOMO (+)→ Worry (+)→ Seeking peer contact (+)→ Use of device at bedtime
B23	Use of device at bedtime (+)→ Exposure to peer online activities (+)→ Comparison of self with peers (-)→ FOMO (+)→ Worry (+)→ Seeking peer contact (+)→ Use of device at bedtime
B24	Use of device at bedtime (+)→ Exposure to peer online activities (+)→ Feelings of being in the loop (-)→ FOMO (+)→ Worry (-)→ Sleep quality/duration (+)→ Ability to function (+)→ Agency (+)→ Ability to switch off (-)→ Use of device at bedtime
B25	Use of device at bedtime (+)→ Exposure to peer online activities (+)→ Comparison of self with peers (-)→ FOMO (+)→ Worry (-)→ Sleep quality/duration (+)→ Ability to function (+)→ Agency (+)→ Ability to switch off (-)→ Use of device at bedtime
B26	Use of device at bedtime (+)→ Exposure to peer online activities (+)→ Feelings of being in the loop (-)→ FOMO (+)→ Worry (-)→ Sleep quality/duration (-)→ Fatigue (-)→ Ability to function (+)→ Agency (+)→ Ability to switch off (-)→ Use of device at bedtime
B27	Use of device at bedtime (+)→ Exposure to peer online activities (+)→ Comparison of self with peers (-)→ FOMO (+)→ Worry (-)→ Sleep quality/duration (-)→ Fatigue (-)→ Ability to function (+)→ Agency (+)→ Ability to switch off (-)→ Use of device at bedtime
B28	Use of device at bedtime (-)→ Energy (+)→ Positive offline social interactions (-)→ FOMO (+)→ Worry (-)→ Sleep quality/duration (-)→ Fatigue (-)→ Ability to function (+)→ Agency (+)→ Ability to switch off (-)→ Use of device at bedtime
B29	Use of device at bedtime (+)→ Exposure to peer online activities (+)→ Feelings of being in the loop (-)→ FOMO (+)→ Worry (-)→ Sleep quality/duration (+)→ Energy (+)→ Ability to function (+)→ Agency (+)→ Ability to switch off (-)→ Use of device at bedtime
B30	Use of device at bedtime (+)→ Exposure to peer online activities (+)→ Comparison of self with peers (-)→ FOMO (+)→ Worry (-)→ Sleep quality/duration (+)→ Energy (+)→ Ability to function (+)→ Agency (+)→ Ability to switch off (-)→ Use of device at bedtime

Reinforcing feedback loops	
**No.**	**Connections**
**Stressful education**	
R22	Use of device at bedtime (+)→ Exposure to educational content (+)→ Learning (+)→ Overload of information (+)→ Stress (+)→ Desire to wind down (+)→ Use of device at bedtime
**Ability to function and regulate device use**	
R23	Use of device at bedtime (-)→ Sleep quality/duration (+)→ Ability to function (+)→ Agency (+)→ Ability to switch off (-)→ Use of device at bedtime

R24	Use of device at bedtime (-)→ Energy (+)→ Ability to function (+)→ Agency (+)→ Ability to switch off (-)→ Use of device at bedtime
R25	Use of device at bedtime (-)→ Sleep quality/duration (-)→ Fatigue (-)→ Ability to function (+)→ Agency (+)→ Ability to switch off (-)→ Use of device at bedtime
R26	Use of device at bedtime (-)→ Sleep quality/duration (+)→ Energy (+)→ Ability to function (+)→ Agency (+)→ Ability to switch off (-)→ Use of device at bedtime
R27	Use of device at bedtime (+)→ Exposure to distressing content (+)→ Worry (-)→ Sleep quality/duration (+)→ Ability to function (+)→ Agency (+)→ Ability to switch off (-)→ Use of device at bedtime
R28	Use of device at bedtime (+)→ Exposure to distressing content (+)→ Worry (-)→ Sleep quality/duration (-)→ Fatigue (-)→ Ability to function (+)→ Agency (+)→ Ability to switch off (-)→ Use of device at bedtime
R29	Use of device at bedtime (+)→ Exposure to distressing content (+)→ Worry (-)→ Sleep quality/duration (+)→ Energy (+)→ Ability to function (+)→ Agency (+)→ Ability to switch off (-)→ Use of device at bedtime
R30	Use of device at bedtime (-)→ Energy (+)→ Positive offline social interactions (-)→ FOMO (+)→ Worry (-)→ Sleep quality/duration (+)→ Ability to function (+)→ Agency (+)→ Ability to switch off (-)→ Use of device at bedtime
**FOMO driving device use**	
R31	Use of device at bedtime (+)→ Exposure to peer online activities (+)→ Comparison of self with peers (-)→ FOMO (+)→ Worry (+)→ Desire to disconnect (-)→ Use of device at bedtime
R32	Use of device at bedtime (+)→ Exposure to peer online activities (+)→ Worry (-)→ Sleep quality/duration (+)→ Energy (+)→ Positive offline social interactions (-)→ FOMO (+)→ Use of device at bedtime
R33	Use of device at bedtime (+)→ Exposure to distressing content (+)→ Worry (-)→ Sleep quality/duration (+)→ Energy (+)→ Positive school experiences (+)→ Positive offline social interactions (-)→ FOMO (+)→ Use of device at bedtime
R34	Use of device at bedtime (-)→ Energy (+)→ Positive school experiences (+)→ Positive offline social interactions (-)→ FOMO (+)→ Worry (-)→ Sleep quality/duration (+)→ Ability to function (+)→ Agency (+)→ Ability to switch off (-)→ Use of device at bedtime
