# Supplementary material for: Developing a systems-based programme theory for reducing night-time interactive electronic device use and improving sleep among adolescents
Source: PLoS One. 2026 Oct 21;21(10):e0359723. doi: 10.1371/journal.pone.0359723 (PMC13686474; doi:10.1371/journal.pone.0359723)
Supplement: Supplementary file 10 [file pone.0359723.s011.docx]

**Review ‘Developing a systems-based programme theory for reducing night-time interactive electronic device use and improving sleep among adolescents: the CLOCK OFF study’**

The article shows substantial potential and makes a valuable contribution to the emerging field of systems-based intervention development. That said, several aspects would benefit from further clarification and refinement - particularly regarding the justification of methodological choices (e.g., adult validation, use of centrality analysis), the presentation of results (e.g., strong emphasis on counts and feedback loops), and the need for deeper system-based interpretation of mechanisms. Also, it doesn’t become clear how a pre-defined traditional intervention (a school program) fits into this systems approach; whereby intervention programmes adapt over time based on system-level insights? Below, I provide section-by-section feedback.

**Introduction**

The introduction is rich in content but at times feels dense and repetitive. Several sentences reiterate similar points regarding complexity, the value of systems perspectives, and the limitations of linear ToCs. Streamlining would improve readability and help foreground the central argument.

In the first paragraph, you state that current interventions often fail to account for the complex and dynamic factors shaping adolescent behaviours. Providing one or two concrete examples of such interventions—and why they fall short—would strengthen this argument. Additionally, the rationale for focusing on adolescents could be elaborated. While the introduction clearly outlines why night-time IED use is problematic, it is less explicit about why a systems-based programme theory is specifically needed for this age group. What is already known about system dynamics influencing adolescent sleep and digital behaviours, and what gaps remain?

In paragraph three, you introduce the development of a programme theory. I recommend briefly explaining what a programme theory entails and how this concept is situated within systems dynamics literature.

The sentence *“Despite the growth of systems mapping in recent years…”* would benefit from concrete examples of prior empirical attempts to develop systems-based programme theories. Clarifying what exactly is missing (e.g., empirical exemplars, methodological guidance, insights into mechanisms) and why this gap matters for intervention development would sharpen the argument.

I miss references to comparable studies using a participatory systems approach in young adolescents, for example:

- Emke et al (2025): A participatory needs assessment to map the system dynamics of overweight related behaviours of 10-14-year-old adolescents living in an underserved neighbourhood in Amsterdam, the Netherlands
- Emke et al (2024): Applying systems thinking in youth-centred participatory action research for health promotion in an underserved neighbourhood
- De Pooter et al (2025): Monitoring and adaptation of a system dynamics approach to prevent childhood overweight and obesity: findings from the LIKE programme
- Emke et al (2024): Facilitating co-research: lessons learned from reflection forms within three participatory action research projects

It is also unclear why adult validation of the CLD was conducted. Introducing this rationale earlier in the introduction would prepare the reader for methodological decisions described later.

Line 126 introduces “subsystem” for the first time; if this is a central concept, consider defining it earlier.

**Minor note:**
Line 67: “mechanisms of change”.

**Methods**

The methods are extremely detailed and rigorous, but the level of description is at times overwhelming. Consider moving some operational elements (e.g., facilitator initials, note-taking roles, platform details) to supplementary materials.

What is the main research question of this study?

Line 134: “Building on existing evidence and theory [8], CLOCK OFF was developed by using the empirical insights presented in this paper to support the integration of components of existing sleep improvement interventions and behaviour change techniques more broadly..” This approach seems unusual when applying a systems thinking approach. Usually, programmes are developed based on insights of the system; rather than integrating already existing interventions. Can you explain this step more clearly? Also the resulting “a six-to-eight-week school-based intervention combining teacher-led lessons and trained peer supporters to promote healthy sleep habits and reduce night-time IED use” seems to have little to do with a systems approach – the end result is a traditional intervention. How does this work exactly?

The recruitment period for this study was 5 April 2022 to 31 May 2023; why was this so long? (over a year)?

What was the central question in the CLD mapping workshop? I.e., how does this relate to Crielaard et al (2025): Systems approaches in public health: beyond mapping the causes

**Step-specific questions and suggestions:**

- **Step 3:** How were feedback loops originally identified—were they derived directly from participant data, inferred by researchers, or a combination of both?
- **Step 5:** The rationale for validating the CLD with parents and teachers is unclear based on the introduction and earlier methods. Why was this additional validation needed?
- **Step 5:** Further justify the use of centrality analysis. Why identify the “10 most central” factors, and why was a submap necessary?
- **Step 6:** The term “mechanism-related factors” requires clarification. Also, given the system-based approach, it would strengthen the analysis to conduct an explicit systems analysis (e.g., Action Scales Model; identification of mechanisms underlying feedback loops).
- **Step 7:** This step includes substantial reinterpretation and re-labelling. Please clarify how the criteria for translating CLD elements into programme theory components were established.

More broadly, please justify sequential decisions such as:

- adolescent validation followed by adult validation,
- merging of datasets,
- use of network centrality analysis,
- combining maps into a final CLD.

Many steps rely on manual coding and interpretive synthesis. Please describe how reliability was supported (e.g., multiple coders, consensus discussions) and how biases were mitigated.

**Minor note:**
Line 217: “different uses of IEDs” – please clarify.

**Results**

The results begin with a brief overview, but two stated elements (“discussion of findings in relation to the literature” and “critical reflections on the participatory process”) do not appear in this section. I recommend adding these or adjusting the overview paragraph. A roadmap sentence linking back to the aims would improve structure, for example:
*“We first describe participant characteristics and CLD outputs, then present key subsystems, and finally outline the systems-based programme theory.”*

**CLD outputs:**
The descriptions of Outputs 1–3 are mainly numerical and technical. While some of this is important to illustrate complexity, the emphasis on counts (factors, loops, connections) risks overshadowing substantive insights. I suggest prioritising interpretive content over numbers. For example: What are the most important feedback loops (or mechanisms) impacting adolescent IED use and sleep?

The authors report >1000 feedback loops. This seems excessive? How can all these feedback loops be relevant and correct, in relation to the central question?

Line 332: “final CLD” – does this refer to the ‘amalgamated’ map?

Table 3: Were all CLOCK OFF mechanisms newly added, or were some also raised by adolescents or adults? Could you elaborate on this?

Line 373: Which leverage points? These are not described in this article?

In the subsystem analyses, I would expect deeper system-based interpretation. Currently, the narrative emphasises loops but does not fully unpack the mechanisms driving them. Conducting a more explicit system-based analysis would strengthen the contribution.

The structure of the results is somewhat difficult to follow. Two subsystems are described in detail early on, but five additional subsystems emerge later. Clarifying how these relate to each other would improve coherence.

The papers main result is a new programme theory; i.e., “The programme theory diagram included 72 factors and 147 feedback loops, grouped into five subsystems. These subsystems highlighted the dynamic interplay between enjoyment, overstimulation, fear of missing out (FOMO), emotional regulation, and educational stress .. “ But this result does in my opinion, not show a programme theory? How should system interventions (leverage points, targeting system levels) address these system dynamics?

**Discussion**

The Discussion clearly restates both aims and integrates empirical findings (e.g., FOMO, peer influence, digital environment) with methodological reflections.

**Findings related to intervention context:**
It is not fully clear why FOMO and peer influence are highlighted as key examples. I encourage the authors to shift emphasis from individual factors to key *mechanisms* or *feedback loops* illuminated by the CLD.

Comparing leverage points or subsystem dynamics to findings from Heemskerk et al. would strengthen the contribution.

The manuscript acknowledges that broader socio-environmental and macro-level conditions perpetuate IED use, yet these do not appear integrated into the CLD or programme theory. If these are considered important, this point should be woven into the introduction and analysed more systematically.

**Implementing participatory systems mapping with adolescents:**
This section offers useful reflection but could more clearly articulate the unique methodological insights arising from the study. The discussion of the “dialogical process” is interesting but could further examine how researcher interpretation influenced the evolving maps. Also I would expect some kind of comparison to other studies providing methodological frameworks, such as ENCOMPASS (Luna Pinzon et al., 2022; The ENCOMPASS framework: a practical guide for the evaluation of public health programmes in complex adaptive systems)

The link between mapping activities and the final CLOCK OFF programme theory could be made more concrete. For example, please provide one or two examples of specific adolescent insights that directly shaped programme components or mechanisms.

**Limitations:**
The acknowledgement of potential bias due to facilitators’ prior exposure to CLOCK OFF is important. Please elaborate on what type of bias this could introduce (e.g., privileging peer influence; emphasising familiar constructs such as FOMO). Also state whether any mitigation strategies (e.g. blinding, structured prompting) were employed.

Consider adding a limitation regarding constraints of the *online format* (e.g., reduced spontaneous interaction, screen fatigue, differential digital literacy).
