# Supplementary material for: Developing a systems-based programme theory for reducing night-time interactive electronic device use and improving sleep among adolescents
Source: PLoS One. 2026 Oct 21;21(10):e0359723. doi: 10.1371/journal.pone.0359723 (PMC13686474; doi:10.1371/journal.pone.0359723)
Supplement: Supplementary file 11 [file pone.0359723.s013.docx]

We thank the editor and the reviewers for taking time to consider our manuscript. We are grateful for the opportunity to revise and resubmit, and are confident that the detail feedback provided has resulted in a much stronger paper. We thank you also for the flexibility in timeframes for resubmitting this work. Here we provide a point-by-point response to the editor and reviewers. Note, where referring to **line numbers** (which have been provided only where feasible to do so given the extensive edits to the manuscript – elsewhere, broader paragraphs or sections are signposted to with reference to more cross-cutting edits), this relates to the ‘Revised Manuscript With Track Changes’ viewed in ‘**All Markup’**.

| **Editorial comments** | |
| --- | --- |
| **Comment** | **Response** |
| We note that the grant information you provided in the ‘Funding Information’ and ‘Financial Disclosure’ sections do not match. When you resubmit, please ensure that you provide the correct grant numbers for the awards you received for your study in the ‘Funding Information’ section. | Thank you for noticing this inconsistency. We have cross-referenced these two funding-related items to ensure we provide correct grant numbers. |
| The two reviewers have provided comprehensive comments to strengthen your manuscript, particularly around providing more information about the CLOCK-OFF study, ensuring you have justified your methodological choices and bringing a systems-based interpretation to your analysis. I encourage you to take into account their comments and I look forward to reading a revised manuscript. | We are grateful to the reviewers for providing such extensive feedback. We recognise that actioning this feedback has extended the length of the manuscript somewhat, and are open to the reviewers’ thoughts on this. However, we trust that our revised manuscript has suitably addressed their comments, and we hope that you enjoy reading our resubmission. |

| **Reviewer 1** | |
| --- | --- |
| **Comment** | **Response** |
| The article shows substantial potential and makes a valuable contribution to the emerging field of systems-based intervention development. That said, several aspects would benefit from further clarification and refinement - particularly regarding the justification of methodological choices (e.g., adult validation, use of centrality analysis), the presentation of results (e.g., strong emphasis on counts and feedback loops), and the need for deeper system-based interpretation of mechanisms. Also, it doesn’t become clear how a pre-defined traditional intervention (a school program) fits into this systems approach; whereby intervention programmes adapt over time based on system-level insights? Below, I provide section-by-section feedback. | We are pleased that the reviewer sees potential in this paper, and are grateful to the reviewer for providing such extensive feedback, which we feel has substantially improved the manuscript. We have carefully considered the feedback, and offer our responses below. Our responses include a range of edits and clarifications. As a result, our manuscript is extensively revised. |
| **Introduction**  The introduction is rich in content but at times feels dense and repetitive. Several sentences reiterate similar points regarding complexity, the value of systems perspectives, and the limitations of linear ToCs. Streamlining would improve readability and help foreground the central argument. | We agree that the original **Background** **section** contained some repetition. In response, we streamlined and restructured it to provide a clearer progression from the public health problem to the study rationale. The revised section now moves from the importance of adolescent night-time IED use and sleep, to the limitations of existing interventions, the role of complexity and programme theory, the added value of systems perspectives and participatory systems mapping, emerging systems-based programme theory approaches, current evidence gaps, and finally the study aims. These revisions more clearly foreground the manuscript's central contribution. |
| In the first paragraph, you state that current interventions often fail to account for the complex and dynamic factors shaping adolescent behaviours. Providing one or two concrete examples of such interventions—and why they fall short—would strengthen this argument. Additionally, the rationale for focusing on adolescents could be elaborated. While the introduction clearly outlines why night-time IED use is problematic, it is less explicit about why a systems-based programme theory is specifically needed for this age group. What is already known about system dynamics influencing adolescent sleep and digital behaviours, and what gaps remain? | In line with this comment, we strengthened the **Background section** in three ways. First, we expanded the discussion of existing interventions by providing examples and explaining that, although some have shown promise, evidence remains mixed and most target a limited set of behavioural determinants without accounting for the interacting influences shaping adolescent IED use and sleep. Second, we strengthened the rationale for focusing on adolescents by emphasising the developmental characteristics that make night-time IED use particularly responsive to influences across peer, family, school, and digital environments. Third, we expanded the discussion of relevant systems research, positioning our study in relation to previous work while highlighting key gaps, including the limited use of systems mapping and systems-based programme theory in adolescent sleep and IED research. |
| In paragraph three, you introduce the development of a programme theory. I recommend briefly explaining what a programme theory entails and how this concept is situated within systems dynamics literature. | We have added a definition of programme theory on **lines 92-95**. Edits to **lines 98-105** and **lines 132-135** help situate this more concretely within the ideas of systems dynamics. |
| The sentence “Despite the growth of systems mapping in recent years…” would benefit from concrete examples of prior empirical attempts to develop systems-based programme theories. Clarifying what exactly is missing (e.g., empirical exemplars, methodological guidance, insights into mechanisms) and why this gap matters for intervention development would sharpen the argument. | We thank the reviewer for this helpful suggestion. We have revised the **Background section** to distinguish more clearly between the growing use of systems mapping and the more limited development of systems-based programme theories. We now acknowledge emerging systems-based programme theory approaches, while emphasising that empirical public health applications remain scarce (**lines 120-138**). We clarify that key gaps include limited methodological guidance for translating participatory systems maps into programme theories (**lines 135-138**), few empirical examples of this process, and limited understanding of how systems mapping can be used to theorise intervention mechanisms in adolescent health. We also explain why this matters for intervention development (*i.e.* systems maps can improve understanding of complex problems but, without an explicit programme theory, provide less guidance on how interventions are expected to generate change) (**lines 161-174**). |
| I miss references to comparable studies using a participatory systems approach in young adolescents, for example: - Emke et al (2025): A participatory needs assessment to map the system dynamics of overweight related behaviours of 10-14-year-old adolescents living in an underserved neighbourhood in Amsterdam, the Netherlands - Emke et al (2024): Applying systems thinking in youth-centred participatory action research for health promotion in an underserved neighbourhood - De Pooter et al (2025): Monitoring and adaptation of a system dynamics approach to prevent childhood overweight and obesity: findings from the LIKE programme - Emke et al (2024): Facilitating co-research: lessons learned from reflection forms within three participatory action research projects | Thank you for these helpful reference sources. We have integrated these into the **Background section** as citation numbers 35-38, see **lines** **143-148**. |
| It is also unclear why adult validation of the CLD was conducted. Introducing this rationale earlier in the introduction would prepare the reader for methodological decisions described later. | As stated on **lines 182-186** our primary aim was to conduct systems mapping with a variety of key system actors, including teachers and parents (*i.e.* adults). We have added additional text on **line 187** introducing the reader to the concept of validation for all stakeholder groups. This has been foregrounded with additional rationale, which has been integrated on the preceding **lines 76-81**. |
| Line 126 introduces “subsystem” for the first time; if this is a central concept, consider defining it earlier. | We have added a brief definition of a subsystem in brackets, see **lines 156-157**. |
| Minor note: Line 67: “mechanisms of change”. | Thank you for noting this typographical error. We have corrected this accordingly. Please see **line 87**. |
| **Methods**  The methods are extremely detailed and rigorous, but the level of description is at times overwhelming. Consider moving some operational elements (e.g., facilitator initials, note-taking roles, platform details) to supplementary materials. | We appreciate the reviewer's perspective. However, we have retained much of the detail because a secondary aim of the paper was to generate methodological insights into the use of participatory systems mapping with adolescents. Given the limited reporting of practical and analytical processes in existing systems mapping studies, we sought to transparently describe how workshop data were generated, analysed, synthesised, and translated into a systems-based programme theory. We believe this level of detail enhances the paper's value as a methodological exemplar for researchers applying similar approaches. |
| What is the main research question of this study? | We have clarified the overarching RQ on **lines 194-197.** |
| Line 134: “Building on existing evidence and theory [8], CLOCK OFF was developed by using the empirical insights presented in this paper to support the integration of components of existing sleep improvement interventions and behaviour change techniques more broadly.” This approach seems unusual when applying a systems thinking approach. Usually, programmes are developed based on insights of the system; rather than integrating already existing interventions. Can you explain this step more clearly? Also the resulting “a six-to-eight-week school-based intervention combining teacher-led lessons and trained peer supporters to promote healthy sleep habits and reduce night-time IED use” seems to have little to do with a systems approach – the end result is a traditional intervention. How does this work exactly? | We have substantially amended the text on **lines 202-221**, to explain how our study aligned with established conventions in complex intervention development and a systems approach. We have clarified that CLOCK OFF was not developed by merely combining existing interventions. Instead, participatory systems mapping informed the programme theory, while existing interventions provided candidate components that were selected and adapted accordingly. We also clarify that a systems perspective does not require a whole-system intervention; rather, the mapping process identified school-based intervention entry points and informed how the intervention was theorised to interact with the wider system shaping adolescent night-time IED use and sleep. |
| The recruitment period for this study was 5 April 2022 to 31 May 2023; why was this so long? (over a year)? | Thank you for this observation. The recruitment period reflects the sequential nature of the systems mapping process rather than continuous participant recruitment throughout the study. Schools and participants were recruited at different stages to support both the CLD-building and subsequent validation workshops, with recruitment occurring in phases as the study progressed. In addition, data collection was scheduled around school timetables and other practical considerations associated with conducting research in school settings. To clarify this, we have added the following sentence to the manuscript (see **lines 238-240**): *“Participants were recruited between 5 April 2022 and 31 May 2023, reflecting the sequential recruitment for mapping and validation workshops, and the scheduling of data collection around school timetables.”*  We hope this clarifies the rationale for the recruitment period. |
| What was the central question in the CLD mapping workshop? I.e., how does this relate to Crielaard et al (2025): Systems approaches in public health: beyond mapping the causes | The workshops were guided by the overarching systems question: *what factors influence adolescents’ use of interactive electronic devices at bedtime, and how do interactions between these factors affect sleep?* This statement has been included on **lines 285-287**. |
| Step-specific questions and suggestions: • Step 3: How were feedback loops originally identified—were they derived directly from participant data, inferred by researchers, or a combination of both? • Step 5: The rationale for validating the CLD with parents and teachers is unclear based on the introduction and earlier methods. Why was this additional validation needed? • Step 5: Further justify the use of centrality analysis. Why identify the “10 most central” factors, and why was a submap necessary? • Step 6: The term “mechanism-related factors” requires clarification. Also, given the system-based approach, it would strengthen the analysis to conduct an explicit systems analysis (e.g., Action Scales Model; identification of mechanisms underlying feedback loops). • Step 7: This step includes substantial reinterpretation and re-labelling. Please clarify how the criteria for translating CLD elements into programme theory components were established. | Responses to step-specific comments:   - Step 3: We clarify that feedback loops were initially identified automatically with Kumu, based on the participant-derived data in the map, and that these were then manually reviewed and interpreted by the research team on **lines 344-346.** - Step 5: We have strengthened this rationale throughout the paper. In particular, detailed justification about the importance of understanding perspectives of actors in different parts of the system has been added to **lines 76-81** and **lines 364-368**. - Step 5: We have expanded the methodological justification for this analysis (see **lines 384-392**). We now explain that, given the size and complexity of the validated CLD, a submap was used to identify and examine influential feedback structures. We also clarify that no standard cut-offs exist for centrality measures, and that selecting ten factors was a pragmatic decision to balance interpretability with system complexity in line with previous literature. - Step 6: We have clarified the terminology of mechanism-related factors with a brief definition on **lines 415-416**. While we agree that explicit systems analysis is important when moving from systems mapping to intervention development, our intention was to understand how CLOCK OFF mechanisms interact with system dynamics and feedback structures, rather than apply an existing framework per se. We have strengthened and clarified our systems analyses throughout the **methods**, **results** and **discussion** sections of the manuscript, foregrounding a more explicit systems interpretation of feedback processes and contextual interactions. - Step 7: We have expanded Step 7 to make the classification process more transparent. See **lines 424-436**. We now describe how CLD factors were categorised into programme theory components using systems-based theory of change and programme theory principles, alongside the originally stated decision rules used to guide classification. These criteria were applied iteratively and refined through discussion within the research team. |
| More broadly, please justify sequential decisions such as: • adolescent validation followed by adult validation, • merging of datasets, • use of network centrality analysis, • combining maps into a final CLD. | We have added clarifications throughout the **Methods section** to explain the rationale for key technical and analytical decisions. Many of these points are hopefully already addressed with our previous responses. However, we draw the reviewer’s attention to the following sections in particular, in relation to the points raised:   - See **lines 353-356**, **364-368** - See **lines 328-332** - See **lines 384-392** - See **lines 328-332** and **Step 6** |
| Many steps rely on manual coding and interpretive synthesis. Please describe how reliability was supported (e.g., multiple coders, consensus discussions) and how biases were mitigated. | We have made substantial edits to the **Methods section** to add clarity to our methodological processes, including reference to consensus meetings on **line** **403**. Our original manuscript included mention of team discussions, which is now on **line 338**. Biases are primarily considered in the **limitations**. |
| Minor note: Line 217: “different uses of IEDs” – please clarify. | We have clarified this point by specifying the types of IED use identified during coding. See **lines 316-317**. |
| **Results** The results begin with a brief overview, but two stated elements (“discussion of findings in relation to the literature” and “critical reflections on the participatory process”) do not appear in this section. I recommend adding these or adjusting the overview paragraph. A roadmap sentence linking back to the aims would improve structure, for example: “We first describe participant characteristics and CLD outputs, then present key subsystems, and finally outline the systems-based programme theory.” | Thank you for this observation. We agree that the original paragraph inaccurately reflected that discussion of findings and methodological reflections were presented within this section of the manuscript. We have revised the text to provide a clearer roadmap linked to the study aims, describing more accurately what the Results section contains. Please see **lines 461-466**. |
| CLD outputs: The descriptions of Outputs 1–3 are mainly numerical and technical. While some of this is important to illustrate complexity, the emphasis on counts (factors, loops, connections) risks overshadowing substantive insights. I suggest prioritising interpretive content over numbers. For example: What are the most important feedback loops (or mechanisms) impacting adolescent IED use and sleep? | We agree that the value of systems mapping lies not only in describing structural complexity, rather also the interpretation of how system dynamics shape/are shaped by mechanisms, behaviours and outcomes. However, given our intention to demonstrate the fullness of the mapping process as an exemplar of detailed reporting, we feel it necessary to retain the structural details as presented in the original manuscript. However, we have included additional interpretative detail in relation to Outputs 1 and 2 on **lines 497-502**. Further interpretative material for Output 3 is initially situated on **lines 526-531** and **lines 545-549.** See comment below related to subsystems. |
| The authors report >1000 feedback loops. This seems excessive? How can all these feedback loops be relevant and correct, in relation to the central question? | We agree that reporting the total number of feedback loops without context could imply that all were equally important. We have clarified that the large number simply reflects the mathematically identifiable complexity of the system, with many loops representing minor variations of the same underlying processes (*e.g.* **lines 524-531**). Our analysis focused on the smaller set of feedback loops in the identified subsystems that were most relevant to understanding behaviour and informing intervention development, particularly those involving CLOCK OFF mechanisms and key system processes. |
| Line 332: “final CLD” – does this refer to the ‘amalgamated’ map? | The amalgamated map refers to the consolidation of three system maps created with adolescents, which were combined into a single output and then validated by the adolescents. The individual maps are not presented in the manuscript. The ‘final CLD’ refers to Output 3. We have adjusted the wording of **lines 476-480**, to better articulate the distinction for the reader. |
| Table 3: Were all CLOCK OFF mechanisms newly added, or were some also raised by adolescents or adults? Could you elaborate on this? | We have clarified that Step 6 primarily involved cross-referencing the participant-informed CLD with the theorised mechanisms underpinning CLOCK OFF, as most mechanisms were already represented by participant-derived factors. Only a small number of additional factors were introduced where necessary to represent key intervention processes. We have also clarified how intervention-relevant feedback loops were identified from the final CLD to inform development of the systems-based programme theory. See **lines 405-420**. |
| Line 373: Which leverage points? These are not described in this article? | We have now adjusted this text to more accurately reflect the focus of our analyses (i.e. the feedback processes). Please see **lines 536-537**. We have thoroughly checked and revised the manuscript to be consistent in our framing throughout. |
| In the subsystem analyses, I would expect deeper system-based interpretation. Currently, the narrative emphasises loops but does not fully unpack the mechanisms driving them. Conducting a more explicit system-based analysis would strengthen the contribution. | We have fully revised the narratives in our presentation of the two Output 3 subsystems (**lines 555-582** and **lines 584-610**), which now include deeper exploration of their dynamics and mechanisms impacting IED use and sleep. |
| The structure of the results is somewhat difficult to follow. Two subsystems are described in detail early on, but five additional subsystems emerge later. Clarifying how these relate to each other would improve coherence. | We acknowledge this concern. However, the two subsystems presented within Output 3 and the five subsystems presented in relation to Output 4 serve different analytical purposes. The former were identified from the final CLD and highlight intervention-relevant structures and feedback processes operating within the broader system. The latter were derived during programme theory development and represent higher-level groupings used to explain how CLOCK OFF is theorised to influence the system. We have clarified this distinction on **lines 633-638**. |
| The papers main result is a new programme theory; i.e., “The programme theory diagram included 72 factors and 147 feedback loops, grouped into five subsystems. These subsystems highlighted the dynamic interplay between enjoyment, overstimulation, fear of missing out (FOMO), emotional regulation, and educational stress …” But this result does in my opinion, not show a programme theory? How should system interventions (leverage points, targeting system levels) address these system dynamics? | We respectfully suggest that our use of the term systems-based programme theory (as defined in the **Background section**) differs from a focus on identifying leverage points or system levels alone. Our aim was to articulate how a discrete intervention (CLOCK OFF) is expected to interact with the wider system shaping adolescent night-time IED use and sleep through its inputs, activities, mechanisms, contextual influences, outcomes, and feedback processes. To clarify this distinction, we have strengthened the description of programme theory development (**Methods, Step 7**), and clarified in the **Discussion** that CLOCK OFF is a discrete, systems-informed intervention situated within a wider behavioural system. Relatedly, we also discuss the implications of this approach for whole school and broader systems interventions (see **lines 807-824**, and response to Reviewer 2 comment 19). |
| **Discussion** The Discussion clearly restates both aims and integrates empirical findings (e.g., FOMO, peer influence, digital environment) with methodological reflections. Findings related to intervention context: It is not fully clear why FOMO and peer influence are highlighted as key examples. I encourage the authors to shift emphasis from individual factors to key mechanisms or feedback loops illuminated by the CLD. | We agree that the study's contribution is better reflected through the mechanisms and feedback processes identified within the CLD than through discussion of individual factors in isolation. We therefore revised the opening of the Discussion (**lines 731–738**) to emphasise the interacting feedback processes and subsystem dynamics captured by the CLD and systems-based programme theory, including how factors such as FOMO, peer contact, social comparison, and worry operate within reinforcing and balancing feedback structures that influence adolescent bedtime IED use. We also note that these findings reflect the factors prioritised by adolescents during the participatory mapping process, which tended to emphasise more proximal (*i.e.* individual/social) rather than macro-level influences, and postulate as to why this may have been the case in the **Discussion.** |
| Comparing leverage points or subsystem dynamics to findings from Heemskerk et al. would strengthen the contribution. | We have expanded the Discussion to more explicitly compare our findings with those of Heemskerk et al. (see **lines 756-763**). As outlined in the **Background**, we positioned our study as extending previous systems mapping research by moving beyond identification of intervention opportunities to the development of a systems-based programme theory. The revised **Discussion** now clarifies how our findings build on Heemskerk et al. through examination of subsystem feedback processes and mechanisms of change. |
| The manuscript acknowledges that broader socio-environmental and macro-level conditions perpetuate IED use, yet these do not appear integrated into the CLD or programme theory. If these are considered important, this point should be woven into the introduction and analysed more systematically. | We agree that it is important to consider the wider socio-environmental/macro-level conditions that perpetuate IED use. While we have addressed the nature of the content of the CLD/systems based programme theory maps in our responses to previous comments, we have incorporated additional reflection on macro-level influences in various places in the **Discussion section**. For example, **lines 807-809**, **lines 821-824**, **lines 889-891** and **lines 933-939**. |
| Implementing participatory systems mapping with adolescents: This section offers useful reflection but could more clearly articulate the unique methodological insights arising from the study. The discussion of the “dialogical process” is interesting but could further examine how researcher interpretation influenced the evolving maps. Also I would expect some kind of comparison to other studies providing methodological frameworks, such as ENCOMPASS (Luna Pinzon et al., 2022; The ENCOMPASS framework: a practical guide for the evaluation of public health programmes in complex adaptive systems) | We strengthened the discussion section by providing additional reflection on the role of researcher interpretation in the evolution of the maps and programme theory (see **lines 845-849**). More broadly, our paper presents practical methodological reflections arising from the application of participatory systems mapping with adolescents for intervention development, rather than a formal methodological framework. Although we recognise the value of frameworks such as ENCOMPASS, this was developed to support the evaluation of public health programmes in complex adaptive systems, rather than intervention development. Therefore, we did not consider a detailed comparison with ENCOMPASS, or other frameworks to be appropriate. |
| The link between mapping activities and the final CLOCK OFF programme theory could be made more concrete. For example, please provide one or two examples of specific adolescent insights that directly shaped programme components or mechanisms. | We agree that the relationship between the participatory systems mapping activities and the final CLOCK OFF programme theory could be made more explicit. We have therefore added examples illustrating how participant-generated insights informed intervention development. See edits and additions made to **lines 764-795**. |
| Limitations: The acknowledgement of potential bias due to facilitators’ prior exposure to CLOCK OFF is important. Please elaborate on what type of bias this could introduce (e.g., privileging peer influence; emphasising familiar constructs such as FOMO). Also state whether any mitigation strategies (e.g. blinding, structured prompting) were employed. | We have expanded this limitation to clarify the nature of the potential bias (*e.g.* confirmation) arising from researchers' prior familiarity with the peer-influence approaches underpinning CLOCK OFF. We have also described several mitigation strategies employed throughout the study. See **lines 958-961**. |
| Consider adding a limitation regarding constraints of the online format (e.g., reduced spontaneous interaction, screen fatigue, differential digital literacy). | While we agree this could have been an issue, we actually see this more as a strength of the study, as the online format afforded us access to the participants, and the anonymity it gave people to give their views (as previously stated in our original manuscript, details now on **lines 910-912**). We have noted that digital literacy may have been a consideration in schools’ participant selection (see **lines 927-929**). |

| **Reviewer 2** | |
| --- | --- |
| **Comment** | **Response** |
| Thank you for the opportunity to review this interesting and innovative manuscript. I genuinely enjoyed reading it and believe it offers a valuable contribution to the field, particularly in demonstrating how CLDs and broader systems approaches can be used to validate and explain interventions within their contextual environments. Moreover, I was impressed by the rigorous process of building the CLDs. That said, I have several suggestions for improvement, including two overarching concerns. First, (in my opinion) the manuscript provides insufficient information about the CLOCK-OFF study itself. Without more detail in the introduction, aims, and methods, the precise process and purpose only became really clear to me around page 20. Second, the manuscript makes claims about adolescents’ experiences with the systems mapping process, yet no data are presented to substantiate these claims. Given this, the assertion that the study “critically examined the methodological and practical considerations of involving adolescents in systems mapping” feels too strong. Below, I outline more specific points for consideration: | We thank the reviewer for taking time to review our paper, and we are pleased that it was found to be rigorous, interesting and innovative, and with the potential to make a valuable contribution.  Regarding the reviewers two substantive concerns, we have revised the following:   1. On **lines 200-222**, we have added additional detail about how the systems mapping process was used in the development of CLOCK OFF, and give details about the discrete nature of the intervention (**lines 217-221**). This is preceded by more explicit aims, which include an outline description of CLOCK OFF on **lines 178-180**. The research gaps to which CLOCK OFF attends are now more clearly articulated in the narrative of the **Background section**. A new **Additional File 1** provides a brief overview of CLOCK OFF components. Our original manuscript already contained what is now Additional File 2, which provides information about the programmes upon which CLOCK OFF draws, and we have added additional signposting to this (*e.g.* on **line 515**). Further, we included what is now Additional File 4, which highlights the key inputs and activities of the CLOCK OFF intervention. Unfortunately at this time it is not possible to cite a ‘sister paper’ that also contains the intervention detail, as it remains under review. We have however mentioned the forthcoming paper, as in our original manuscript. We hope these adjustments provide sufficient detail about the study itself. 2. We accept that the original presentation of ‘findings’ related to the considerations of mapping with adolescents was too definitive in its claims, and it implied that we undertook more empirical research activities to directly observe and assess adolescents’ experiences of mapping. This was not the case, and therefore we have tempered the tone, presenting our reflections and personal research experiences. These adjustments are primarily found on **lines 825-827** (including an adjusted subheading for clarity), **line 867**, **lines 877-883**, and **lines 897-906**. In line with specific reviewer comments below, we have strengthened proposed directions for future research by advocating for more explicit assessment of systems mapping with adolescent groups (**lines 945-947**). |
| 1. Title: Consider removing “CLOCK-OFF” from the title, as it may mislead readers about the manuscript’s primary focus. | We have removed the subtitle to avoid unnecessary confusion (see **line 4**)**.** |
| 2. Background: Although CLOCK-OFF is briefly introduced, more contextual information is needed. Even if the intervention is described elsewhere, a short overview of its components would help readers understand the mapping process. It also remains unclear whether CLOCK-OFF is fully developed, still a prototype, or how it was originally created. | We have created a new **Additional File 1** (signposted on **line 205**) to give a brief overview of CLOCK OFF’s components parts. This is necessarily brief to protect the intellectual integrity of our forthcoming article. We have also clarified the developmental status of CLOCK OFF at the time at which the work reported in this paper was completed (see **lines 180-183**). Since the time of the work being reported here, the CLOCK OFF intervention has been manualised and piloted, findings from which are reported in our forthcoming publication, as noted on **lines 221-222** and **lines 254-255**, and in our response to the Reviewer 2’s overarching comment above. |
| 3. Study aims: The aims would benefit from being stated as clearly as in the discussion, where the authors note that they “adopted a systems perspective to ensure the development of an intervention that was theoretically informed, responded to the contextual causes of the problem, and was contextually sensitive.” This clarity is missing in the aims section. | We have significantly restructured the presentation of the study aims in line with feedback from both reviewers. We have also included our guiding research question. See **lines 178-197**. |
| 4. Materials and Methods (line 139f): It is unclear whether CLOCK-OFF was developed participatorily. Moreover, at this point, it reads as though CLOCK-OFF is a finished intervention, yet later sections imply that the current work could inform its refinement or validation. | We have amended the **Methods section** to add more detail about the development of CLOCK OFF, not withstanding that some of this detail will be provided in our second forthcoming manuscript as signposted. On lines. Our previous responses also highlight the developmental nature of CLOCK OFF at the time of the work being reported (see **lines 180-183**). We clarify on **lines 203-204** that the intervention development work had several inputs, including participatory components. Participatory systems mapping contributed to identifying influences on adolescent IED use, mechanism of change and intervention entry points. This learning was integrated iteratively through the processes of intervention development and refinement. |
| 5. Study design (line 143f): Consider referring readers to the more detailed description provided later in the manuscript. | The study design section of the Methods has been considerably altered in line with reviewers’ feedback. Therefore, it was unclear whether this comment was still relevant to address. We have added additional signposting to **lines 268-269**, and we trust this is a suitable adjustment. |
| 6. Recruitment (line 157): School contacts identified adolescents for participation. Please discuss how this recruitment strategy may have introduced bias and consider adding this to the limitations. | We have added a brief reflection on the potential risk of bias through recruitment in the study limitations on **lines 929-931**. |
| 7. Line 165: The manuscript states that “additional details about the intervention development workshops will be reported elsewhere,” but it remains unclear how these processes relate. Is this not part of a single development trajectory? If the current work contributes to testing or validating CLOCK-OFF, it may belong within the development phase and should be described accordingly. | In responding to both sets of reviewer comments, we have attempted to strengthen the positioning of our manuscript as reporting on one key aspect of a wider intervention development and refinement process. Specifically, this paper describes the development of an initial systems-based programme theory, which was used to help identify potential intervention components and theorise the mechanisms and systems dynamics that may influence outcomes. Edits to the **CLOCK OFF**, **study design** and **data collection** sections make this relationship explicit, and we signpost the reader to our other forthcoming publication, which provides further detail about intervention integration, refinement and piloting. Therefore, we agree that this is part of a single development trajectory, while emphasising processes were iterative rather than linear. |
| 8. Line 172: Mental health is highlighted here, while in the introduction also educational outcomes and other health outcomes are mentioned. Clarification would be helpful. | Thank you for noticing this inconsistency. We have amended the opening paragraph to reflect the focus on mental health in particular, as being a key issue associated with night-time IED use among adolescents. See **line 59**. |
| 9. Line 250f: The validation process for the CLD is rigorous, but why was the adult CLD not re checked with adolescents? Given that elements were added or removed, revalidation with adolescents would align with participatory principles. | We have added clarification to the limitations section on **lines 942-943**, citing practical issues. |
| 10. Line 281, Step 4: A brief example would help clarify how the CLD was mapped onto CLOCK-OFF. Again, this reinforces the need for a clearer description of the intervention earlier in the manuscript. | Please see earlier responses about additional information relating to CLOCK OFF components. We have added some additional detail in **Step 6** that helps clarify the analytical relationship between the developed CLD and CLOCK OFF inputs, activities and proposed mechanisms. |
| 11. Lines 319–321: This could potentially be removed. | The manuscript has since been substantially revised, and the text originally appearing at these lines has been reorganised and rewritten as part of broader revisions to the Results section. We therefore believe this concern has been addressed through revision of the surrounding text. |
| 12. Line 323: The participant age could be said to be broad, given the developmental phase (and especially regarding sleep health?) Did developmental differences (e.g., puberty) influence participation or the mapping process? | Participants were recruited from the target age group for the CLOCK OFF intervention (11-14 years). While we recognise that developmental differences exist within this age range, the study was not designed to examine variation according to age or pubertal status, nor were data collected that would permit such analyses. References to developmental appropriateness in the manuscript relate to the design and delivery of participatory activities and intervention content, rather than to the examination of developmental differences between participants. Consequently, we have not added speculative interpretation regarding the influence of puberty or developmental stage on the resulting maps. |
| 13. Line 329f: This section confused me a little, as I understood the main output to be a single final CLD? | We agree that the progression from the interim mapping outputs to the final CLD and systems-based programme theory could have been clearer. We hope that in making edits in response to Reviewer 1 (see **lines 478-482**), we have also addressed this comment. To clarify, Outputs 3 and 4 illustrate distinct processes, each contributing to intervention development. While the finalised CLD (Output 3) represents the principal systems map produced by the study, the primary study output is the systems-based programme theory diagram (Output 4), which was derived from the final CLD. Outputs 1 and 2 were retained to illustrate how the representations of the system evolved during the study. |
| 14. Table 3: A more thorough description of CLOCK-OFF is needed to interpret this table meaningfully. | We agree that understanding CLOCK OFF is important for interpreting Table 3. In response to this and related comments from both reviewers, we have substantially expanded the description of CLOCK OFF throughout the manuscript, particularly in the Background, Study Aims and Methods sections. We have added **Additional File 1.** The revised manuscript now provides additional detail regarding the intervention's developmental status, constituent components, theoretical basis, delivery model, and the role of systems mapping in informing intervention development and programme theory generation. We have also added a signpost adjacent to Table 3 directing readers to what are now Additional Files 2 and 4, which provides further detail regarding the intervention components that informed CLOCK OFF. |
| 15. Line 369 onwards: From this point, the mapping process becomes clearer. It appears the aim is to predict or validate an intervention’s effectiveness within context. Could this approach be applied to other healthy sleep interventions? You touch on context dependency later, but this could be expanded. | To clarify, the purpose of the systems-based programme theory was not to predict or validate intervention effectiveness, but rather to make explicit how CLOCK OFF is expected to operate within the wider system influencing adolescent night-time IED use and sleep. In line with our definition of programme theory (see **lines 94-97**), the map seeks to represent intervention inputs, activities, mechanisms, contextual influences and outcomes, and the dynamic interactions between them. We agree that the broader applicability of this approach is an important consideration and have added a brief reflection to the Discussion noting that the methodological approach may have relevance beyond CLOCK OFF, while recognising that programme theories are necessarily context-dependent (see **lines 728-732**). |
| 16. Line 454f: This suggests that CLOCK-OFF is not yet a fully developed intervention (“…should be important considerations in CLOCK-OFF”). Do you plan to integrate these considerations? Is CLOCK-OFF currently a prototype to be refined? Could additional CLD processes with other adolescent groups further strengthen validation? | We agree that the original manuscript did not sufficiently distinguish between intervention development and evaluation. The revised manuscript now makes clear that CLOCK OFF was in the intervention development phase at the time of the study, and that the systems mapping process was used to identify intervention targets, mechanisms of change and implementation considerations that informed ongoing intervention refinement. While additional participatory mapping with other adolescent groups may further extend understanding of the system, formal validation beyond the scope of the current study would require future research, which we propose in the limitations (see **lines 937-939**). |
| 17. Line 495f: This sentence may need revision. | Thank you for noting the clarity of this sentence, which we have now improved. See **lines 709-711**. |
| 18. Discussion (line 509f): The insights about using these methods with adolescents are not grounded in data. This relates to my earlier concern that claims about adolescents’ experiences are not supported by data. | We agree that the study did not collect formal evaluation data regarding adolescents' experiences of participatory systems mapping and have therefore been careful not to present the methodological reflections as empirical findings about participant experiences. In response to this and related comments, we have revised the Discussion to emphasise that these observations represent practical and methodological reflections arising from implementation of the approach (**lines 827-908**), rather than direct evidence regarding adolescents' experiences. We have also expanded the Limitations section to acknowledge the absence of participant evaluation data and recommend that future studies collect such data through approaches such as reflection forms, interviews, focus groups or other workshop evaluation activities (**lines 947-949**). See also our response to overarching reviewer comment above. |
| 19. Line 563: When discussing a whole school approach, consider referencing literature on challenges related to teacher involvement, as this is a well documented issue. | We agree that it would be beneficial to have noted some of the challenges associated with whole school approaches. Therefore, we have added detail on **lines 817-819**, explaining the importance of influences of workload, autonomy, leadership and culture. |
| 20. Line 570 onwards (implementing PSM with adolescents): This section lacks depth and supporting data. Interviews, focus groups, or researcher reflections could have provided insight into adolescents’ experiences. Without such data, the considerations remain basic and do not substantially advance existing co creation literature. The manuscript may need to temper claims about filling this gap. | Please see our responses to the reviewer’s overarching comment, and comment 18 above. We have reframed **lines 827-908** to significantly temper the claims made, with a focus on our researcher reflections, as opposed to giving an impression of empirical insights. |
| 21. Limitations (line 645): Consider discussing the (arguably) broad age range and uneven sex/gender distribution. | Participants were recruited from the intended target population for CLOCK OFF (11-14 years). The study was not designed to examine age-related or developmental differences in perceptions of IED use and sleep, and data were not collected that would permit such analyses. We therefore do not believe the age range constitutes a substantive limitation in relation to the study aims. We have made a recommendation to explore the impacts of sex and gender on **lines** **931-933**. |
| 22. Line 647: Including a fourth school could also be framed as a strength, as it broadens contextual diversity. | We agree with this assessment, and have added a clause to this effect on **line 354**. |
| 23. Line 653: When suggesting that future studies should prioritise certain aspects, please specify how. E.g., through reflection forms, interviews, or focus groups. | We agree with this suggestion, and have added a clause to this effect on lines **949-951**. |
| 24. Line 659 onwards: This section warrants deeper discussion. Many sleep interventions share similar components; how might other interventions fit within this framework? What strategies could reduce bias? | We agree that the broader applicability of the approach warrants consideration. We have clarified that, while the systems-based programme theory developed in this study is specific to CLOCK OFF and its implementation context, the participatory systems mapping approach may have wider relevance for the development of other sleep- and health-related interventions (**lines 728-732**). We have also expanded the limitations section (**line 930** and **lines 963-964**) to discuss potential sources of bias and the mitigation strategies employed, including participant-led mapping, stakeholder validation and iterative review of existing evidence. |
| 25. General remark: Could the CLD process itself function as an intervention? A brief reflection on this possibility may be valuable. | Yes, systems mapping may also function as an intervention in and of itself, yet this is seldom reflected on in published studies. We have added a short reflection to this effect on **lines 899-903**. |
| 26. Supplements: Figure 2 requires more explanation, particularly regarding the four headings in the corners. | Details of these questions are already provided in Additional File 3 (the workshop guide), but we have added additional clarity in the main body of the text as to how these questions were used as prompts for factor elicitation. See **lines 297-299**. |
