# Supplementary material for: Developing a systems-based programme theory for reducing night-time interactive electronic device use and improving sleep among adolescents
Source: PLoS One. 2026 Oct 21;21(10):e0359723. doi: 10.1371/journal.pone.0359723 (PMC13686474; doi:10.1371/journal.pone.0359723)
Supplement: Peer review history

# Peer review: Decision Letter 0

**DOI:** 10.1371/journal.pone.0359723.r001

**Authors:** Elizabeth McGill

19 Jan 2026

Dear Dr. Rigby,

Thank you for submitting your manuscript to PLOS ONE. After careful consideration, we feel that it has merit but does not fully meet PLOS ONE’s publication criteria as it currently stands. Therefore, we invite you to submit a revised version of the manuscript that addresses the points raised during the review process.

Please submit your revised manuscript by Mar 05 2026 11:59PM. If you will need more time than this to complete your revisions, please reply to this message or contact the journal office at plosone@plos.org. When you're ready to submit your revision, log on to https://www.editorialmanager.com/pone/ and select the 'Submissions Needing Revision' folder to locate your manuscript file.

- A letter that responds to each point raised by the academic editor and reviewer(s). You should upload this letter as a separate file labeled 'Response to Reviewers'.
- A marked-up copy of your manuscript that highlights changes made to the original version. You should upload this as a separate file labeled 'Revised Manuscript with Track Changes'.
- An unmarked version of your revised paper without tracked changes. You should upload this as a separate file labeled 'Manuscript'.

If you would like to make changes to your financial disclosure, please include your updated statement in your cover letter. Guidelines for resubmitting your figure files are available below the reviewer comments at the end of this letter.

If applicable, we recommend that you deposit your laboratory protocols in protocols.io to enhance the reproducibility of your results. Protocols.io assigns your protocol its own identifier (DOI) so that it can be cited independently in the future. For instructions see: https://journals.plos.org/plosone/s/submission-guidelines#loc-laboratory-protocols. Additionally, PLOS ONE offers an option for publishing peer-reviewed Lab Protocol articles, which describe protocols hosted on protocols.io. Read more information on sharing protocols at https://plos.org/protocols?utm_medium=editorial-email&utm_source=authorletters&utm_campaign=protocols.

We look forward to receiving your revised manuscript.

Kind regards,

Elizabeth McGill

Academic Editor

PLOS One

Journal Requirements:

When submitting your revision, we need you to address these additional requirements.

1.Please ensure that your manuscript meets PLOS ONE’s style requirements, including those for file naming. The PLOS ONE style templates can be found at

https://journals.plos.org/plosone/s/file?id=wjVg/PLOSOne_formatting_sample_main_body.pdf and

https://journals.plos.org/plosone/s/file?id=ba62/PLOSOne_formatting_sample_title_authors_affiliations.pdf

2. Please note that PLOS One has specific guidelines on code sharing for submissions in which author-generated code underpins the findings in the manuscript. In these cases, we expect all author-generated code to be made available without restrictions upon publication of the work. Please review our guidelines at https://journals.plos.org/plosone/s/materials-and-software-sharing#loc-sharing-code and ensure that your code is shared in a way that follows best practice and facilitates reproducibility and reuse.

3. Please note that funding information should not appear in any section or other areas of your manuscript. We will only publish funding information present in the Funding Statement section of the online submission form. Please remove any funding-related text from the manuscript.

4. We note that the grant information you provided in the ‘Funding Information’ and ‘Financial Disclosure’ sections do not match.

When you resubmit, please ensure that you provide the correct grant numbers for the awards you received for your study in the ‘Funding Information’ section.

5. Your ethics statement should only appear in the Methods section of your manuscript. If your ethics statement is written in any section besides the Methods, please delete it from any other section.

6. Please include captions for your Supporting Information files at the end of your manuscript, and update any in-text citations to match accordingly. Please see our Supporting Information guidelines for more information: http://journals.plos.org/plosone/s/supporting-information.

7. If the reviewer comments include a recommendation to cite specific previously published works, please review and evaluate these publications to determine whether they are relevant and should be cited. There is no requirement to cite these works unless the editor has indicated otherwise.

Additional Editor Comments:

The two reviewers have provided comprehensive comments to strengthen your manuscript, particularly around providing more information about the CLOCK-OFF study, ensuring you have justified your methodological choices and bringing a systems-based interpretation to your analysis. I encourage you to take into account their comments and I look forward to reading a revised manuscript.

[Note: HTML markup is below. Please do not edit.]

Reviewers' comments:

Reviewer’s Responses to Questions

**Comments to the Author**

1. Is the manuscript technically sound, and do the data support the conclusions?

Reviewer #1: Partly

Reviewer #2: Partly

**********

2. Has the statistical analysis been performed appropriately and rigorously?

Reviewer #1: No

Reviewer #2: N/A

**********

3. Have the authors made all data underlying the findings in their manuscript fully available?

The PLOS Data policy

Reviewer #1: Yes

Reviewer #2: No

**********

4. Is the manuscript presented in an intelligible fashion and written in standard English?

Reviewer #1: Yes

Reviewer #2: Yes

**********

Reviewer #1: Review ‘Developing a systems-based programme theory for reducing night-time interactive electronic device use and improving sleep among adolescents: the CLOCK OFF study’

The article shows substantial potential and makes a valuable contribution to the emerging field of systems-based intervention development. That said, several aspects would benefit from further clarification and refinement - particularly regarding the justification of methodological choices (e.g., adult validation, use of centrality analysis), the presentation of results (e.g., strong emphasis on counts and feedback loops), and the need for deeper system-based interpretation of mechanisms. Also, it doesn’t become clear how a pre-defined traditional intervention (a school program) fits into this systems approach; whereby intervention programmes adapt over time based on system-level insights? Below, I provide section-by-section feedback.

Introduction

The introduction is rich in content but at times feels dense and repetitive. Several sentences reiterate similar points regarding complexity, the value of systems perspectives, and the limitations of linear ToCs. Streamlining would improve readability and help foreground the central argument.

In the first paragraph, you state that current interventions often fail to account for the complex and dynamic factors shaping adolescent behaviours. Providing one or two concrete examples of such interventions—and why they fall short—would strengthen this argument. Additionally, the rationale for focusing on adolescents could be elaborated. While the introduction clearly outlines why night-time IED use is problematic, it is less explicit about why a systems-based programme theory is specifically needed for this age group. What is already known about system dynamics influencing adolescent sleep and digital behaviours, and what gaps remain?

In paragraph three, you introduce the development of a programme theory. I recommend briefly explaining what a programme theory entails and how this concept is situated within systems dynamics literature.

The sentence “Despite the growth of systems mapping in recent years…” would benefit from concrete examples of prior empirical attempts to develop systems-based programme theories. Clarifying what exactly is missing (e.g., empirical exemplars, methodological guidance, insights into mechanisms) and why this gap matters for intervention development would sharpen the argument.

I miss references to comparable studies using a participatory systems approach in young adolescents, for example:

- Emke et al (2025): A participatory needs assessment to map the system dynamics of overweight related behaviours of 10-14-year-old adolescents living in an underserved neighbourhood in Amsterdam, the Netherlands

- Emke et al (2024): Applying systems thinking in youth-centred participatory action research for health promotion in an underserved neighbourhood

- De Pooter et al (2025): Monitoring and adaptation of a system dynamics approach to prevent childhood overweight and obesity: findings from the LIKE programme

- Emke et al (2024): Facilitating co-research: lessons learned from reflection forms within three participatory action research projects

It is also unclear why adult validation of the CLD was conducted. Introducing this rationale earlier in the introduction would prepare the reader for methodological decisions described later.

Line 126 introduces “subsystem” for the first time; if this is a central concept, consider defining it earlier.

Minor note:

Line 67: “mechanisms of change”.

Methods

The methods are extremely detailed and rigorous, but the level of description is at times overwhelming. Consider moving some operational elements (e.g., facilitator initials, note-taking roles, platform details) to supplementary materials.

What is the main research question of this study?

Line 134: “Building on existing evidence and theory [8], CLOCK OFF was developed by using the empirical insights presented in this paper to support the integration of components of existing sleep improvement interventions and behaviour change techniques more broadly..” This approach seems unusual when applying a systems thinking approach. Usually, programmes are developed based on insights of the system; rather than integrating already existing interventions. Can you explain this step more clearly? Also the resulting “a six-to-eight-week school-based intervention combining teacher-led lessons and trained peer supporters to promote healthy sleep habits and reduce night-time IED use” seems to have little to do with a systems approach – the end result is a traditional intervention. How does this work exactly?

The recruitment period for this study was 5 April 2022 to 31 May 2023; why was this so long? (over a year)?

What was the central question in the CLD mapping workshop? I.e., how does this relate to Crielaard et al (2025): Systems approaches in public health: beyond mapping the causes

Step-specific questions and suggestions:

• Step 3: How were feedback loops originally identified—were they derived directly from participant data, inferred by researchers, or a combination of both?

• Step 5: The rationale for validating the CLD with parents and teachers is unclear based on the introduction and earlier methods. Why was this additional validation needed?

• Step 5: Further justify the use of centrality analysis. Why identify the “10 most central” factors, and why was a submap necessary?

• Step 6: The term “mechanism-related factors” requires clarification. Also, given the system-based approach, it would strengthen the analysis to conduct an explicit systems analysis (e.g., Action Scales Model; identification of mechanisms underlying feedback loops).

• Step 7: This step includes substantial reinterpretation and re-labelling. Please clarify how the criteria for translating CLD elements into programme theory components were established.

More broadly, please justify sequential decisions such as:

• adolescent validation followed by adult validation,

• merging of datasets,

• use of network centrality analysis,

• combining maps into a final CLD.

Many steps rely on manual coding and interpretive synthesis. Please describe how reliability was supported (e.g., multiple coders, consensus discussions) and how biases were mitigated.

Minor note:

Line 217: “different uses of IEDs” – please clarify.

Results

The results begin with a brief overview, but two stated elements (“discussion of findings in relation to the literature” and “critical reflections on the participatory process”) do not appear in this section. I recommend adding these or adjusting the overview paragraph. A roadmap sentence linking back to the aims would improve structure, for example:

“We first describe participant characteristics and CLD outputs, then present key subsystems, and finally outline the systems-based programme theory.”

CLD outputs:

The descriptions of Outputs 1–3 are mainly numerical and technical. While some of this is important to illustrate complexity, the emphasis on counts (factors, loops, connections) risks overshadowing substantive insights. I suggest prioritising interpretive content over numbers. For example: What are the most important feedback loops (or mechanisms) impacting adolescent IED use and sleep?

The authors report >1000 feedback loops. This seems excessive? How can all these feedback loops be relevant and correct, in relation to the central question?

Line 332: “final CLD” – does this refer to the ‘amalgamated’ map?

Table 3: Were all CLOCK OFF mechanisms newly added, or were some also raised by adolescents or adults? Could you elaborate on this?

Line 373: Which leverage points? These are not described in this article?

In the subsystem analyses, I would expect deeper system-based interpretation. Currently, the narrative emphasises loops but does not fully unpack the mechanisms driving them. Conducting a more explicit system-based analysis would strengthen the contribution.

The structure of the results is somewhat difficult to follow. Two subsystems are described in detail early on, but five additional subsystems emerge later. Clarifying how these relate to each other would improve coherence.

The papers main result is a new programme theory; i.e., “The programme theory diagram included 72 factors and 147 feedback loops, grouped into five subsystems. These subsystems highlighted the dynamic interplay between enjoyment, overstimulation, fear of missing out (FOMO), emotional regulation, and educational stress .. “ But this result does in my opinion, not show a programme theory? How should system interventions (leverage points, targeting system levels) address these system dynamics?

Discussion

The Discussion clearly restates both aims and integrates empirical findings (e.g., FOMO, peer influence, digital environment) with methodological reflections.

Findings related to intervention context:

It is not fully clear why FOMO and peer influence are highlighted as key examples. I encourage the authors to shift emphasis from individual factors to key mechanisms or feedback loops illuminated by the CLD.

Comparing leverage points or subsystem dynamics to findings from Heemskerk et al. would strengthen the contribution.

The manuscript acknowledges that broader socio-environmental and macro-level conditions perpetuate IED use, yet these do not appear integrated into the CLD or programme theory. If these are considered important, this point should be woven into the introduction and analysed more systematically.

Implementing participatory systems mapping with adolescents:

This section offers useful reflection but could more clearly articulate the unique methodological insights arising from the study. The discussion of the “dialogical process” is interesting but could further examine how researcher interpretation influenced the evolving maps. Also I would expect some kind of comparison to other studies providing methodological frameworks, such as ENCOMPASS (Luna Pinzon et al., 2022; The ENCOMPASS framework: a practical guide for the evaluation of public health programmes in complex adaptive systems)

The link between mapping activities and the final CLOCK OFF programme theory could be made more concrete. For example, please provide one or two examples of specific adolescent insights that directly shaped programme components or mechanisms.

Limitations:

The acknowledgement of potential bias due to facilitators’ prior exposure to CLOCK OFF is important. Please elaborate on what type of bias this could introduce (e.g., privileging peer influence; emphasising familiar constructs such as FOMO). Also state whether any mitigation strategies (e.g. blinding, structured prompting) were employed.

Consider adding a limitation regarding constraints of the online format (e.g., reduced spontaneous interaction, screen fatigue, differential digital literacy).

Reviewer #2: Thank you for the opportunity to review this interesting and innovative manuscript. I genuinely enjoyed reading it and believe it offers a valuable contribution to the field, particularly in demonstrating how CLDs and broader systems approaches can be used to validate and explain interventions within their contextual environments. Moreover, I was impressed by the rigorous process of building the CLDs.

That said, I have several suggestions for improvement, including two overarching concerns. First, (in my opinion) the manuscript provides insufficient information about the CLOCK-OFF study itself. Without more detail in the introduction, aims, and methods, the precise process and purpose only became really clear to me around page 20. Second, the manuscript makes claims about adolescents’ experiences with the systems mapping process, yet no data are presented to substantiate these claims. Given this, the assertion that the study “critically examined the methodological and practical considerations of involving adolescents in systems mapping” feels too strong.

Below, I outline more specific points for consideration:

1. Title: Consider removing “CLOCK-OFF” from the title, as it may mislead readers about the manuscript’s primary focus.

2. Background: Although CLOCK-OFF is briefly introduced, more contextual information is needed. Even if the intervention is described elsewhere, a short overview of its components would help readers understand the mapping process. It also remains unclear whether CLOCK-OFF is fully developed, still a prototype, or how it was originally created.

3. Study aims: The aims would benefit from being stated as clearly as in the discussion, where the authors note that they “adopted a systems perspective to ensure the development of an intervention that was theoretically informed, responded to the contextual causes of the problem, and was contextually sensitive.” This clarity is missing in the aims section.

4. Materials and Methods (line 139f): It is unclear whether CLOCK-OFF was developed participatorily. Moreover, at this point, it reads as though CLOCK-OFF is a finished intervention, yet later sections imply that the current work could inform its refinement or validation.

5. Study design (line 143f): Consider referring readers to the more detailed description provided later in the manuscript.

6. Recruitment (line 157): School contacts identified adolescents for participation. Please discuss how this recruitment strategy may have introduced bias and consider adding this to the limitations.

7. Line 165: The manuscript states that “additional details about the intervention development workshops will be reported elsewhere,” but it remains unclear how these processes relate. Is this not part of a single development trajectory? If the current work contributes to testing or validating CLOCK-OFF, it may belong within the development phase and should be described accordingly.

8. Line 172: Mental health is highlighted here, while in the introduction also educational outcomes and other health outcomes are mentioned. Clarification would be helpful.

9. Line 250f: The validation process for the CLD is rigorous, but why was the adult CLD not re checked with adolescents? Given that elements were added or removed, revalidation with adolescents would align with participatory principles.

10. Line 281, Step 4: A brief example would help clarify how the CLD was mapped onto CLOCK-OFF. Again, this reinforces the need for a clearer description of the intervention earlier in the manuscript.

11. Lines 319–321: This could potentially be removed.

12. Line 323: The participant age could be said to be broad, given the developmental phase (and especially regarding sleep health?) Did developmental differences (e.g., puberty) influence participation or the mapping process?

13. Line 329f: This section confused me a little, as I understood the main output to be a single final CLD?

14. Table 3: A more thorough description of CLOCK-OFF is needed to interpret this table meaningfully.

15. Line 369 onwards: From this point, the mapping process becomes clearer. It appears the aim is to predict or validate an intervention’s effectiveness within context. Could this approach be applied to other healthy sleep interventions? You touch on context dependency later, but this could be expanded.

16. Line 454f: This suggests that CLOCK-OFF is not yet a fully developed intervention (“…should be important considerations in CLOCK-OFF”). Do you plan to integrate these considerations? Is CLOCK-OFF currently a prototype to be refined? Could additional CLD processes with other adolescent groups further strengthen validation?

17. Line 495f: This sentence may need revision.

18. Discussion (line 509f): The insights about using these methods with adolescents are not grounded in data. This relates to my earlier concern that claims about adolescents’ experiences are not supported by data.

19. Line 563: When discussing a whole school approach, consider referencing literature on challenges related to teacher involvement, as this is a well documented issue.

20. Line 570 onwards (implementing PSM with adolescents): This section lacks depth and supporting data. Interviews, focus groups, or researcher reflections could have provided insight into adolescents’ experiences. Without such data, the considerations remain basic and do not substantially advance existing co creation literature. The manuscript may need to temper claims about filling this gap.

21. Limitations (line 645): Consider discussing the (arguably) broad age range and uneven sex/gender distribution.

22. Line 647: Including a fourth school could also be framed as a strength, as it broadens contextual diversity.

23. Line 653: When suggesting that future studies should prioritise certain aspects, please specify how. E.g., through reflection forms, interviews, or focus groups.

24. Line 659 onwards: This section warrants deeper discussion. Many sleep interventions share similar components; how might other interventions fit within this framework? What strategies could reduce bias?

25. General remark: Could the CLD process itself function as an intervention? A brief reflection on this possibility may be valuable.

26. Supplements: Figure 2 requires more explanation, particularly regarding the four headings in the corners.

**********

what does this mean?). If published, this will include your full peer review and any attached files.

If you choose “no”, your identity will remain anonymous but your review may still be made public.

**Do you want your identity to be public for this peer review?**  For information about this choice, including consent withdrawal, please see our Privacy Policy

Reviewer #1: No

Reviewer #2: No

**********

[NOTE: If reviewer comments were submitted as an attachment file, they will be attached to this email and accessible via the submission site. Please log into your account, locate the manuscript record, and check for the action link "View Attachments". If this link does not appear, there are no attachment files.]

To ensure your figures meet our technical requirements, please review our figure guidelines: https://journals.plos.org/plosone/s/figures

You may also use PLOS’s free figure tool, NAAS, to help you prepare publication quality figures: https://journals.plos.org/plosone/s/figures#loc-tools-for-figure-preparation.

NAAS will assess whether your figures meet our technical requirements by comparing each figure against our figure specifications.

Attachment Submitted filename: Review_2.docx

# Author comment: Author response to Decision Letter 1

**DOI:** 10.1371/journal.pone.0359723.r002

20 Jul 2026

We thank the editor and the reviewers for taking time to consider our manuscript. We are grateful for the opportunity to revise and resubmit, and are confident that the detail feedback provided has resulted in a much stronger paper. We thank you also for the flexibility in timeframes for resubmitting this work. We have provided a point-by-point response to the editor and reviewers, which is uploaded to as an attachment to this resubmission.

Attachment Submitted filename: Response to reviewers.docx

# Peer review: Decision Letter 1

**DOI:** 10.1371/journal.pone.0359723.r003

**Authors:** Elizabeth McGill

17 Sep 2026

Developing a systems-based programme theory for reducing night-time interactive electronic device use and improving sleep among adolescents

PONE-D-25-55895R1

Dear Dr. Rigby,

We’re pleased to inform you that your manuscript has been judged scientifically suitable for publication and will be formally accepted for publication once it meets all outstanding technical requirements.

Within one week, you’ll receive an e-mail detailing the required amendments. When these have been addressed, you’ll receive a formal acceptance letter and your manuscript will be scheduled for publication.

An invoice will be generated when your article is formally accepted. Please note, if your institution has a publishing partnership with PLOS and your article meets the relevant criteria, all or part of your publication costs will be covered. Please make sure your user information is up-to-date by logging into Editorial Manager at Editorial Manager® and clicking the ‘Update My Information' link at the top of the page. For questions related to billing, please contact billing support.

If your institution or institutions have a press office, please notify them about your upcoming paper to help maximize its impact. If they’ll be preparing press materials, please inform our press team as soon as possible -- no later than 48 hours after receiving the formal acceptance. Your manuscript will remain under strict press embargo until 2 pm Eastern Time on the date of publication. For more information, please contact onepress@plos.org.

Kind regards,

Elizabeth McGill

Academic Editor

PLOS One

Additional Editor Comments: Thank you for your comprehensive engagement with the reviewers' comments. As you point out, the manuscript is now longer as a result, but I think the revisions have strengthened it considerably. I look forward to seeing this published.

Reviewers' comments:

Reviewer’s Responses to Questions

**Comments to the Author**

Reviewer #2: All comments have been addressed

**********

2. Is the manuscript technically sound, and do the data support the conclusions?

Reviewer #2: (No Response)

**********

3. Has the statistical analysis been performed appropriately and rigorously?

Reviewer #2: (No Response)

**********

4. Have the authors made all data underlying the findings in their manuscript fully available?

The PLOS Data policy

Reviewer #2: (No Response)

**********

5. Is the manuscript presented in an intelligible fashion and written in standard English?

Reviewer #2: (No Response)

**********

Reviewer #2: (No Response)

**********

what does this mean?). If published, this will include your full peer review and any attached files.

If you choose “no”, your identity will remain anonymous but your review may still be made public.

**Do you want your identity to be public for this peer review?**  For information about this choice, including consent withdrawal, please see our Privacy Policy

Reviewer #2: No

**********

# Editor report: Acceptance letter

**DOI:** 10.1371/journal.pone.0359723.r004

**Authors:** Elizabeth McGill

PONE-D-25-55895R1

PLOS One

Dear Dr. Rigby,

I'm pleased to inform you that your manuscript has been deemed suitable for publication in PLOS One. Congratulations! Your manuscript is now being handed over to our production team.

At this stage, our production department will prepare your paper for publication. This includes ensuring the following:

* All references, tables, and figures are properly cited

* All relevant supporting information is included in the manuscript submission,

* There are no issues that prevent the paper from being properly typeset

You will receive further instructions from the production team, including instructions on how to review your proof when it is ready. Please keep in mind that we are working through a large volume of accepted articles, so please give us a few days to review your paper and let you know the next and final steps.

Lastly, if your institution or institutions have a press office, please let them know about your upcoming paper now to help maximize its impact. If they'll be preparing press materials, please inform our press team within the next 48 hours. Your manuscript will remain under strict press embargo until 2 pm Eastern Time on the date of publication. For more information, please contact onepress@plos.org.

You will receive an invoice from PLOS for your publication fee after your manuscript has reached the completed accept phase. If you receive an email requesting payment before acceptance or for any other service, this may be a phishing scheme. Learn how to identify phishing emails and protect your accounts at https://explore.plos.org/phishing.

If we can help with anything else, please email us at customercare@plos.org.

Thank you for submitting your work to PLOS One and supporting open access.

Kind regards,

PLOS One Editorial Office Staff

on behalf of

Dr Elizabeth McGill

Academic Editor

PLOS One
